# Country- and individual-level factors associated with healthy working life expectancy: A multinational longitudinal study

**DOI:** 10.1371/journal.pmed.1004676

**Published:** 2026-09-10

**Authors:** Jingjie Zhu, Bo Ye, Zeyu Huang, Huazhen You, Junjia Jiang, Shitong Yang, Junling Gao

**Affiliations:** 1 School of Public Health, Fudan University, Shanghai, China; 2 Huadong Hospital, Fudan University, Shanghai, China; Waymark, UNITED STATES OF AMERICA

## Abstract

**Background:**

To address labor force shortages associated with population aging, many countries have proposed raising the retirement age. As health is an important determinant of employment outcomes among older workers, it is important in this context to understand the individual- and country-level factors associated with healthy working life expectancy (HWLE), defined as the average number of years individuals can expect to remain healthy and employed from age 50.

**Methods and findings:**

This is a multinational prospective cohort study, leveraging data from six health and retirement cohorts. The study included 79,833 men and 95,485 women aged ≥50 years from 28 countries. Individual-level factors included demographics, socioeconomic status, lifestyle factors, and chronic diseases. Country-level factors comprised economic (GDP per capita, health expenditure, tax revenue, Gini index, ease of doing business), employment (flexible working Arrangements, labor force participation, equal workforce opportunities, hiring and firing practices) and governance (effectiveness, corruption control, political stability) metrics sourced from the World Bank. HWLE was estimated using discrete-time Markov multi-state models. Among the total participants, at age 50, the total life expectancy (TLE) was 33.59 years (95% CI [33.49, 33.69]), of which 10.98 years (95% CI [10.87, 11.08]) were HWLE. HWLE among women(9.82 years, 95% CI [9.68, 9.97]) was shorter than HWLE among men(12.36 years, 95% CI [12.22, 12.50]). Marriage, higher education, and healthy lifestyles were associated with longer HWLE, while chronic diseases reduced HWLE, with stroke showing the greatest reduction of 5.49 years (95% CI [4.94,6.04]). At the country level, higher health expenditures, GDP per capita, and tax revenue were associated with longer HWLE. Greater governance effectiveness, corruption control, and ease of doing business were associated with longer HWLE, whereas higher Gini index and political instability were associated with shorter HWLE. Higher levels of flexible working arrangements, labor force participation, equal workforce opportunities, and more favorable hiring and firing practices were consistently associated with longer HWLE and nHWLE and shorter nHnWLE. The main limitations were differences in follow-up periods across cohorts, relatively small samples in some countries, and incomplete global coverage, which may limit cross-country comparability and generalizability.

**Conclusions:**

In this multinational cohort, the findings revealed the associations of individual- and country-level factors with healthy working lives, underscoring the necessity for measures aimed at extending healthy working lives to align with country-specific social, economic and employment contexts. Besides, interventions targeting individual modifiable risk factors should be warranted through life-course.

## Author summary

**Why was this study done?:**

- No prior multinational study had quantitatively evaluated both individual and national factors associated with HWLE across diverse populations.

**What did researchers do and find?:**

- This study provides a comprehensive estimate of HWLE at age 50, and identifies national economic indicators, governance quality and employment-related conditions as contextual factors associated with HWLE, beyond individual factors like chronic diseases and lifestyle.

**What do these findings mean?:**

- Policies may consider integrating individual interventions (e.g., chronic disease prevention) with structural reforms (economic growth, governance) to extend healthy working life.
- Retirement systems and healthy-aging policies should consider evidence on healthy working capacity together with broader social and economic contexts.
- These findings should be interpreted with caution due to small sample sizes in some countries and limited global coverage.

## Abstract 3

Jingjie Zhu and Bo Ye examine healthy working life expectancy across nearly 175,000 adults aged 50 and over in 28 countries, finding that factors such as higher education, healthier lifestyles, and supportive economic, governance, and employment environments are associated with longer periods of healthy employment. Their study also highlights substantial differences by sex and health status, with chronic conditions, particularly stroke, linked to shorter healthy working lives.

## Introduction

Globally, accelerating population aging has led to a relative shortage of the productive labor force compared with a growing number of retirees [1]. In order to mitigate the labor force shortage with population aging, many countries have proposed raising the retirement age as a solution [2]. People are thus expected to stay in work longer than before [3]. Health is a strong determinant of work outcomes among older workers. Poor physical and mental health increases the risk of absenteeism, presenteeism, work disability, early retirement, and increased employer costs [4]. In this context, extending working life expectancy in a healthy state among older workers is important, which means not merely prolonging the years individuals spend in the workforce, but ensuring that these additional working years are accompanied by good health and functional capacity. It is crucial because it helps address the labor force shortages associated with population aging while simultaneously reducing the risk of mismatch between evolving retirement policies and individuals’ actual ability to work. By fostering a healthy working life, delayed retirement policies become more sustainable, benefiting both individuals through improved well-being and societies through sustained productivity and reduced healthcare burdens.

Previous studies have established healthy working life expectancy (HWLE) as a population indicator reflecting the average number of years a person is expected to remain healthy and in work from age 50 [5]. HWLE has been widely utilized to assess health status and working capacity across populations, particularly in the context of extended working lives [6–9]. A population-based study in England revealed that HWLE was only 9.42 years among people at age 50 years (10.94 years for men and 8.25 years for women) [9], which was lower than the UK State Pension age. Another study in China found that HWLE was only 6.87 years (8.06 years for men and 5.77 years for women) among people at age 50 years, which indicated male HWLE was also lower than the China State Pension age [10]. Previous studies have shown that individual-level factors, including gender, education, lifestyle, and chronic conditions, are associated with HWLE [6,7,9,11]. In addition, country-level factors, such as gross domestic product (GDP) per capita [12], national health expenditures [2], quality of governance [9], and the Gini index [7] are closely related to population health.

While previous research has established the influence of individual-level factors on HWLE, a systematic, multinational evaluation of country-level factors, particularly beyond simple wealth indicators, has been lacking. This study aims to fill this gap by quantifying the distinct associations of the country-level factors with HWLE. Specifically, we move beyond the general understanding that wealthier nations tend to have longer healthy working lives by dissecting the specific contributions of various economic indicators (e.g., health expenditure, tax revenue) and, crucially, employment-related conditions (e.g., flexible working arrangements, equal workforce opportunities), governance quality metrics (e.g., government effectiveness, control of corruption, political stability). This approach allows for a more nuanced understanding of how diverse national contexts are associated with healthy working life, providing actionable insights for policy development that extends beyond mere economic prosperity.

This study aimed to estimate HWLE among a multinational population aged 50 and older in 28 countries across three continents, and to examine the associations of individual-level and country-level factors with HWLE.

## Methods

### Study design and participants

This study used data of 6 harmonized cohorts from the Gateway to Global Aging Data (https://g2aging.org/): (1) the Health and Retirement Survey (HRS, 1992–2018); (2) the English Longitudinal Study of Ageing (ELSA, 2002–2019); (3) the Survey of Health, Ageing and Retirement in Europe (SHARE, 2004–2020); (4) the Korean Longitudinal Study of Aging (KLoSA, 2006–2018); (5) the Mexican Health and Aging Study (MHAS, 2001–2019); and (6) the China Health and Retirement Longitudinal Study (CHARLS, 2011–2018). The Global Aging Data have been specifically harmonized to allow comparability of the self-reported measures [13]. The detailed cohorts’ information was provided in S1 Text. Survey weights were not applied in the analyses due to inconsistent weight construction across cohorts. This study adheres to the Strengthening the Reporting of Observational Studies in Epidemiology (STROBE) guidelines (S1 Checklist) and the Guidelines for Accurate and Transparent Health Estimates Reporting (GATHER) statement (S2 Checklist).

### Outcomes

Self-reported health (SRH) was used as a health measure, which has proven to be a reliable and valid health indicator predicting healthcare utilization, current and future health problems, and mortality, and frequently used to study healthy life expectancy [14]. The previous studies also indicated SRH was equivalent to other health measures: the presence or absence of limiting long-standing illness [9] and the presence or absence of diagnosed chronic diseases [7] in calculating HWLE. Consistent with previous studies [9], health was defined as good if respondents reported good, very good, or excellent general health and poor if respondents reported fair, bad, very bad, or poor general health.

Work was defined as participation in paid work or self-employment within the month preceding the interview. According to participants’ health and work states in each wave, participants were classified into one of four alive health and work states (healthy and working: HW, healthy and not working: HnW, not healthy and working: nHW, and not healthy and not working: nHnW) or death, permitted transitions shown with arrows (Fig B in S1 Appendix).

### Age calculation

Multistate model used to estimate life expectancy not only needs the exact status of a condition but also needs the exact age at the specific status. Age of survivors was calculated as the difference between the first interview date and the date of birth. For age at death who died during follow-up was calculated by the difference between the specific time of death and birthday. Mortality information was obtained from exit and post-exit interviews conducted with proxy respondents who knew the deceased participants well, such as their spouse, child, or another close person (S2 Text). Partially missing time of death data were extrapolated from the closest interview date of the last wave and known vital status at this interview [9] and were determined as the mid of the time interval between two consecutive interviews [10].

### Individual-level factors

Based on previous studies [9,10], the following individual-level factors were included in this study: gender, educational attainment, marital status, health behaviors (smoking, drinking, and physical activity), and chronic diseases. Due to availability, hypertension, diabetes, cancer, lung disease, heart problems, stroke, and arthritis, which were available in all cohorts, were included in our analyses. All individual-level factors were self-reported when participants were first enrolled in the cohort. At baseline, all participants reported whether they had any doctor-diagnosed diseases, including hypertension, diabetes, cancer, lung disease, heart problems, stroke, or arthritis. The detailed definitions and coding of these individual-level factors are provided in S3 Text and can also be accessed from the Gateway to Global Aging Data (https://g2aging.org/vac/home).

### Country-level factors

In order to examine country-level factors associated with HWLE, based on availability of data, and previous literature on contextual factors considered to affect population health [7], the following country socio-economic indicators gained from the World Bank (https://databank.worldbank.org/home) were used: Flexible Working Arrangements, Labor Force Participation (% of adult population), Equal Workforce Opportunities, Hiring and Firing Practices, GDP per capita (constant 2015 US$), The Gini Index (reflecting the degree of inequality in income or consumption distribution), Current Health Expenditure (% of GDP), Tax Revenue (% of GDP), Ease of Doing Business, Government Effectiveness, Control of Corruption, and Political Stability and Absence of Violence/Terrorism (PSAVT). The detailed information was provided in S3 Text**.** All country-level factors were dichotomized into “high” and “low” groups. For the first four indicators, comparable annual historical data were unavailable; therefore, country-specific values from the TTDI 2024 assessment were assigned to participants according to their country of residence. For the remaining eight factors, data corresponding to the year in which each participant was first enrolled in the cohort were used. If data for that year were unavailable, values from the preceding year were used instead. For each factor, participants were subsequently classified into “high” and “low” groups according to the median of the assigned values in the pooled sample. Observations with values exactly equal to the median were allocated to the group that minimized the difference in sample size between the two groups.

### Statistical analysis

Given the dynamic nature of health and working state, a multi-state model was defined (Fig B in S1 Appendix), which permits transitions (shown with arrows) from any alive state to any state (including the same state and the dead state). Discrete-time Markov multistate models were used to estimate HWLE with standard errors using Interpolation of Markov Chains (IMaCh) software version 0.99s7 [15]. Based on the Markov assumption, the model assumes that an individual’s transition between states depends only on the current state and covariates. The transition probabilities were allowed to vary by age but were assumed to remain constant within each time interval. Considering the facts that participants of the cohorts were followed up at about two-year intervals, all estimations used biennial transition probability models with interpolation steps of size 24 months. The detailed information on the method has been described in previous studies [9,10]. A brief description was provided in S4 Text.

Firstly, life expectancies were calculated overall for all participants. In order to assess impacts of individual-level factors on life expectancies, we firstly calculated life expectancies for participants stratified by each individual-level factor, including gender, educational attainment, marital status, health behaviors (smoking, drinking, and physical activity), and chronic diseases (hypertension, diabetes, cancer, lung disease, heart problems, stroke, and arthritis), and then calculated the differences of life expectancies and 95% CI of the differences between levels of each individual-level factor. Similarly, to assess the associations of country-level factors with life expectancy and health expectancies, each participant was assigned the corresponding country-level value according to the procedure described above. Based on the distribution of the assigned values across all participants, each country-level factor was dichotomized at the median into high- and low-level groups. The corresponding estimates were then obtained for the two groups, and the differences between groups, together with their 95% CI, were calculated. The 95% CI of the difference was calculated based on the square root of the sum of variances under the assumption of independence [16]. For comparisons between groups, if the 95% CIs do not overlap, it suggests a statistically significant difference.

All analyses were conducted in IMaCh 0.99s7 [15] and R [17]. No formally registered prospective protocol or analysis plan was available. The study objectives and analytical approach were specified before the primary analyses were conducted, and no changes to the analytical approach were made based on data-driven findings or during peer review.

### Sensitivity analysis

To assess the robustness of the findings, we conducted a series of sensitivity analyses. First, to examine whether the results were sensitive to the interpolation interval used in the transition probability models, analyses were repeated using step sizes of 12 months (Method 1), 18 months (Method 2), and 36 months (Method 3). Second, to assess the potential impact of sample selection, we included the 11,465 individuals who had been excluded from the primary analysis due to missing individual-level covariates and re-estimated the results in the intact sample of 186,783 participants (Method 4). We also randomly selected 70% of the analytic sample and repeated the analyses in this sub-sample of 122,722 participants (Method 5). Third, to assess whether the findings were sensitive to the health definition, we used activities of daily living (ADL) as a functional definition of health. Health was defined as good if participants reported no difficulty in bathing, dressing, eating, getting in and out of bed, or walking across a room, and poor if difficulty was reported in at least one of these activities. Based on this alternative definition, analyses were repeated using a 24-month step (Method 6) and a 12-month step (Method 7).

### Ethics statement

This study was approved by the Ethics Committee of the School of Public Health, Fudan University (approval number: IRB#2021-TYSQ-03–108). Informed consent was obtained from participants in all six original cohort studies (CHARLS, ELSA, HRS, SHARE, MHAS, and KLoSA) before participation, in accordance with each study’s procedures. As the present analysis used de-identified secondary data, additional informed consent was not required.

### Use of artificial intelligence tools

ChatGPT was used solely to assist with English-language editing and improve the clarity and readability of the manuscript. All AI-assisted text was reviewed, revised, and verified by the authors. The authors take full responsibility for the final manuscript.

## Results

Using these harmonized data, we constructed a multinational dynamic cohort of 241,622 participants aged 50 years and older from 28 countries, which were included based on data availability. Among them, 54,101 were excluded due to being interviewed in only one wave; 154 were excluded due to missing information of age or birthdate, and 11,465 were excluded for missing information of individual-level factors (Fig A in S1 Appendix), so the final analytic sample included 175,318 participants. Information of the number of participants by waves was provided in Table A in S1 Appendix.

### Characteristics of participants and life expectancy across countries

The baseline characteristics of the overall and specific cohort participants in Table B in S1 Appendix. The average age of the overall sample was 63.91 years (SD: 9.68), with 54.5% of women. The mean follow-up duration for the overall sample was 8.22 years (SD: 5.58).

Among the total population, the TLE was 33.59 years (95% CI [33.49, 33.69]), of which 10.98 years (95% CI [10.87, 11.08]) were HWLE. HWLE differed according to the health and employment status at age 50. Individuals who were healthy and working at 50 years had a HWLE of 12.19 years (95% CI [12.11, 12.27]), representing 36.1% of their TLE (33.73 years, 95% CI [33.63, 33.83]) at age 50. Healthy but not working individuals at age 50 had a HWLE of 7.54 years (95% CI [7.42, 7.66]), equivalent to 22.5% of their TLE (33.56 years, 95% CI [33.47, 33.66]). Those who were unhealthy but working at age 50 had an expected HWLE of 8.79 years (95% CI [8.68, 8.90]), 26.5% of their TLE of 33.17 years (95% CI [33.05, 33.30]). For individuals who were both unhealthy and not working at age 50, the HWLE was 5.53 years (95% CI [5.43, 5.63]), accounting for 17.0% of their TLE of 32.49 years (95% CI [32.37, 32.62]). Detailed information is provided in Fig C and Table D in S1 Appendix. Cohort-stratified results are shown in Table E in S1 Appendix.

### Individual-level factors of life expectancies

Table 1 shows life expectancies in different health and employment states, stratified by demographic factors, lifestyle behaviors, and chronic diseases. Women had a higher TLE (35.29 years, 95% CI [35.16, 35.42]) than men (31.72 years, 95% CI [31.58, 31.87]). Conversely, men had a longer HWLE (12.36 years, 95% CI [12.22, 12.50]) than women (9.82 years, 95% CI [9.68, 9.97]). Married individuals had both a longer TLE (33.87 years, 95% CI [33.76, 33.99]) and HWLE (11.19 years, 95% CI [11.08, 11.30]) compared with unmarried individuals (TLE: 32.53 years, 95% CI [32.31, 32.74]; HWLE: 10.12 years, 95% CI [9.87, 10.30]). HWLE showed an upward trend with increasing educational attainment.

**Table 1:** Life expectancy in different states by demographics, lifestyle factors, and chronic diseases.

	N	TLE	HWLE	HnWLE	nHWLE	nHnWLE
**Overall**	175,318	33.59(33.49–33.69)	10.98(10.87–11.08)	12.20(12.11–12.29)	2.28(2.23–2.33)	8.13(8.06–8.20)
**Gender**						
Men	79,833	31.72(31.58–31.87)	12.36(12.22–12.50)	10.29(10.17–10.41)	2.39(2.32–2.46)	6.68(6.59–6.77)
Women	95,485	35.29(35.16–35.42)	9.82(9.68–9.97)	13.87(13.74–14.00)	2.19(2.12–2.26)	9.41(9.31–9.51)
**Education level**						
Low	65,789	33.38(33.23–33.54)	9.69(9.50–9.87)	11.68(11.53–11.83)	2.66(2.57–2.75)	9.36(9.23–9.48)
Intermediate	66,353	33.19(33.02–33.36)	10.14(9.98–10.30)	12.34(12.19–12.49)	2.35(2.26–2.43)	8.36(8.24–8.48)
High	43,176	34.37(34.17–34.57)	13.11(12.95–13.28)	13.23(13.05–13.42)	1.87(1.80–1.95)	6.15(6.03–6.27)
**Marital status**						
Married	130,872	33.87(33.76–33.99)	11.19(11.08–11.30)	12.53(12.43–12.64)	2.26(2.21–2.31)	7.89(7.81–7.96)
Other	44,446	32.53(32.31–32.74)	10.12(9.87–10.36)	11.12(10.94–11.31)	2.35(2.24–2.47)	8.93(8.77–9.10)
**Physical activity**						
No	103,033	32.49(32.37–32.62)	10.09(9.95–10.23)	11.55(11.44–11.67)	2.14(2.08–2.20)	8.72(8.62–8.81)
Yes	72,285	35.45(35.29–35.62)	12.02(11.88–12.16)	13.65(13.50–13.81)	2.47(2.39–2.54)	7.32(7.21–7.43)
**Drinking**						
No	87,178	33.58(33.44–33.73)	11.82(11.69–11.94)	12.85(12.72–12.98)	2.00(1.94–2.06)	6.92(6.83–7.01)
Yes	88,140	33.54(33.40–33.68)	9.82(9.66–9.98)	11.67(11.54–11.80)	2.68(2.60–2.77)	9.37(9.26–9.48)
**Smoking**						
Never	92,022	36.04(35.91–36.17)	11.33(11.19–11.47)	13.65(13.51–13.78)	2.15(2.09–2.22)	8.91(8.81–9.01)
Former	48,191	33.03(32.86–33.20)	11.50(11.31–11.70)	12.18(12.01–12.35)	2.13(2.04–2.22)	7.22(7.10–7.34)
Current	35,105	28.34(28.12–28.56)	9.66(9.45–9.87)	8.75(8.59–8.91)	2.65(2.54–2.75)	7.29(7.14–7.44)
**Arthritis**						
Yes	43,334	32.71(32.51–32.91)	8.24(8.01–8.48)	10.43(10.25–10.60)	3.13(3.00–3.25)	10.92(10.74–11.10)
No	131,984	33.81(33.69–33.92)	11.65(11.54–11.76)	12.86(12.76–12.97)	2.06(2.01–2.11)	7.24(7.16–7.31)
**Cancer**						
Yes	9,314	28.49(27.95–29.04)	8.69(8.08–9.29)	9.48(9.04–9.93)	2.41(2.14–2.67)	7.92(7.57–8.26)
No	166,004	33.87(33.77–33.97)	11.06(10.96–11.16)	12.37(12.28–12.47)	2.28(2.23–2.33)	8.16(8.09–8.23)
**Diabetes**						
Yes	19,893	29.03(28.71–29.34)	6.97(6.59–7.36)	8.20(7.96–8.44)	3.32(3.11–3.54)	10.52(10.25–10.80)
No	155,425	34.17(34.07–34.27)	11.33(11.23–11.44)	12.76(12.66–12.86)	2.20(2.15–2.24)	7.88(7.80–7.95)
**Heart problems**						
Yes	22,273	29.49(29.18–29.80)	7.05(6.63–7.47)	8.66(8.39–8.93)	3.06(2.82–3.29)	10.73(10.43–11.03)
No	153,045	34.26(34.15–36.37)	11.26(11.15–11.36)	12.87(12.77–12.97)	2.23(2.18–2.28)	7.91(7.83–7.98)
**Hypertension**						
Yes	64,341	32.45(32.28–32.62)	8.89(8.69–9.09)	11.02(10.86–11.17)	2.76(2.66–2.87)	9.78(9.64–9.92)
No	110,997	34.17(34.05–34.29)	11.82(11.71–11.94)	13.02(12.91–13.14)	2.10(2.05–2.16)	7.22(7.14–7.30)
**Stroke**						
Yes	6,761	26.74(26.09–27.38)	5.57(4.66–6.48)	6.92(6.46–7.38)	2.85(2.38–3.32)	11.40(10.71–12.08)
No	168,557	33.88(33.78–33.98)	11.07(10.96–11.17)	12.45(12.36–12.54)	2.28(2.23–2.32)	8.08(8.01–8.16)
**Chronic lung disease**						
Yes	10,132	27.67(27.20–28.14)	6.38(5.82–6.93)	6.99(6.65–7.32)	3.48(3.17–3.79)	10.83(10.41–11.25)
No	165,186	33.94(33.84–34.04)	11.15(11.05–11.25)	12.55(12.45–12.64)	2.23(2.18–2.28)	8.01(7.94–8.08)

Data are *n*, mean (95% CI).

Heart problems were defined as self-reported doctor-diagnosed heart-related conditions, including heart attack, coronary heart disease, angina, and other heart problems. TLE, total life expectancy; HWLE, healthy and working life expectancy; HnWLE, life expectancy on healthy and not working status; nHWLE, life expectancy on not healthy and working status; nHnWLE, life expectancy on not healthy and not working status.

**Fig 1:**
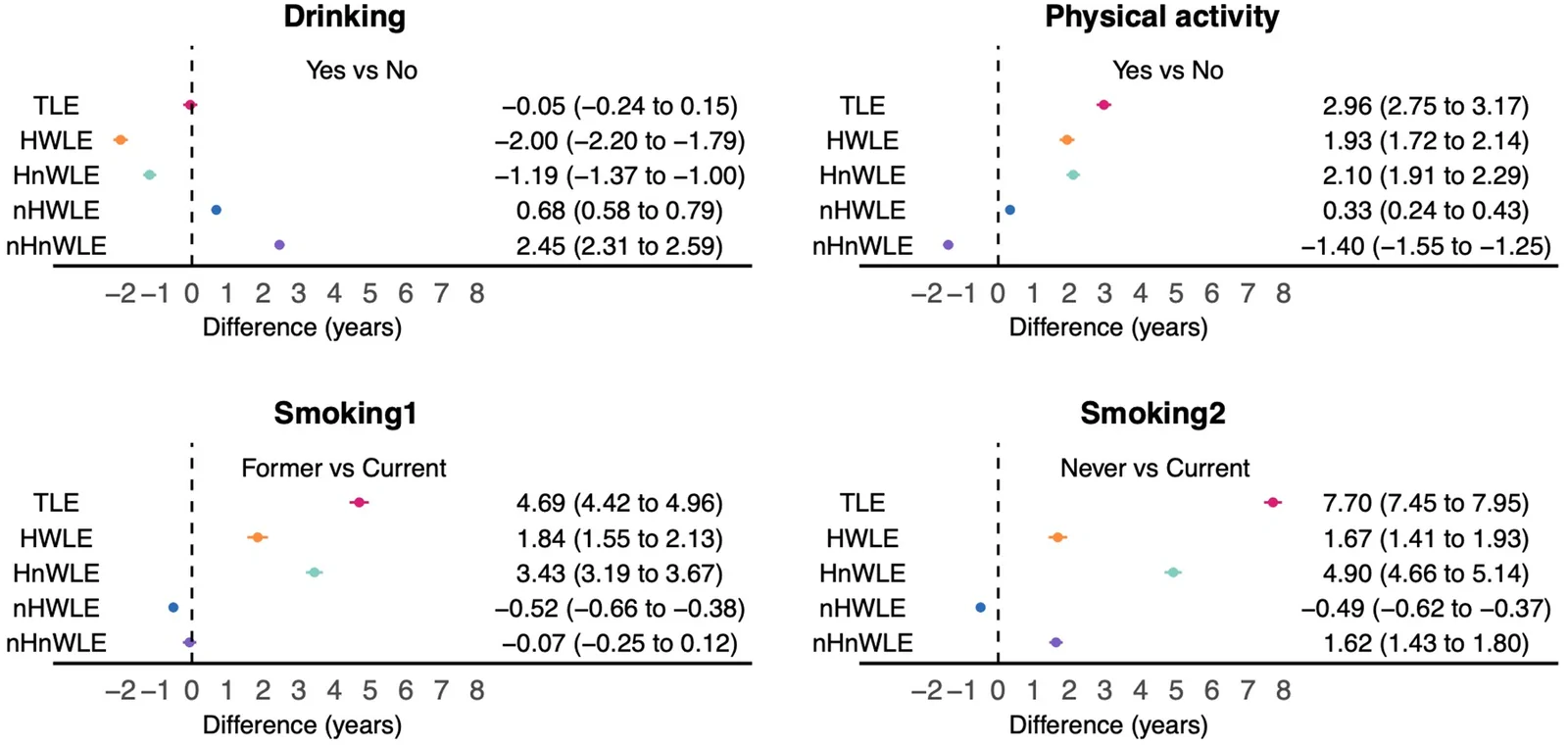
Health expectancies and life expectancy at age 50 according to lifestyle factors. Lifestyle factors include physical activity, smoking, and drinking. Estimates are derived from data on 175,318 individuals, with a mean follow-up of 8.22 years. Tails represent 95% CIs. TLE, total life expectancy; HWLE, healthy and working life expectancy; HnWLE, life expectancy on healthy and not working status; nHWLE, life expectancy on not healthy and working status; nHnWLE, life expectancy on not healthy and not working status.

Fig 1 depicts the associations between lifestyle factors and TLE and the four health expectancies. Physical activity was associated with a longer TLE (2.96 years, 95% CI [2.75, 3.17]) and a longer HWLE (1.93 years, 95% CI [1.72, 2.14]) at age 50. Although no significant difference in TLE was observed between drinkers (33.54 years, 95% CI [33.40, 33.68]) and non-drinkers (33.58 years, 95% CI [33.44, 33.73]), drinking was associated with a 2.00-year reduction in HWLE (95% CI [1.79, 2.20]; *p* < 0.05). Compared with current smokers, both former smokers and never smokers had higher TLE and HWLE. Specifically, former smokers had a 4.69-year higher TLE (95% CI [4.42, 4.96]; *p* < 0.05), and never smokers had a 7.70-year higher TLE (95% CI [7.45, 7.95]; *p* < 0.05). Former smokers had a 1.84-year higher HWLE (95% CI [1.55, 2.13]; *p* < 0.05), and never smokers had a 1.67-year higher HWLE (95% CI [1.41, 1.93]; *p* < 0.05).

**Fig 2:**
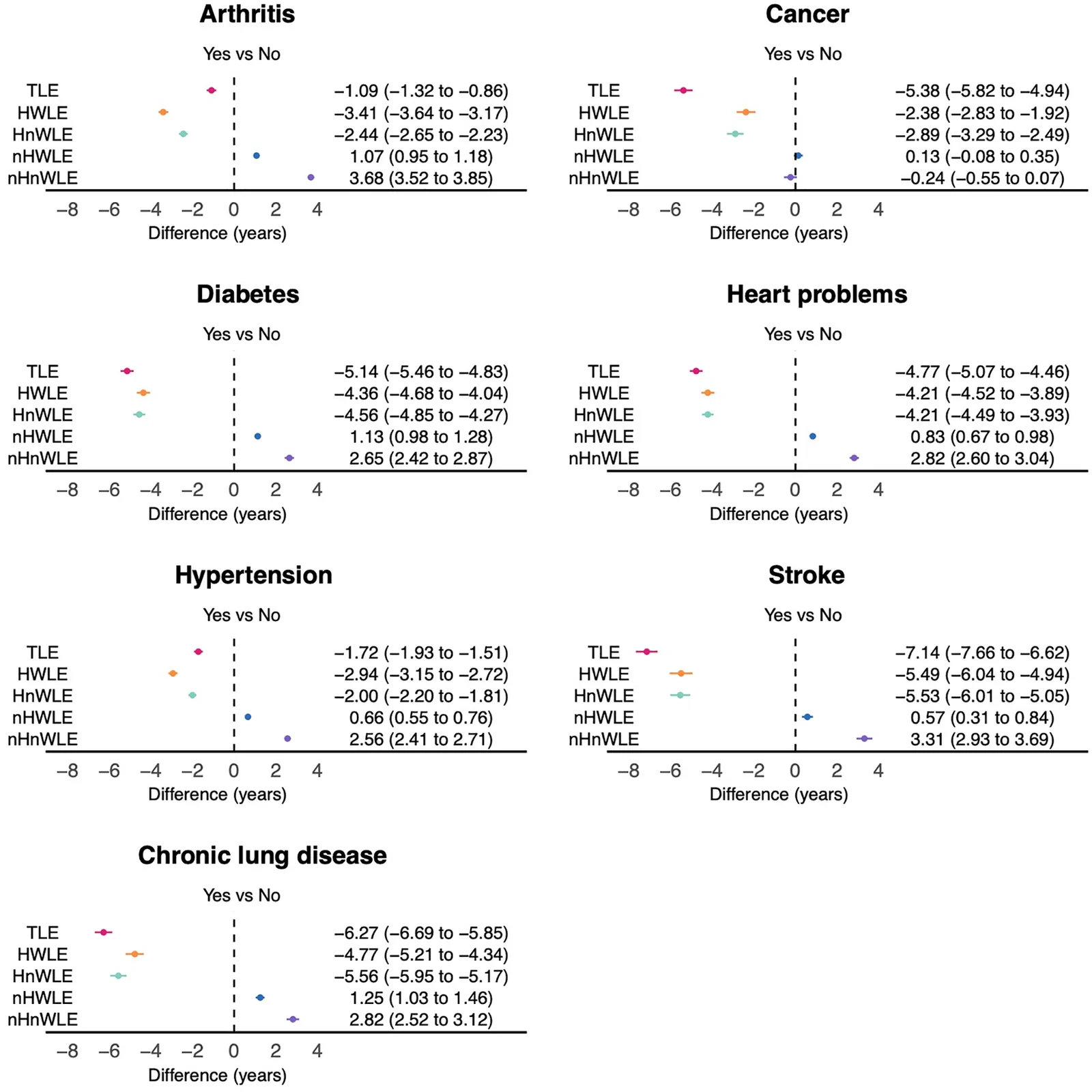
Health expectancies and life expectancy at age 50 according to chronic diseases. Chronic diseases include stroke, chronic lung disease, diabetes, cancer, heart disease, hypertension, and arthritis. Estimates are derived from data on 175,318 individuals, with a mean follow-up of 8.22 years. Tails represent 95% CIs. Heart problems were defined as self-reported doctor-diagnosed heart-related conditions, including heart attack, coronary heart disease, angina, and other heart problems. TLE, total life expectancy; HWLE, healthy and working life expectancy; HnWLE, life expectancy on healthy and not working status; nHWLE, life expectancy on not healthy and working status; nHnWLE, life expectancy on not healthy and not working status.

In addition, Fig 2 presents the associations between chronic diseases and TLE and the four health expectancies. Stroke, chronic lung disease, diabetes, cancer, heart disease, hypertension, and arthritis resulted in varying degrees of reduction in TLE and HWLE, in descending order of magnitude. For example, participants with stroke had a 7.14-year lower TLE (95% CI [6.62, 7.66]; *p* < 0.05) and 5.49-year lower HWLE (95% CI [4.94, 6.04]; *p* < 0.05) compared with those without stroke.

### Country-level factors of life expectancies

As shown in Table F in S1 Appendix, the estimated life expectancies varied substantially across countries. However, given potential differences in cohort representativeness and mortality ascertainment across countries, these absolute estimates should be interpreted cautiously.

**Fig 3:**
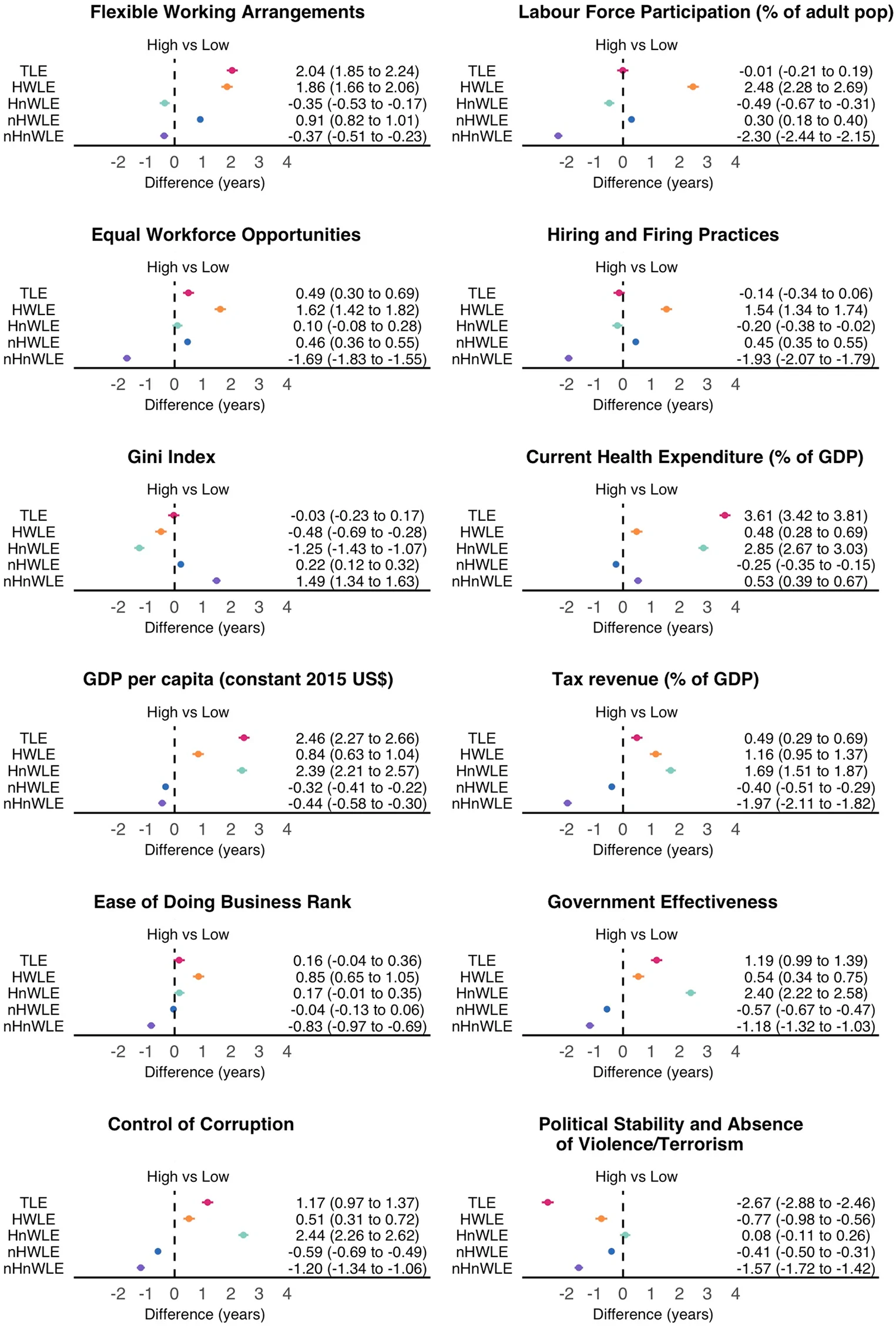
Health expectancies and life expectancy at age 50 across 33 countries according to country-level determinants. Twelve country-level determinants: Flexible Working Arrangements, Labor Force Participation (% of adult population), Equal Workforce Opportunities, Hiring and Firing Practices, The Gini index, Current health expenditure (% of GDP), GDP per capita (constant 2015 US$), Tax revenue (% of GDP), Ease of Doing Business Rank, Government Effectiveness, Control of Corruption, and Political Stability and Absence of Violence/Terrorism(PSAVT). Tails represent 95% CIs. GDP, gross domestic product. TLE, total life expectancy; HWLE, healthy and working life expectancy; HnWLE, life expectancy on healthy and not working status; nHWLE, life expectancy on not healthy and working status; nHnWLE, life expectancy on not healthy and not working status.

Table 2 and Fig 3 present the associations between national economic indicators and TLE and the four health expectancies. Individuals in countries with high national health expenditure had a higher TLE and HWLE than those in countries with low national health expenditure. The difference in TLE (DTLE) was 3.61 years (95% CI [3.42, 3.81]; *p* < 0.05), and the difference in HWLE (DHWLE) was 0.48 years (95% CI [0.28, 0.69]; *p* < 0.05). Similarly, higher GDP per capita (DTLE:2.46 years, 95% CI [2.27, 2.66], *p* < 0.05; DHWLE: 0.84 years, 95% CI [0.63, 1.04], *p* < 0.05) and higher tax revenue (DTLE:0.49 years, 95% CI [0.29, 0.69], *p* < 0.05; DHWLE:1.16 years, 95% CI [0.95, 1.37]; *p* < 0.05) were associated with longer TLE and HWLE. By contrast, a high Gini index was associated a 0.48-year shorter HWLE (95% CI [0.28, 0.69]; *p* < 0.05) compared with countries with a low Gini index, whereas populations in countries with higher rankings in ease of doing business had a 0.85-year longer HWLE (95% CI [0.65, 1.05]; *p* < 0.05) than those in countries with lower rankings. No significant associations were observed between the Gini index or ease of doing business rankings and TLE.

**Table 2:** Life expectancy in different states by country-level factors.

	N	TLE	HWLE	HnWLE	nHWLE	nHnWLE
**Flexible Working Arrangements**						
Low	86,658	32.61(32.47–32.75)	10.06(9.92–10.21)	12.39(12.26–12.52)	1.84(1.77–1.90)	8.32(8.22–8.42)
High	88,659	34.65(34.51–34.79)	11.92(11.78–12.05)	12.04(11.91–12.17)	2.75(2.68–2.82)	7.94(7.85–8.04)
**Labor Force Participation (% of adult population)**						
Low	80,975	33.60(33.44–33.75)	9.60(9.43–9.76)	12.38(12.24–12.52)	2.14(2.05–2.24)	9.47(9.35–9.59)
High	94,343	33.58(33.46–33.71)	12.08(11.95–12.20)	11.89(11.78–12.01)	2.44(2.38–2.50)	7.18(7.10–7.26)
**Equal Workforce Opportunities**						
Low	85,148	33.29(33.14–33.44)	10.13(9.97–10.28)	12.11(11.97–12.24)	2.02(1.95–2.09)	9.03(8.92–9.14)
High	90,170	33.78(33.65–33.91)	11.75(11.62–11.88)	12.21(12.09–12.33)	2.48(2.42–2.55)	7.34(7.25–7.43)
**Hiring and Firing Practices**						
Low	87,488	33.65(33.50–33.80)	10.17(10.01–10.32)	12.26(12.12–12.40)	2.03(1.95–2.10)	9.19(9.09–9.31)
High	87,830	33.52(33.39–33.64)	11.71(11.58–11.84)	12.06(11.94–12.18)	2.48(2.42–2.54)	7.26(7.18–7.35)
**Gini Index**						
Low	76,679	33.55(33.41–33.69)	11.33(11.20–11.47)	12.74(12.62–12.87)	2.18(2.13–2.24)	7.29(7.20–7.38)
High	98,639	33.52(33.38–33.66)	10.85(10.70–11.00)	11.50(11.37–11.62)	2.40(2.32–2.48)	8.78(8.68–8.88)
**Current Health Expenditure (% of GDP)**						
Low	85,177	31.93(31.79–32.06)	10.73(10.59–10.86)	10.92(10.80–11.03)	2.40(2.34–2.47)	7.88(7.79–7.98)
High	90,141	35.54(35.40–35.68)	11.21(11.05–11.36)	13.76(13.63–13.90)	2.15(2.08–2.22)	8.42(8.31–8.52)
**GDP per capita (constant 2015 US$)**						
Low	89,476	32.35(32.21–32.50)	10.54(10.40–10.68)	11.02(10.89–11.14)	2.43(2.36–2.50)	8.37(8.27–8.47)
High	85,842	34.82(34.68–34.95)	11.38(11.22–11.53)	13.40(13.27–13.53)	2.11(2.04–2.18)	7.93(7.83–8.03)
**Tax revenue (% of GDP)**						
Low	87,615	33.27(33.12–33.42)	10.46(10.29–10.63)	11.10(10.96–11.23)	2.55(2.45–2.64)	9.17(9.05–9.29)
High	87,703	33.76(33.63–33.89)	11.62(11.49–11.74)	12.79(12.67–12.91)	2.15(2.10–2.20)	7.20(7.12–7.28)
**Ease of Doing Business Rank**						
Low	86,581	33.49(33.33–33.64)	10.54(10.40–10.69)	12.05(11.91–12.18)	2.30(2.23–2.37)	8.60(8.49–8.71)
High	88,737	33.64(33.51–33.77)	11.40(11.26–11.53)	12.22(12.10–12.34)	2.26(2.20–2.32)	7.77(7.68–7.86)
**Government Effectiveness: Estimate**						
Low	86,661	32.88(32.73–33.03)	10.64(10.49–10.79)	10.84(10.70–10.97)	2.60(2.52–2.67)	8.80(8.69–8.91)
High	88,657	34.07(33.94–34.20)	11.18(11.04–11.32)	13.23(13.11–13.36)	2.03(1.96–2.09)	7.63(7.54–7.72)
**Control of Corruption: Estimate**						
Low	87,334	32.89(32.74–33.04)	10.66(10.51–10.80)	10.82(10.69–10.95)	2.61(2.53–2.68)	8.81(8.70–8.92)
High	87,984	34.06(33.93–34.19)	11.17(11.03–11.31)	13.26(13.14–13.38)	2.02(1.96–2.08)	7.61(7.52–7.71)
**Political Stability and Absence of Violence/Terrorism: Estimate**						
Low	85,856	32.50(32.37–32.64)	10.58(10.43–10.74)	12.17(12.04–12.29)	2.09(2.02–2.17)	7.66(7.56–7.76)
High	89,462	35.17(35.02–35.33)	11.36(11.22–11.49)	12.09(11.95–12.22)	2.50(2.44–2.56)	9.23(9.11–9.35)

Data are *n*, mean (95% CI).

TLE, total life expectancy; HWLE, healthy and working life expectancy; HnWLE, life expectancy on healthy and not working status; nHWLE, life expectancy on not healthy and working status; nHnWLE, life expectancy on not healthy and not working status. GDP, gross domestic product.

Regarding governance indicators, individuals in countries with high levels of governance effectiveness (DTLE:1.19 years, 95% CI [0.99, 1.39], *p* < 0.05; DHWLE:0.54 years, 95% CI [0.34, 0.75], *p* < 0.05), control of corruption (DTLE:1.17 years,95% CI [0.97, 1.37], *p* < 0.05; DHWLE:0.51 years, 95% CI [0.31, 0.72], *p* < 0.05), and political stability (DTLE:2.67 years, 95% CI [2.46, 2.88], *p* < 0.05; DHWLE:0.77 years, 95% CI [0.56, 0.98], *p* < 0.05) had better TLE and HWLE compared with those in countries with lower levels of these governance indicators.

Higher levels of all four employment-related indicators were consistently associated with longer HWLE and nHWLE and shorter nHnWLE. Specifically, individuals in countries with higher labor force participation had a 2.48-year longer HWLE (95% CI [2.28, 2.69]; *p* < 0.05), a 0.30-year longer nHWLE (95% CI [0.18, 0.40]; *p* < 0.05), a 0.49-year shorter HnWLE (95% CI [0.31, 0.67]; *p* < 0.05), and a 2.30-year shorter nHnWLE (95% CI [2.15, 2.44]; *p* < 0.05). Higher scores for hiring and firing practices showed a similar pattern, being associated with a 1.54-year longer HWLE (95% CI [1.34,1.74]; *p* < 0.05), a 0.45-year longer nHWLE (95% CI [0.35, 0.55]; *p* < 0.05), a 0.20-year shorter HnWLE (95% CI [0.02, 0.38]; *p* < 0.05), and a 1.93-year shorter nHnWLE (95% CI [1.79, 2.07]; *p* < 0.05). Compared with individuals in countries with lower levels of flexible working arrangements, those in countries with higher levels had a 2.04-year longer TLE (95% CI [1.85, 2.24]; *p* < 0.05) and significantly longer HWLE and nHWLE (DHWLE:1.86 years, 95% CI [1.66, 2.06], *p* < 0.05; DnHWLE:0.91 years, 95% CI [0.82, 1.01], *p* < 0.05). Similarly, higher levels of equal workforce opportunities were associated with longer TLE, HWLE, and nHWLE. (DTLE:0.49 years, 95% CI [0.30, 0.69], *p* < 0.05; DHWLE:1.62 years, 95% CI [1.42, 1.82], *p* < 0.05; DnHWLE:0.46 years, 95% CI [0.36, 0.55], *p* < 0.05).

### Sensitivity analyses

As shown in Table G and Fig D in S1 Appendix, the results from sensitivity analyses across transition probability models with different step sizes were consistent. Additionally, sensitivity analyses conducted with an intact sample of 186,783 individuals and a randomly selected 70% sub-sample (122,722 individuals) showed no statistically significant discrepancies when compared with the results of the primary analysis. Results obtained using an alternative health measure (ADL). The results showed that defining health as the absence of difficulty with ADL led to higher HWLE overall (11.72 years, 95% CI [11.63, 11.81], *p* < 0.05) and lower estimates for time spent not healthy and in work (1.46 years, 95% CI [1.43, 1.49], *p* < 0.05).

## Discussion

Using multinational quantitative data, this study estimated TLE and HWLE for individuals at age 50 from 28 countries and the associations of demographic factors, lifestyle behaviors, prevalent chronic diseases, and national disparities with TLE and HWLE. Poor physical and mental health increase the risk of absenteeism, presenteeism, work disability, early retirement, and increased employer costs [4]. At age 50, many individuals are in mid-to-late career stages and may begin to experience age-related health problems that affect work capacity and productivity [5,9,10]. Estimating HWLE at age 50 offers several advantages: it provides a comprehensive view of population health and labor-force participation, informs the feasibility of policies to prolong working lives, and facilitates comparison with findings from other studies. The results corroborate previous findings from England [9], Australia [11], Europe [18], and China [10], indicating that inequalities in HWLE persist across socioeconomic groups. This suggests that the capacity to remain both healthy and employed is not equally distributed across populations. In particular, individuals with lower socioeconomic status, poorer health, and chronic conditions may have fewer years available to remain in healthy work. This finding suggestsmay highlight the importance of considering older adults’ health and work capacity when evaluating retirement policies. For example, in the United States, HWLE was 10.69 years in our study, whereas the current full retirement age in the Social Security system is 67 for people born in 1960 or later [19]. This comparison is illustrative rather than a formal analysis of retirement systems, but it highlights the potential risk that some older adults may be expected to remain in work longer than their healthy working capacity would allow.

Importantly, this study systematically quantified and compared the associations between national economic status, governance quality, employment-related factors and both TLE and HWLE across a diverse multinational population. While the general notion that wealthier countries tend to have higher HWLE is acknowledged, our study provides novel insights by dissecting the specific contributions of various economic indicators and governance metrics, moving beyond a simplistic correlation with overall wealth. For instance, we found that while GDP per capita and national health expenditure are pivotal for longer TLE and HWLE, tax revenue emerged as a distinct factor more closely linked to maintaining a healthy workforce. This suggests that the allocation and generation of public funds, rather than just the overall economic output, play a critical role in supporting healthy working lives. Furthermore, the significant associations of governance quality metrics—including government effectiveness, control of corruption, and political stability—with both TLE and HWLE represent a crucial novel contribution. These findings suggest that robust institutional frameworks, transparent governance, and a stable political environment are not merely proxies for economic development but exert an independent influence on population health and the ability to maintain a healthy workforce. For instance, effective governance can ensure efficient allocation of health resources, implement effective public health programs, and foster a stable environment conducive to both economic activity and individual well-being, thereby directly supporting longer and healthier working lives. This nuanced understanding of how specific economic and governance dimensions, rather than just aggregate wealth, influence HWLE provides a deeper, more actionable perspective for policy interventions.

The relationships between macro-level per capita income, health expenditures, and population health have been well established [20,21]. Even within different regions of the same country, GDP disparities can give rise to health inequalities [22]. In several developed countries, the presence of diminishing marginal returns on health outcomes makes it increasingly difficult to achieve substantial gains in life expectancy beyond a certain level [23]. Conversely, in many low- and middle-income countries (LMICs), national health expenditure continues to show a significant inverse correlation with Disability-Adjusted Life Years (DALY) and a positive correlation with healthy life expectancy [20]. For example, China as one of the world’s most populous LMICs undergoing rapid population aging—exhibits marked interregional economic imbalance that have produced substantial regional disparities in HWLE [10]. This underscores the inadequacy of a “one-size-fits-all” retirement age policy [10]. In contrast. policies such as promoting phased retirement programs, enhancing fair hiring practices, expanding employment opportunities, and offering flexible work arrangements would be feasible approaches. Previous research has shown that augmenting health spending through increased taxation from tobacco and alcohol is a viable strategy to improve health outcomes, which can be reinvested in dynamic sectors, thereby promoting health, employment, and economic development [24–26].

Beyond economic factors, our results reveal associations of governance quality, specifically, government effectiveness, control of corruption, and political stability with both TLE and HWLE. These findings align with previous research suggesting that improvements in government effectiveness, reductions in corruption, and enhanced political stability are plausibly linked to better population health outcomes [20,27,28], which may be explained by the positive association between robust national governance and improved health service delivery.

Our findings further indicate that national employment-related conditions may shape how later life is distributed across health and work states. Higher levels of flexible working arrangements, labor force participation, equal workforce opportunities, and more favorable hiring and firing practices were consistently associated with longer HWLE and nHWLE and shorter nHnWLE. These patterns suggest that more inclusive and adaptable employment environments may enable older adults, including those experiencing health limitations, to remain economically active for longer. However, a longer nHWLE should not necessarily be interpreted as a favorable outcome. It may reflect effective workplace flexibility and accommodation that allow people with health limitations to continue working, but it may also indicate financial pressure, inadequate retirement protection, or presenteeism among workers in poor health. These findings should therefore be interpreted in light of each country’s specific labor-market conditions, social protection systems, and institutional context. Policies should aim not merely to prolong employment, but to create equitable and flexible working conditions that allow employment to remain compatible with individuals’ health and functional capacity.

Chronic diseases are increasingly recognized as a major global health burden, contributing substantially to mortality while profoundly affecting work capacity and overall health in an aging workforce [29]. Previous studies estimating HWLE from the perspective of chronic diseases have shown that individuals in Organization for Economic Cooperation and Development countries and China often continue to work for extended periods despite living with hypertension and arthritis [6,7]. Our findings, based on self-reported health measures, also show that stroke and chronic lung disease are associated with lower TLE and HWLE, indicating controlling for risk factors leading to stroke and chronic lung disease among adults may benefit to increasing TLE and HWLE.

Although this study pooled data from 28 countries to identify overall associations, the study design and the limited numbers of disease cases and state transitions in some countries restricted our ability to conduct detailed country-specific analyses of the relationship between individual health conditions and HWLE. Larger country-specific samples are needed to determine whether the associations between chronic diseases and working-life expectancy vary across countries. Such analyses may clarify how differences in healthcare systems, chronic disease management, public health interventions, and workplace accommodations modify the impact of chronic conditions on the ability to remain healthy and employed.

Furthermore, cross-country differences were evident not only in HWLE but also in HnWLE, nHWLE, and nHnWLE, suggesting that similar levels of TLE or HWLE may arise from substantially different health–employment trajectories. A relatively long HnWLE may reflect earlier labor-force exit, longer survival after retirement, or stronger financial protection outside paid employment. By contrast, a longer nHWLE indicates that individuals spend a considerable period working while in poor health. This pattern may reflect employment arrangements that enable people with health limitations to remain economically active, but it may also indicate financial necessity, inadequate retirement protection, or presenteeism. A relatively long nHnWLE represents the combined burden of poor health and labor-force non-participation. These differences may be shaped by chronic disease burden, retirement and disability systems, pension adequacy, labor-market conditions, and workplace support. Understanding these country-specific patterns can provide deeper insight into how national policies and institutional contexts influence the distribution of later life across different health and employment states. Accordingly, HWLE should be interpreted alongside HnWLE, nHWLE, and nHnWLE rather than as an isolated indicator of population health and employment.

Notably, healthy behaviors such as never smoking, and regular physical activity were associated with substantial higher TLE and HWLE, highlighting potentially modifiable factors for health outcomes. Previous studies have demonstrated that smoking and insufficient physical activity are associated with reduced life expectancy and work ability [30–32]. This context helps to explain our findings, which suggest that the absence of smoking and engagement in physical activity are important determinants of longer TLE and HWLE. In response to population aging, feasible strategies include investments in health management and disease prevention—these not only promote a healthier society but also yield returns in strengthening the workforce and extending healthy lifespans, thereby improving the economy.

Finally, consistent with previous studies [6–9], women had longer TLE but shorter HWLE than men. This disparity can be partly explained by earlier retirement ages for women in many countries and by gender-based factors that limit women’s opportunities to remain in the workforce [33]. Policies encouraging women to delay retirement and expand employment options have substantial potential. Additionally, higher education levels are positively correlated with better health, productivity, and employment opportunities [34] which supports our finding that greater educational attainment is associated with longer HWLE. Thus, ensuring universal access to education could be a benefit strategy for enhancing TLE and HWLE.

The strengths of our study include its multinational population-based design, long-term follow-up, examination of country-level factors associated with and HWLE. However, several limitations should be acknowledged. First, the baseline and follow-up periods of the harmonized cohorts varied across countries and regions, and the number of waves also differed across datasets, which may bias cross-country comparisons because health and working states may be influenced by the temporal and policy contexts at baseline and follow-up. Second, although the overall sample size across countries was large, this was not a global sample, and the sample size in some countries was relatively small, which may limit the generalizability of the findings. This is particularly important for transition-based multistate models, because the estimates depend on the number of observed transitions between states. When the sample size is small in some countries, the number of specific transitions may be limited, which can lead to unstable transition probability estimates and wider confidence intervals. Third, SRH-based HWLE estimates are comparable to those derived from measures of limiting long-standing illness or diagnosed chronic conditions [9,10], but self-reported measures of health and working status are susceptible to measurement error. The definition of work was inclusive and did not differentiate full-time workers from people who work part time. Moreover, work ability limitations are an outcome of poor health. Some individuals may perceive themselves as good health, yet their work ability is limited. This discrepancy could lead to misestimation of HWLE estimates. Fourth, information on doctor-diagnosed diseases was collected at baseline and not updated during follow-up. Changes in disease status over time, including the onset or remission of diseases, may have affected the estimation of transitions. Fifth, mortality ascertainment bias may have affected our estimates. If some deceased participants were not identified and were instead treated as lost to follow-up, mortality rates may have been underestimated, which may have led to overestimation of TLE and HWLE. Sixth, the addition of new participants to each cohort at different times may introduce cohort effects, as disease incidence and prevalence change over time, potentially biasing our estimates. Seventh, sample weights were not applied in our analyses, due to the multi-cohort design and inconsistent methods of the construction of weights across cohorts. Additionally, throughout the life course, individual intrinsic capacity, relevant environmental characteristics, and the interactions between individuals and their environments are key determinants of healthy aging and functional ability [12,29]. However, due to methodological constraints, we were unable to clarify the effects of these interactions on HWLE, which represents an important area for future research. Finally, we acknowledge that the estimated TLE and HWLE for some countries, particularly Switzerland and Austria, were higher than estimates from official national statistics. This discrepancy suggests that cohort-based estimates may be subject to country-specific biases arising from differences in sampling frameworks, follow-up procedures, mortality ascertainment, and population representativeness. Importantly, we cannot determine whether the magnitude of this potential bias is uniform across countries. Therefore, differential bias cannot be excluded, and comparisons of absolute HWLE values between countries should be interpreted cautiously. Our findings should primarily be interpreted as describing associations between individual- and country-level factors and HWLE within the study framework rather than providing definitive rankings of countries according to absolute HWLE..

Nevertheless, this study may help inform strategies to extend healthy working life by identifying factors associated with shorter HWLE at both the individual and country levels. For example, the associations of chronic diseases with lower TLE and HWLE highlight the importance of chronic disease prevention and management, while the associations with health system investment, social inequality, and governance indicators suggest that policy and institutional contexts may shape opportunities to remain healthy and employed at older ages. Health promotion programs focused on preventing chronic diseases (i.e., stroke and chronic lung disease), controlling tobacco use, and promoting physical activity have the potential to extend both life expectancy and healthy working years. At the national level, strategies to boost economic development and improve governance quality, such as increasing tobacco taxes to fund sports facility construction may be beneficial to population health; improving ease of doing business may provide more job opportunity. In summary, a variety of multilevel interventions are warranted to extend the TLE and HWLE. At the individual level, prevention and effective management of chronic diseases, particularly diabetes, stroke, and chronic lung disease, along with healthier lifestyles, may help preserve both health and work capacity. At the employment level, creating more equitable, inclusive, and flexible working environments may help individuals remain economically active without requiring them to work beyond their health and functional capacity. At the policy level, greater investment in health systems, efforts to reduce social inequality, and stronger governance may provide the structural conditions needed to support longer and healthier working lives.

## Supporting information

S1 Checklist STROBE Checklist. Completed STROBE checklist for this study. Source: STROBE Statement (https://www.strobe-statement.org/), licensed under CC BY 4.0.
(DOCX)

S2 Checklist GATHER Checklist. Completed GATHER checklist for this study. Source: Guidelines for Accurate and Transparent Health Estimates Reporting (GATHER) (https://www.who.int/data/gather), licensed under CC BY 4.0.
(DOCX)

S1 Text Cohort profiles used in this study. (DOCX)

S2 Text Methods to gain mortality information: Exit and Post-exit Interviews. (DOCX)

S3 Text Measures of individual-level and country-level factors of HWLE. (DOCX)

S4 Text Methods of HWLE calculation. (DOCX)

S1 Appendix Figs A–D and Tables A–G. **Fig A.** Flowchart of participants. **Fig B.** Hypothetical transitions of healthy and working status. **Fig C.** Life expectancy in different states at age 50 by starting state. **Fig D.** Results of sensitivity analyses. **Table A.** Number of individuals in each wave across six databases. **Table B.** Sample characteristics across six databases. **Table C.** Sample characteristics of SHARE participants by country. **Table D.** Life expectancy in different states at age 50 by starting state. **Table E.** Life expectancy in different states across cohorts. **Table F.** Life expectancy in different states across countries. **Table G.** Results of sensitivity analyses by including all missing data, sampling randomly, using different interpolations and health definitions.
(DOCX)

## Abbreviations

ADL: activities of daily living
CHARLS: China Health and Retirement Longitudinal Study
DALY: Disability-Adjusted Life Years
DHWLE: difference in HWLE
DTLE: difference in TLE
ELSA: English Longitudinal Study of Ageing
GATHER: Guidelines for Accurate and Transparent Health Estimates Reporting
GDP: gross domestic product
HRS: Health and Retirement Survey
HWLE: healthy working life expectancy
IMaCh: Interpolation of Markov Chains
KLoSA: Korean Longitudinal Study of Aging
LMICs: low- and middle-income countries
MHAS: Mexican Health and Aging Study
PSAVT: Political Stability and Absence of Violence/Terrorism
SHARE: Survey of Health, Ageing and Retirement in Europe
SRH: self-reported health
STROBE: Strengthening the Reporting of Observational Studies in Epidemiology
TLE: total life expectancy
