# Supplementary material for: Country- and individual-level factors associated with healthy working life expectancy: A multinational longitudinal study
Source: PLoS Med. 2026 Sep 10;23(9):e1004676. doi: 10.1371/journal.pmed.1004676 (PMC13561363; doi:10.1371/journal.pmed.1004676)
Supplement: Supplementary file 3 [file pmed.1004676.s003.docx]

**S1 Text. Cohort profiles used in this study**

1) **the Health and Retirement Survey (HRS)** is a nationally representative longitudinal survey of more than 37 000 individuals over age 50 in 23 000 households in the USA. The survey has been fielded every 2 years since 1992. This study uses the data of 14 waves from 1992-2018^1^;
2) **the English Longitudinal Study of Ageing (ELSA)** is a panel study of a representative cohort consisted of 11 391 men and women living in England aged ≥50 years. The study commenced in 2002, and the sample has been followed up every 2 years. Until 2019, the ELSA have conducted 9 waves, this study used wave 1,2,4,5,6 due to health information was unavailable in wave 3,7,8,9^2^;
3) **the Survey of Health, Ageing and Retirement in Europe** (SHARE) was conducted among more than 150 000 adults aged ≥50 years in 27 Europe countries (Austria, Germany, Sweden, Netherlands, Spain, Italy, France, Denmark, Greece, Switzerland, Belgium, Czech Republic, Poland, Luxembourg, Hungary, Portugal, Slovenia, Estonia, Croatia, Lithuania, Bulgaria, Cyprus, Finland, Latvia, Malta, Romania, and Slovakia) and Israel. The study commenced in 2004, and the sample has been followed up every 2 years. Until 2020, 8 waves surveys were conducted, this study used 7 waves data due to data of wave 3 is unavailability^3^;
4) **the Korean Longitudinal Study of Aging (KLoSA)** was conducted among 10 254 adults aged ≥50 years in South Korea. The study commenced in 2006, and the sample has been followed up every 2 years. From 2006-2018, 7 waves were conducted^4^;
5) **the Mexican Health and Aging Study (MHAS)** was conducted among 15 186 adults aged ≥50 years in Mexico since 2001; The sample has been followed up every 2 years. until 2019, 5 waves surveys were conducted^5^;

6) **the China Health and Retirement Longitudinal Study (CHARLS)** was conducted among 17 708 aged ≥50 years in China; The study commenced in 2001, and the sample has been followed up every 2 years, until 2018, 4 waves follow-ups were conducted.

**Reference**

1. Sonnega A, Faul JD, Ofstedal MB, Langa KM, Phillips JW, Weir DR. Cohort Profile: the Health and Retirement Study (HRS). Int J Epidemiol. 2014;43:576-585. doi:10.1093/ije/dyu067

2. Steptoe A, Breeze E, Banks J, Nazroo J. Cohort profile: the English longitudinal study of ageing. Int J Epidemiol. 2013;42:1640-1648. doi:10.1093/ije/dys168

3. Börsch-Supan A, Brandt M, Hunkler C, et al. Data Resource Profile: the Survey of Health, Ageing and Retirement in Europe (SHARE). Int J Epidemiol. 2013;42:992-1001. doi:10.1093/ije/dyt088

4. Lee Y, Yeung WJ. Cohort matters: The relationships between living arrangements and psychological health from the Korean Longitudinal Study of Aging (KLoSA). J Affect Disord. 2022;299:652-657. doi:10.1016/j.jad.2021.10.103

5. Wong R, Michaels-Obregon A, Palloni A. Cohort Profile: The Mexican Health and Aging Study (MHAS). Int J Epidemiol. 2017;46(2):e2. doi:10.1093/ije/dyu263

6. Zhao Y, Hu Y, Smith JP, Strauss J, Yang G. Cohort profile: the China Health and Retirement Longitudinal Study (CHARLS). Int J Epidemiol. 2014;43:61-68. doi:10.1093/ije/dys20
