# Supplementary material for: Country- and individual-level factors associated with healthy working life expectancy: A multinational longitudinal study
Source: PLoS Med. 2026 Sep 10;23(9):e1004676. doi: 10.1371/journal.pmed.1004676 (PMC13561363; doi:10.1371/journal.pmed.1004676)
Supplement: Supplementary file 4 [file pmed.1004676.s004.docx]

**S2 Text**. **Methods to gain mortality information: Exit and Post-exit Interviews**

1. HRS: If a respondent passes away, the study tries to do a follow-up interview called an "exit interview" with someone who knew them well, like their spouse, child, or another person close to them. This interview aims to gather information about health care costs, family matters, and what was happening in the person's life near the end, as well as how their belongings and money were handled after they died. If there are still questions left after the exit interview, especially about the settling of the person's estate, a "post-exit interview" might be done in the next survey wave. The post-exit interview is triggered when the exit proxy respondent reports that a deceased respondent's estate, will, or trust has not been settled.

**Source: Sonnega, A. (2025). Using Health and Retirement Study data: A guide for new users. Survey Research Center, Institute for Social Research, University of Michigan, Ann Arbor, MI.**

2. SHARE: SHARE requests interviewers to confirm the decease of a respondent by a proxy-respondent. In case of decease, interviewers try to conduct an end-of-life interview, which mainly contains information on the circumstances of death like time and cause of death. The proxy-respondent can be a family member, a household member, a neighbor or any other person of the closer social network of the deceased respondent.

**Source: Bergmann, M. Kneip, T., De Luca, G., & Scherpenzeel, A. (2019). Survey participation in the Survey of Health, Ageing and Retirement in Europe (SHARE), Wave 1-7. Based on Release 7.0.0. SHARE Working Paper Series 41-2019. Munich: SHARE-ERIC.**

3. ELSA: An End-of-Life module was introduced at wave 2 to collect information about a respondent who has died since their last interview. A close friend or relative is interviewed and asked the respondent’s health, social, and economic circumstances in the last 2 years of their life, as well as their assets after they died. Details about the date, place, and circumstances of death are also collected.

**Source: Zaninotto, P., Steptoe, A. (2019). English Longitudinal Study of Ageing. In: Gu, D., Dupre, M. (eds) Encyclopedia of Gerontology and Population Aging. Springer, Cham.**

4. KLoSA: Starting with Wave 2, The KLoSA exit survey including death date, age, cause of death et.al, was conducted as an interview with surrogate respondents of the decedents, such as family, relatives, or neighbors.

**Source: Jenny Wilkens, Hae Yeun Park, Drystan Phillips, Youngha Oh, & Jinkook Lee. Harmonized KLoSA Documentation VERSION D.2 (2006-2018). Gateway to Global Aging Data, 2025.**

5. MHAS: Year and month of death are based on questions asked as part of the Next-of-Kin interview asked at each wave starting with Wave2. The Next-of-Kin interview is conducted in the case the subject is reported no longer alive.

**Source: Michaels-Obregon, Alejandra, Jenny Wilkens, Drystan Phillips, Rebeca Wong, & Jinkook Lee. Gateway Harmonized MHAS, Version D.** **Gateway to Global Aging Data, 2025.**

6. CHARLS: Like HRS, for persons who have died, an exit module was conducted. In the exit module, information on various conditions just prior to death and information on the cause of death since wave 2.

**Source: Zhao, Yaohui, John Strauss, Xinxin Chen, Yafeng Wang, Jinquan Gong, Qinqin Meng, Gewei Wang, Huali Wang. (2020). China Health and Retirement Longitudinal Study Wave 4 User’s Guide, National School of Development, Peking University.**
