# Supplementary material for: Country- and individual-level factors associated with healthy working life expectancy: A multinational longitudinal study
Source: PLoS Med. 2026 Sep 10;23(9):e1004676. doi: 10.1371/journal.pmed.1004676 (PMC13561363; doi:10.1371/journal.pmed.1004676)
Supplement: Supplementary file 5 [file pmed.1004676.s005.docx]

**S3 Text. Measures of** **individual-level and country-level factors of HWLE**

| **Variable** | **Definition/Coding** | **Reference period / Assessment year** |
| --- | --- | --- |
| **Individual-level factors** |  |  |
| Gender | Self-reported as men or women | This variable was self-reported when the participant was first enrolled in the cohort. |
| Marital status | Self-reported as married or unmarried | This variable was self-reported when the participant was first enrolled in the cohort. |
| Education level | Harmonized according to simplified version of 1997 International Standard Classification of Education:  - Low: less than upper secondary  - Intermediate: upper secondary or vocational training  - High: tertiary education | This variable was self-reported when the participant was first enrolled in the cohort. |
| Physical activity | Self-reported as yes or no | This variable was self-reported when the participant was first enrolled in the cohort. |
| Drinking | Self-reported as yes or no | This variable was self-reported when the participant was first enrolled in the cohort. |
| Smoking | Self-reported as never, former, or current smoker | This variable was self-reported when the participant was first enrolled in the cohort. |
| Diseases | Self-reported presence of doctor-diagnosed diseases (hypertension, diabetes, cancer, lung disease, heart problems, stroke, or arthritis) | This variable was self-reported when the participant was first enrolled in the cohort. |
| **Country-level factors** |  |  |
| GDP per capita (constant 2015 US$) | GDP per capita is gross domestic product divided by midyear population, which is in constant 2015 U.S. dollars^1^. | The country-level indicator in the year when the individual was first enrolled in the cohort; if data for that year were unavailable, the value from the preceding year was used. |
| The Gini index | the Gini index measures the extent to which the distribution of income or consumption among individuals or households within an economy deviates from a perfectly equal distribution. A Gini index of 0 represents perfect equality, while an index of 100 implies perfect inequality^2^. | The country-level indicator in the year when the individual was first enrolled in the cohort; if data for that year were unavailable, the value from the preceding year was used. |
| Current health expenditure (% of GDP) | Level of current health expenditure including consumed healthcare goods and services expressed as a percentage of GDP^3^. | The country-level indicator in the year when the individual was first enrolled in the cohort; if data for that year were unavailable, the value from the preceding year was used. |
| Tax revenue (% of GDP) | Tax revenue refers to compulsory transfers to the central government for public purposes^4^. | The country-level indicator in the year when the individual was first enrolled in the cohort; if data for that year were unavailable, the value from the preceding year was used. |
| Ease of doing business | The ease of doing business scores benchmark economies with respect to regulatory best practice, showing the proximity to the best regulatory performance on doing business indicators. It ranges from 0 to 100, where 0 represents the worst regulatory performance and 100 the best regulatory performance^5^. | The rankings for all economies are benchmarked to May 2019. |
| Government effectiveness | Government effectiveness captures perceptions of the quality of public services, the quality of the civil service and the degree of its independence from political pressures, the quality of policy formulation and implementation, and the credibility of the government's commitment to such policies^6^. | The country-level indicator in the year when the individual was first enrolled in the cohort; if data for that year were unavailable, the value from the preceding year was used. |
| Control of corruption | Control of corruption captures perceptions of the extent to which public power is exercised for private gain, including both petty and grand forms of corruption, as well as "capture" of the state by elites and private interests^7^. | The country-level indicator in the year when the individual was first enrolled in the cohort; if data for that year were unavailable, the value from the preceding year was used. |
| Political stability and absence of violence/terrorism (PSAVT) | Political Stability and Absence of Violence/Terrorism measures perceptions of the likelihood of political instability and/or politically-motivated violence, including terrorism^8^. In this study, higher values indicate greater political stability and a lower likelihood of political instability and politically motivated violence. | The country-level indicator in the year when the individual was first enrolled in the cohort; if data for that year were unavailable, the value from the preceding year was used. |
| Flexible Working Arrangements | Flexible working arrangements, 1-7 (best). Response to the survey question: In your country, to what extent do companies offer flexible working arrangements such as remote and part-time work?” [1 = Not at all; 7 = To a great extent] ^9^ | 2022–2023 weighted average / 2024; Source: World Economic Forum Executive Opinion Survey, accessed through World Bank Data360. |
| Labour Force Participation (% of adult population) | Proportion of the population aged 15 and older that is economically active. Labour force participation rate is the proportion of the population aged 15 and older that is economically active: that is, all people who supply labour for the production of goods and services during a specified period^10^. | 2022 / 2024; Source: World Bank World Development Indicators, accessed through World Bank Data360. |
| Equal Workforce Opportunities | In your country, to what extent do companies give equal workforce opportunities to [the following groups]? Women; those from a typically disadvantaged religious, ethnic or racial background; those with disabilities; those who identify as LGBTI. [1 = Not at all; 7 = To a great extent]. The final score was calculated as the average of the four items^11^. | 2022–2023 weighted average / 2024; Source: World Economic Forum Executive Opinion Survey, accessed through World Bank Data360. |
| Hiring and Firing Practices | Response to the survey question: “In your country, to what extent do regulations allow flexible hiring and firing of workers?” [1 = Not at all; 7 = To a great extent] ^12^ | 2022–2023 weighted average / 2024; Source: World Economic Forum Executive Opinion Survey, accessed through World Bank Data360. |

**References**

1. World Bank. GDP per capita (constant 2015 US$): World Bank; 2024 [Available from: https://data.worldbank.org/indicator/NY.GDP.PCAP.KD accessed 10/13 2024.

2. World Bank. Gini index (World Bank estimate): World Bank; 2024 [Available from: https://genderdata.worldbank.org/en/indicator/si-pov-gini accessed 10/13 2024.

3. World Bank. Current health expenditure (% of GDP): World Bank; [Available from: https://data.worldbank.org/indicator/SH.XPD.CHEX.GD.ZS accessed 10/13 2024.

4. World Bank. Tax revenue (% of GDP) [Available from: https://data.worldbank.org/indicator/GC.TAX.TOTL.GD.ZS accessed 10/14 2024.

5. World Bank. Ease of doing business score (0 = lowest performance to 100 = best performance) [Available from: https://data.worldbank.org/indicator/IC.BUS.DFRN.XQ?end=2 accessed 10/14 2024.

6. World Bank. Government Effectiveness: Estimate 2024 [Available from: https://data.worldbank.org/indicator/GE.EST accessed 10/14 2024.

7. World Bank. Control of Corruption: Estimate 2024 [Available from: https://data.worldbank.org/indicator/CC.EST accessed 10/14 2024.

8. World Bank. Political Stability and Absence of Violence/Terrorism: Estimate: World Bank; [Available from: https://data.worldbank.org/indicator/PV.EST?view=chart accessed 10/14 2024.

9. World Bank. Flexible working arrangements, 1-7 (best) [Available from: https://data360.worldbank.org/en/indicator/WEF_TTDI_EOSQ515 accessed 7/18 2026

10. World Bank. Labour force participation, % of adult pop. [Available from: https://data360.worldbank.org/en/indicator/WEF_TTDI_LABOURPART accessed 7/18 2026

11. World Bank. Equal workforce opportunities, 1-7 (best). [Available from: https://data360.worldbank.org/en/indicator/WEF_TTDI_EQUALWORKFORCE accessed 7/18 2026

12. World Bank. Hiring and firing practices, 1-7 (best) [Available from: https://data360.worldbank.org/en/indicator/WEF_GCIHH_EOSQ134 accessed 7/18 202
