# Supplementary material for: Country- and individual-level factors associated with healthy working life expectancy: A multinational longitudinal study
Source: PLoS Med. 2026 Sep 10;23(9):e1004676. doi: 10.1371/journal.pmed.1004676 (PMC13561363; doi:10.1371/journal.pmed.1004676)
Supplement: Supplementary file 6 [file pmed.1004676.s006.docx]

**S4 Text. Methods of HWLE calculation**

Discrete-time Markov multistate model followed two steps to estimate HWLE with standard errors. Firstly, multinomial logistic regression was used to model the age-specific probabilities of transition from and to each state over small discrete time intervals (interpolation steps) based on the transitions observed in the data. Maximum likelihood estimates of transition probability model parameters were found by evaluating the product of the transition probabilities for each step contained within each observed transition or sequence of transitions (where more than one transition was observed for an individual). Secondly, HWLE was estimated according to the health and work state occupied at age 50 years and averaged (weighted by the observed prevalence of occupying each health and work state at age 50 years) to estimate healthy working life expectancy for the population.

Moreover, health expectancies were also estimated for individuals categorized as HnW, nHW and nHnW. The estimate of life expectancy was derived by adding up all four health expectancies. Health expectancies and life expectancy were estimated for overall participants then by each individual-level and country-level factors. Considering the facts that participants of the cohorts were followed up at about two-year intervals, all estimations used biennial transition probability models with interpolation steps of size 24 months.
