# Supplementary material for: Country- and individual-level factors associated with healthy working life expectancy: A multinational longitudinal study
Source: PLoS Med. 2026 Sep 10;23(9):e1004676. doi: 10.1371/journal.pmed.1004676 (PMC13561363; doi:10.1371/journal.pmed.1004676)
Supplement: Supplementary file 7 [file pmed.1004676.s007.docx]

**S1 appendix**

**Fig A.** Flowchart of participants

**Fig B.** Hypothetical transitions of healthy and working status

**Fig C.** Life expectancy in different states at age 50 by starting state

**Fig D.** Results of sensitivity analyses

**Table A.** Number of individuals in each wave across six databases

**Table B.** Sample characteristics across six databases

**Table C.** Sample characteristics of SHARE participants by country

**Table D.** Life expectancy in different states at age 50 by starting state

**Table E.** Life expectancy in different states across cohorts

**Table F.** Life expectancy in different states across countries

**Table G.** Results of sensitivity analyses by including all missing data, sampling randomly, using different interpolations and health definitions

**154 were excluded due to missing age or birth-date**

ELSA:18

CHARLS: 136

**54,101 were excluded due to being interviewed only in one wave**

HRS: 1,870

ELSA: 4,836

SHARE: 40,075

KLoSA: 584

CHARLS: 1,815

MHAS: 5, 505

**11,465** were excluded due to missing individual-level factors

HRS: 1,181

ELSA:859

SHARE: 1,052

KLoSA: 2

CHARLS:2,690

MHAS:5,681

HRS(N:42,233): 4,024 aged <50 years were excluded

ELSA(N:19,802): 967 aged <50 years were excluded

SHARE(N:139,620): 4,918 aged <50 years were excluded

KLoSA(N:11,174): 1,789 aged <50 years were excluded

CHARLS (N:25,586): 8,373 aged <50 years were excluded

MHAS(26,839): 3,561 aged<50 years were excluded

**Total 241,622**

**175,318 were included**

HRS: 35,158

ELSA: 13,122

SHARE: 93,575

KLoSA: 8,799

CHARLS: 12,572

MHAS: 12,092

**Fig A. Flowchart of participants.** HRS: the Health and Retirement Survey. ELSA: the English Longitudinal Study of Ageing. SHARE: the Survey of Health, Ageing and Retirement in Europe. KLoSA: the Korean Longitudinal Study of Aging. CHARLS: the China Health and Retirement Longitudinal Study. MHAS: the Mexican Health and Aging Study.

Dead

Not healthy and working

Not healthy and not working

Healthy and working

Healthy and not working

**Fig B. Hypothetical transitions of healthy and working status**

**Fig C. Life expectancy in different states at age 50 by starting state.** HWLE: healthy and working life expectancy; HnWLE: life expectancy on healthy and not working status; nHWLE: life expectancy on not healthy and working status; nHnWLE: life expectancy on not healthy and not working status.

**Fig D. Results of sensitivity analyses.** Method 1: primary analysis repeated using a 12-month interpolation interval. Method 2: primary analysis repeated using an 18-month interpolation interval. Method 3: primary analysis repeated using a 36-month interpolation interval. Method 4: primary analysis repeated in the intact sample (n=186,783), including the 11,465 individuals excluded from the main analysis because of missing individual-level covariates. Method 5: primary analysis repeated in a randomly selected 70% sub-sample of the analytic sample (n=122,722). Method 6: primary analysis repeated using ADL as an alternative health definition and a 24-month interpolation interval. Method 7: primary analysis repeated using ADL as an alternative health definition and a 12-month interpolation interval. HWLE: healthy and working life expectancy; HnWLE: life expectancy on healthy and not working status; nHWLE: life expectancy on not healthy and working status; nHnWLE: life expectancy on not healthy and not working status. SRH: self-reported health; ADL: activities of daily living.

**Table A. Number of individuals in each wave across six databases**

|  | **Overall** | **Wave1** | **Wave2** | **Wave3** | **Wave4** | **Wave5** | **Wave6** | **Wave7** | **Wave8** | **Wave9** | **Wave10** | **Wave11** | **Wave12** | **Wave13** | **Wave14** |
| --- | --- | --- | --- | --- | --- | --- | --- | --- | --- | --- | --- | --- | --- | --- | --- |
| **SHARE** | 93,575 | 48,843 | 53,029 | 50,508 | 55,844 | 44,896 | 32,942 | 18,450 | — | — | — | — | — | — | — |
| **CHARLS** | 12,572 | 11,045 | 11,415 | 11,429 | 11,125 | — | — | — | — | — | — | — | — | — | — |
| **ELSA** | 13,122 | 10,238 | 8,841 | 9,421 | 8,986 | 8,270 | — | — | — | — | — | — | — | — | — |
| **MHAS** | 12,092 | 7,214 | 6,715 | 10,865 | 11,194 | 9,092 | — | — | — | — | — | — | — | — | — |
| **HRS** | 35,158 | 10,989 | 17,235 | 16,469 | 19,371 | 17,720 | 16,354 | 17,672 | 16,230 | 15,028 | 18,771 | 17,648 | 15,995 | 17,145 | 14,445 |
| **KLoSA** | 8,799 | 7,912 | 7,226 | 6,592 | 6,213 | 6,687 | 6,308 | 3,625 | — | — | — | — | — | — | — |

**Table B. Sample characteristics across six databases**

|  | **Overall**  **(n=175,318)** | **SHARE**  **(n=93,575)** | **CHARLS**  **(n=12,572)** | | **ELSA**  **(n=13,122)** | | **MHAS**  **(n=12,092)** | | **HRS**  **(n=35,158)** | | **KLoSA**  **(n=8,799)** |
| --- | --- | --- | --- | --- | --- | --- | --- | --- | --- | --- | --- |
| **Age at baseline, years (SD)** | 63.91(9.68) | 64.24(9.80) | 61.53(8.04) | | 64.04(9.96) | | 68.16(6.57) | | 60.66(9.99) | | 63.79(10.02) |
| **Gender** |  |  |  | |  | |  | |  | |  |
| Men | 79,833(45.5%) | 41,999(44.9%) | 6,220(49.5%) | | 6,049(46.1%) | | 5,415(44.8%) | | 16,283(46.3%) | | 3,867(43.9%) |
| Women | 95,485(54.5%) | 51,576(55.1%) | 6,352(50.5%) | | 7,073(53.9%) | | 6,677(55.2%) | | 18,875(53.7%) | | 4,932(56.0%) |
| **Education level** |  |  |  | |  | |  | |  | |  |
| Low | 65,789(37.5%) | 22,853(24.4%) | 11,217(89.2%) | | 4,950(37.7%) | | 10,335(85.5%) | | 10,732(30.5%) | | 5,702(64.8%) |
| Intermediate | 66,353(37.9%) | 47,279(50.5%) | 1,145(9.1%) | | 5,307(40.5%) | | 403(3.3%) | | 9,951(28.3%) | | 2,268(25.8%) |
| High | 43,176(24.6%) | 23,443(25.1%) | 210(1.7%) | | 2,865(21.8%) | | 1,354(11.2%) | | 14,475(41.2%) | | 829(9.4%) |
| **Marital status** |  |  |  | |  | |  | |  | |  |
| Married | 130,872(74.7%) | 70,435(75.3%) | 10,848(86.3%) | | 9,313(71.0%) | | 9,231(76.3%) | | 24,308(69.1%) | | 6,737(76.6%) |
| Other | 44,446(25.3%) | 23,140(24.7%) | 1,724(13.7%) | | 3,809(29.0%) | | 2,861(23.7%) | | 10,850(30.9%) | | 2,062(23.4%) |
| **Physical activity** |  |  | |  | |  | |  | |  | |
| Yes | 72,285(41.2%) | 46,295(49.5%) | 3,813(30.3%) | | 3,832(29.2%) | | 5,063(41.9%) | | 10,028(28.5%) | | 3,254(37.0%) |
| No | 103,033(58.8%) | 47,280 (50.5%) | 8,759(69.7%) | | 9,290(70.8%) | | 7,029(58.1%) | | 25,130(71.5%) | | 5,545(63.0%) |
| **Drinking** |  |  |  | |  | |  | |  | |  |
| Yes | 88,140(50.3%) | 44,332(47.4%) | 7,442(59.2%) | | 1,480(11.3%) | | 8,213(67.9%) | | 21,141(60.1%) | | 5,532(62.9%) |
| No | 87,178(49.7%) | 49,243(52.6%) | 5,130(40.8%) | | 11,642(88.7%) | | 3,879(32.1%) | | 14,017(39.9%) | | 3,267(37.1%) |
| **Smoking** |  |  |  | |  | |  | |  | |  |
| Never | 92,022(52.5%) | 51,596(55.2%) | 7,384(58.7%) | | 4,880(37.2%) | | 7,167(59.3%) | | 14,688 (41.8%) | | 6,307(71.7%) |
| Former | 48,191(27.5%) | 24,357(26.0%) | 1,225(9.8%) | | 5,954(45.4%) | | 2,943(24.3%) | | 12,814(36.4%) | | 898(10.2%) |
| Current | 35,105(20.0%) | 17,622(18.8%) | 3,963(31.5%) | | 2,288(17.4%) | | 1,982(16.4%) | | 7,656(21.8%) | | 1,594(18.1%) |
| **Follow-up time, years (SD)** | 8.22(5.58) | 6.17(4.17) | 6.26(1.50) | | 6.24(3.69) | | 9.31(6.54) | | 11.26(8.14) | | 8.33(3.98) |

**Table C. Sample characteristics of SHARE participants by country**

| **Country** | **N** | **Age at baseline, years (SD)** | **Follow-up time, years (SD)** | **Gender** | |
| --- | --- | --- | --- | --- | --- |
|  |  |  |  | Men | Women |
| Austria | 4,602 | 64.14(9.11) | 6.52(3.70) | 1,932(41.98) | 2,670(58.02) |
| Germany | 5,519 | 62.90(9.26) | 6.40(3.91) | 2,630(47.65) | 2,889(52.35) |
| Sweden | 5,263 | 65.43(9.68) | 7.12(4.86) | 2,480(47.12) | 2,783(52.88) |
| Netherlands | 4,134 | 62.96(9.56) | 7.18(4.61) | 1,932(46.73) | 2,202(53.27) |
| Spain | 6,629 | 65.42(10.35) | 6.58(4.23) | 3,026(45.65) | 3,603(54.35) |
| Italy | 5,854 | 63.27(9.19) | 7.17(4.58) | 2,724(46.53) | 3,130(53.47) |
| France | 5,824 | 63.53(10.37) | 7.14(4.42) | 2,587(44.42) | 3,237(55.58) |
| Denmark | 4,560 | 62.29(10.03) | 7.06(4.60) | 2,144(47.02) | 2,416(52.98) |
| Greece | 4,915 | 63.94(10.29) | 7.69(5.28) | 2,245(45.68) | 2,670(54.32) |
| Switzerland | 3,329 | 63.39(9.50) | 7.78(3.88) | 1,561(46.89) | 1,768(53.11) |
| Belgium | 6,854 | 62.22(9.84) | 6.60(4.24) | 3,181(46.41) | 3,673(53.59) |
| Israel | 2,913 | 64.61(9.89) | 7.24(4.35) | 1,339(45.97) | 1,574(54.03) |
| Czech Republic | 6,061 | 64.83(9.21) | 6.24(3.54) | 2,590(42.73) | 3,471(57.27) |
| Poland | 3,484 | 63.30(9.57) | 5.83(4.34) | 1,590(45.64) | 1,894(54.36) |
| Luxembourg | 1,560 | 63.93(9.37) | 4.26(1.97) | 723(46.35) | 837(53.65) |
| Portugal | 1,582 | 64.87(9.48) | 3.48(1.16) | 695(43.93) | 887(56.07) |
| Slovenia | 3,905 | 65.61(9.86) | 5.02(2.65) | 1,694(43.38) | 2,211(56.62) |
| Estonia | 6,713 | 66.05(10.06) | 5.48(3.00) | 2,753(41.01) | 3,960(58.99) |
| Croatia | 1,267 | 66.39(9.62) | 3.52(1.70) | 579(45.70) | 688(54.30) |
| Lithuania | 1,326 | 66.34(10.22) | 2.53(0.14) | 470(35.44) | 856(64.56) |
| Bulgaria | 854 | 66.26(9.00) | 2.65(0.11) | 343(40.16) | 511(59.84) |
| Latvia | 726 | 66.38(9.61) | 2.42(0.19) | 262(36.09) | 464(63.91) |
| Slovakia | 929 | 61.63(7.89) | 2.43(0.11) | 429(46.18) | 500(53.82) |

**Table D. Life expectancy in different states at age 50 by starting state**

| **Starting state** | **TLE** | **HWLE** | **HnWLE** | **nHWLE** | **nHnWLE** |
| --- | --- | --- | --- | --- | --- |
| **HW** | 33.73(33.63-33.83) | 12.19(12.11-12.27) | 11.74(11.66-11.82) | 2.04(2.01-2.07) | 7.76(7.70-7.82) |
| **HnW** | 33.56(33.47-33.66) | 7.54(7.42-7.66) | 15.78(15.65-15.91) | 1.57(1.54-1.60) | 8.67(8.60-8.74) |
| **nHW** | 33.17(33.05-33.30) | 8.79(8.68-8.90) | 11.53(11.44-11.62) | 4.41(4.34-4.48) | 8.44(8.36-8.52) |
| **nHnW** | 32.49(32.37-32.62) | 5.53(5.43-5.63) | 13.01(12.91-13.11) | 1.90(1.84-1.96) | 12.05(11.93-12.17) |

Rows represent the starting state at age 50, and columns represent life expectancy in each specific state. TLE: total life expectancy; HWLE: healthy and working life expectancy; HnWLE: life expectancy on healthy and not working status; nHWLE: life expectancy on not healthy and working status; nHnWLE: life expectancy on not healthy and not working status.

**Table E. Life expectancy in different states across cohorts**

| **Cohort** | **N** | **TLE** | **HWLE** | **HnWLE** | **nHWLE** | **nHnWLE** |
| --- | --- | --- | --- | --- | --- | --- |
| SHARE | 94,627 | 34.40(34.24-34.55) | 10.71(10.55-10.87) | 10.97(10.84-11.10) | 2.80(2.70-2.90) | 9.92(9.80-10.04) |
| CHARLS | 15,262 | 30.85(30.44-31.27) | 12.76(12.49-13.03) | 7.87(7.62-8.12) | 4.15(3.98-4.32) | 6.08(5.85-6.30) |
| KLoSA | 8,801 | 33.15(32.73-33.57) | 13.46(13.03-13.90) | 11.51(11.08-11.93) | 1.55(1.43-1.67) | 6.63(6.39-6.86) |
| HRS | 36,339 | 30.42(30.24-30.62) | 10.69(10.50-10.88) | 11.16(11.00-11.31) | 1.84(1.77-1.91) | 6.74(6.62-6.85) |
| ELSA | 13,981 | 30.78(30.41-31.14) | 9.94(9.54-10.34) | 13.23(12.87-13.59) | 1.40(1.23-1.57) | 6.20(5.96-6.45) |
| MHAS | 17,773 | 30.58(30.30-30.86) | 11.05(10.73-11.38) | 15.03(14.71-15.36) | 1.05(0.97-1.13) | 3.44(3.31-3.57) |

TLE: total life expectancy; HWLE: healthy and working life expectancy; HnWLE: life expectancy on healthy and not working status; nHWLE: life expectancy on not healthy and working status; nHnWLE: life expectancy on not healthy and not working status

**Table F.** **Life expectancy in different states across countries**

| **Country** | **N** | **TLE** | **HWLE** | **HnWLE** | **nHWLE** | **nHnWLE** |
| --- | --- | --- | --- | --- | --- | --- |
| Austria | 4,631 | 38.31(37.42-39.21) | 8.61(7.83-9.39) | 15.60(14.91-16.29) | 1.86(1.47-2.25) | 12.25(11.56-12.94) |
| Germany | 5,534 | 36.58(35.80-37.36) | 10.60(9.96-11.23) | 10.26(9.77-10.76) | 4.30(3.80-4.80) | 11.42(10.81-12.04) |
| Sweden | 5,296 | 34.43(33.85-35.01) | 14.57(13.90-15.23) | 9.83(9.42-10.25) | 3.10(2.60-3.59) | 6.93(6.56-7.30) |
| Netherlands | 4,173 | 34.00(33.24-34.76) | 12.83(12.05-13.60) | 11.61(10.93-12.28) | 2.74(2.17-3.31) | 6.83(6.36-7.30) |
| Spain | 6,697 | 36.47(35.89-37.06) | 9.66(9.16-10.17) | 11.72(11.30-12.15) | 2.32(1.96-2.68) | 12.77(12.32-13.22) |
| Italy | 5,881 | 35.92(35.37-36.48) | 9.06(8.46-9.66) | 11.19(10.71-11.68) | 2.24(1.91-2.57) | 13.44(12.93-13.94) |
| France | 5,861 | 36.21(35.54-36.88) | 9.49(8.93-10.06) | 13.17(12.67-13.67) | 2.23(1.87-2.59) | 11.31(10.83-11.79) |
| Denmark | 4,573 | 33.10(32.50-33.69) | 14.02(13.46-14.58) | 11.11(10.60-11.63) | 2.17(1.90-2.44) | 5.80(5.43-6.17) |
| Greece | 4,921 | 34.82(34.21-35.42) | 12.47(11.70-13.24) | 11.85(11.14-12.57) | 1.71(1.28-2.13) | 8.79(8.29-9.29) |
| Switzerland | 3,348 | 40.46(39.51-41.41) | 15.12(14.59-15.65) | 16.61(15.84-17.38) | 1.80(1.45-2.15) | 6.93(6.35-7.52) |
| Belgium | 6,877 | 35.62(35.07-36.17) | 9.22(8.57-9.88) | 15.44(14.90-15.99) | 1.96(1.60-2.32) | 9.00(8.58-9.42) |
| Israel | 3,013 | 32.66(31.90-33.42) | 12.26(11.25-13.26) | 7.85(7.21-8.49) | 3.62(2.97-4.27) | 8.93(8.34-9.52) |
| Czech Republic | 6,095 | 32.06(31.49-32.62) | 9.96(9.41-10.51) | 11.19(10.74-11.63) | 2.52(2.20-2.85) | 8.39(8.03-8.76) |
| Poland | 3,532 | 30.83(29.96-31.70) | 8.27(7.33-9.20) | 7.30(6.76-7.84) | 3.13(2.47-3.79) | 12.13(11.42-12.84) |
| Luxembourg | 1,560 | 36.19(34.45-37.93) | 7.52(5.81-9.23) | 14.55(13.22-15.87) | 2.36(0.83-3.88) | 11.77(10.40-13.14) |
| Portugal | 1,582 | 25.53(23.71-27.34) | 3.97(1.83-6.12) | 3.96(3.04-4.88) | 7.11(4.73-9.48) | 10.49(8.89-12.09) |
| Slovenia | 3,912 | 33.48(32.50-34.46) | 8.38(7.31-9.44) | 13.09(12.24-13.94) | 2.00(1.32-2.69) | 10.02(9.38-10.65) |
| Estonia | 6,727 | 30.08(29.40-30.76) | 8.82(8.12-9.52) | 2.29(2.09-2.49) | 8.66(8.09-9.22) | 10.31(9.80-10.82) |
| Croatia | 1,267 | 27.21(25.41-29.02) | 10.99(9.06-12.91) | 7.58(5.98-9.19) | 1.83(0.57-3.08) | 6.82(5.46-8.17) |
| Lithuania | 1,431 | 33.10(30.12-36.09) | 9.81(7.82-11.81) | 6.16(4.89-7.43) | 4.53(3.29-5.77) | 12.60(10.33-14.86) |
| Bulgaria | 948 | 28.71(25.30-32.12) | 9.53(6.68-12.37) | 9.63(7.56-11.71) | 1.64(0.64-2.64) | 7.91(5.94-9.88) |
| Latvia | 774 | 27.89(18.81-36.97) | 5.48(2.48-8.48) | 2.21(1.22-3.20) | 4.05(2.33-5.76) | 16.15(10.41-21.90) |
| Slovakia | 979 | 31.10(27.73-34.47) | 10.87(10.05-11.68) | 11.94(10.19-13.69) | 0.45(0.07-0.83) | 7.84(5.20-10.49) |
| China | 15,262 | 30.85(30.44-31.27) | 12.76(12.49-13.03) | 7.87(7.62-8.12) | 4.15(3.98-4.32) | 6.08(5.85-6.30) |
| South Korea | 8,801 | 33.15(32.73-33.57) | 13.46(13.03-13.90) | 11.51(11.08-11.93) | 1.55(1.43-1.67) | 6.63(6.39-6.86) |
| USA | 36,339 | 30.42(30.24-30.62) | 10.69(10.50-10.88) | 11.16(11.00-11.31) | 1.84(1.77-1.91) | 6.74(6.62-6.85) |
| UK | 13,981 | 30.78(30.41-31.14) | 9.94(9.54-10.34) | 13.23(12.87-13.59) | 1.40(1.23-1.57) | 6.20(5.96-6.45) |
| Mexico | 17,773 | 30.58(30.30-30.86) | 11.05(10.73-11.38) | 15.03(14.71-15.36) | 1.05(0.97-1.13) | 3.44(3.31-3.57) |

TLE: total life expectancy; HWLE: healthy and working life expectancy; HnWLE: life expectancy on healthy and not working status; nHWLE: life expectancy on not healthy and working status; nHnWLE: life expectancy on not healthy and not working status.

**Table G. Results of sensitivity analyses by including all missing data，sampling randomly，using different interpolations and health definitions**

| **Method** | **Step** | **Sample Size** | **Total life expectancy, years (95% CI)** | **Life expectancy at age 50 in different work and health states, years (95% CI)** | | | |
| --- | --- | --- | --- | --- | --- | --- | --- |
|  |  |  |  | **Healthy and working** | **Healthy and not working** | **Not healthy and working** | **Not healthy and not working** |
| **SRH** | | | | | | | |
| Method 1 | Step=12 | 175,318 | 33.51(33.40-33.62) | 10.95(10.86-11.05) | 12.16(12.07-12.26) | 2.27(2.23-2.32) | 8.12(8.05-8.19) |
| Method 2 | Step=18 | 175,318 | 33.56(33.46-33.66) | 10.98(10.88-11.08) | 12.18(12.09-12.27) | 2.26(2.23-2.32) | 8.13(8.06-8.20) |
| Method 3 | Step=36 | 175,318 | 33.91(33.81-34.00) | 11.09(10.97-11.20) | 12.23(12.13-12.32) | 2.38(2.33-2.44) | 8.21(8.14-8.28) |
| Method 4 | Step=24 (Intact sample) | 186,783 | 32.19(32.10-32.29) | 10.81(10.72-10.91) | 11.56(11.47-11.65) | 2.25(2.20-2.30) | 7.57(7.50-7.63) |
| Method 5 | Step=24 (Random Sampling 70%) | 122,722 | 33.58(33.46-33.69) | 11.02(10.89-11.14) | 12.18(12.07-12.29) | 2.27(2.22-2.33) | 8.11(8.03-9.19) |
| **ADL** | | | | | | | |
| Method 6 | Step=24 | 186,879 | 32.28(32.17-32.38) | 11.72(11.63-11.81) | 12.43(12.32-12.54) | 1.46(1.43-1.49) | 6.67(6.61-6.73) |
| Method 7 | Step=12 | 186,879 | 32.22(32.12-32.31) | 11.71(11.62-11.79) | 12.36(12.26-12.45) | 1.47(1.44-1.49) | 6.68(6.63-6.74) |

Method 1: primary analysis repeated using a 12-month interpolation interval. Method 2: primary analysis repeated using an 18-month interpolation interval. Method 3: primary analysis repeated using a 36-month interpolation interval. Method 4: primary analysis repeated in the intact sample (n = 186,783), including the 11,465 individuals excluded from the main analysis because of missing individual-level covariates. Method 5: primary analysis repeated in a randomly selected 70% sub-sample of the analytic sample (n = 122,722). Method 6: primary analysis repeated using ADL as an alternative health definition and a 24-month interpolation interval. Method 7: primary analysis repeated using ADL as an alternative health definition and a 12-month interpolation interval. SRH: self-reported health; ADL: activities of daily living.
