# Supplementary material for: Country- and individual-level factors associated with healthy working life expectancy: A multinational longitudinal study
Source: PLoS Med. 2026 Sep 10;23(9):e1004676. doi: 10.1371/journal.pmed.1004676 (PMC13561363; doi:10.1371/journal.pmed.1004676)
Supplement: Supplementary file 9 [file pmed.1004676.s011.docx]

Responses to Reviewers’ Comments for Manuscript PMEDICINE-D-25-02153R1

**Country- and individual-level factors associated with healthy working life expectancy: a multinational longitudinal study**

Addressed Comments for Publication to

PLOS Medicine

by

Jingjie Zhu, Bo Ye, Junling Gao

Dear Editors and Reviewers,

We sincerely appreciate the opportunity to revise our manuscript entitled “Country- and individual-level factors associated with healthy working life expectancy: a multinational longitudinal study”, and we are deeply grateful to the reviewers for their insightful and constructive comments. These comments have been extremely valuable in helping us improve the manuscript and refine our study. We have carefully considered each suggestion and made every effort to address all comments clearly and thoroughly in the revised version. We hope that the revisions have satisfactorily addressed the concerns raised and have improved the quality of the manuscript.

Thank you again for your time, consideration, and the opportunity to revise our work.

**Response to Reviewer #1:**

**Comment 11:**

Unfortunately, I am still struggling to understand which sensitivity analysis results in the supplementary materials (notated by “Method X”) correspond to which description in the Methods. Suggest the authors make this crystal clear by referencing the method number in the text and ensuring each method number 1-7 is covered e.g., “First, to verify whether the results were sensitive to the health definition, health based on activities of daily living was defined as good if there were no difficulties with activities of daily living and poor if difficulty was reported with at least one activity from bathing, dressing, eating, getting in and out of bed, and walking across a room (Method 1) ”.

**Response**:

Thank you for pointing out this important aspect. Following your suggestion, we have added the following description to the main text. “To assess the robustness of the findings, we conducted a series of sensitivity analyses. First, to examine whether the results were sensitive to the interpolation interval used in the transition probability models, analyses were repeated using step sizes of 12 months (Method 1), 18 months (Method 2), and 36 months (Method 3). Second, to assess the potential impact of sample selection, we included the 11,465 individuals who had been excluded from the primary analysis due to missing individual-level covariates and re-estimated the results in the intact sample of 186,783 participants (Method 4). We also randomly selected 70% of the analytic sample and repeated the analyses in this sub-sample of 122,722 participants (Method 5). Third, to assess whether the findings were sensitive to the health definition, we used activities of daily living (ADL) as an alternative indicator of health. Health was defined as good if participants reported no difficulty in bathing, dressing, eating, getting in and out of bed, or walking across a room, and poor if difficulty was reported in at least one of these activities. Based on this alternative definition, analyses were repeated using a 24-month step (Method 6) and a 12-month step (Method 7).” (Page 9, Line 16)

**Comment 12:**

The authors have made good progress on this point but there are still cases where there are definitions used in tables or figures that are not defined in each case e.g., “heart problems” in Figure 3, “GDP” in Figure 4, dataset names in Supplementary Figure 1, and both “SRH” and “ADL” in Supplementary Figure 4. Also in Supplementary Table 5 and Supplementary Figure 4, ensuring enough detail is included in the table/figure notes to succinctly but completely define the analysis method used for each “Method” number. Suggest carefully checking each table and figure and ensuring all definitions are covered.

**Response**:

Thank you for your valuable suggestion. Following your recommendation, we have carefully checked the definitions used in all tables and figures and added the relevant explanations in both the main manuscript and the supplementary information. For example, in **Supplementary fig 1**, we added the following dataset definitions: “HRS, the Health and Retirement Study; ELSA, the English Longitudinal Study of Ageing; SHARE, the Survey of Health, Ageing and Retirement in Europe; KLoSA, the Korean Longitudinal Study of Aging; CHARLS, the China Health and Retirement Longitudinal Study; and MHAS, the Mexican Health and Aging Study.” In **Supplementary fig 4** and **Supplementary table 6**, we also added detailed notes as follows: “Method 1: primary analysis repeated using a 12-month interpolation interval. Method 2: primary analysis repeated using an 18-month interpolation interval. Method 3: primary analysis repeated using a 36-month interpolation interval. Method 4: primary analysis repeated in the intact sample (n=186,783), including the 11,465 individuals excluded from the main analysis because of missing individual-level covariates. Method 5: primary analysis repeated in a randomly selected 70% sub-sample of the analytic sample (n=122,722). Method 6: primary analysis repeated using ADL as an alternative health definition and a 24-month interpolation interval. Method 7: primary analysis repeated using ADL as an alternative health definition and a 12-month interpolation interval”.

**Response to Reviewer #2:**

**Comment 1:**

Abstract - I suggest removing average data but focus on the range. The average is dependent on the selection of the countries.

**Response**:

Thank you for your valuable suggestion. Taking into account all your other suggestions, we focus on to examine individual and national factors associated with HWLE. Following this recommendation, we have revised the relevant text in the Abstract to report the range across countries rather than the average values. “At age 50, HWLE ranged from 3.97 years to 15.12 years across countries.” (Page 2, Line 15-16)

**Comment 2:**

Intro - first paragraph - “It is crucial to extend working life expectancy in healthy state among older workers. ”- please clarify what this means and why it is crucial.

**Response**:

Thank you for your important suggestion. Following your recommendation, we have revised the relevant text in the Introduction section. “In this context, extending working life expectancy in a healthy state among older workers is important, which means not merely prolonging the years individuals spend in the workforce, but ensuring that these additional working years are accompanied by good health and functional capacity. It is crucial because it helps address the labor force shortages associated with population aging while simultaneously reducing the risk of mismatch between evolving retirement policies and individuals’ actual ability to work. By fostering a healthy working life, delayed retirement policies become more sustainable, benefiting both individuals through improved well-being and societies through sustained productivity and reduced healthcare burdens.” (Page 4, Line 8-16)

**Comment 3:**

Intro - Please clarify “Although health status is a major determinant of HWLE for older workers, current evidence suggests that individual-level factors, including gender, education, lifestyle, and chronic conditions—are associated with HWLE.” Health is part of the HWLE construct; how can it also be a determinant?

**Response**:

Thank you for your important comment. We agree that our previous wording was imprecise. We have revised the sentence according to your suggestion. “Previous studies have shown that individual-level factors, including gender, education, lifestyle, and chronic conditions, are associated with HWLE[6, 7, 9, 11]. In addition, country-level factors, such as gross domestic product (GDP) per capita[12], national health expenditures[2], quality of governance[9], and the Gini index[7] are closely related to population health.” (Page 4, Line 1-5)

**Comment 4:**

Intro - Revise “Fully examining the associations between individual- and country-level factors and HWLE can deepen understanding of the relationships between politics, economy, employment, and health, and guide the development of country-specific measures, such as healthcare investment policies, governance capacity building, and the optimization of employment and retirement”. This analysis and manuscript does not do this; please stop overstating what this analysis is doing or explain how your manuscript deepens the understanding between politics, healthcare investment and the optimization of employment and retirement.

**Response**:

Thank you for this important comment. We agree that our previous statement overstated the scope of our analysis. We have revised the sentence to avoid overinterpretation. “While previous research has established the influence of individual-level factors on HWLE, a systematic, multinational evaluation of country-level factors, particularly beyond simple wealth indicators, has been lacking. This study aims to fill this gap by quantifying the distinct associations of the country-level factors with HWLE. Specifically, we move beyond the general understanding that wealthier nations tend to have longer healthy working lives by dissecting the specific contributions of various economic indicators (e.g., health expenditure, tax revenue) and, crucially, governance quality metrics (e.g., government effectiveness, control of corruption, political stability). This approach allows for a more nuanced understanding of how diverse national contexts shape healthy working life, providing actionable insights for policy development that extends beyond mere economic prosperity.” (Page 5, Line 6-15)

**Comment 5:**

Revise: “to identified the individual-level and country-level factors that influencing healthy working lives”. The analysis is not causal.

**Response**:

Thank you for this important comment. We have revised the text according to your suggestion. “This study aimed to estimate HWLE among a multinational population aged 50 and older in 28 countries across three continents, and to examine the associations of individual-level and country-level factors with HWLE” (Page 5, Line 16-18)

**Comment 6:**

The aim is to estimate HWLE in 28 countries but it needs to be stated and elaborated in the discussion why comparing HWLE for 28 countries is useful. Please outline how your analysis explains differences between countries. Should the results and discussion further explore why HWLE differs by health conditions - is this about healthcare provision or as outlined above politics or economy.

**Response**:

We appreciate the reviewer's insightful comment regarding the utility of comparing HWLE across 28 countries and the need to elaborate on how our analysis explains cross-country differences. Due to the poor description, we did not clarify the main purpose of this article in the previous manuscript. We would like to clarify that while our study indeed estimates HWLE in 28 countries, its primary aim extends beyond a mere comparison of absolute HWLE values. Instead, our core objective is to systematically examine the associations of both individual-level and country-level factors with HWLE across this multinational cohort. The corresponding description has been modified as follows:

“This study aimed to estimate HWLE among a multinational population aged 50 and older in 28 countries across three continents, and to examine the associations of individual-level and country-level factors with HWLE.” (Page 5, Line 16-18)

**Comment 7:**

Please also state the novelty of the country level factors; the factors are a measure of wealth. The results indicate that wealthier countries have higher HWLE which is known. The last paragraph indicates that you would explore factors in more depth but this is not achieved. Please re-frame the aim and purpose of this work.

**Response**:

We appreciate the reviewer's valuable feedback regarding the novelty and depth of our analysis on country-level factors, and the suggestion to re-frame the aim and purpose of our work. We agree that simply stating wealthier countries have higher HWLE is a known fact, and our study aims to provide a more nuanced and in-depth understanding beyond this general observation.

We have revised the Introduction and Discussion sections to more clearly articulate the novelty of our approach and the depth of our findings regarding country-level factors. Regarding the novelty of country-level factors: While it is generally understood that economic prosperity correlates with better health outcomes, our study's novelty lies in its disaggregation and quantification of specific economic indicators and, crucially, the inclusion and analysis of governance quality metrics as distinct determinants of HWLE across a multinational cohort. As highlighted in the revised Discussion, we found that:

Beyond overall GDP per capita, tax revenue emerged as a distinct factor more closely linked to maintaining a healthy workforce, suggesting the importance of public funding mechanisms.

Governance quality metrics (government effectiveness, control of corruption, and political stability) were identified as significant independent associations with HWLE. These are not merely proxies for wealth but represent critical institutional and societal conditions that foster better population health and working capacity. This systematic, multinational evaluation of governance's role in HWLE is a key novel contribution.

Regarding the depth of exploration: We acknowledge the reviewer's point about exploring factors in more depth. In the revised Discussion, we have expanded on how these specific economic and governance factors might influence HWLE. For example, robust governance likely contributes through improved public service delivery, social trust, and stability, which are essential for supporting healthy aging and sustained employment. By quantifying the distinct contributions of these specific variables, our analysis moves beyond a general correlation with 'wealth' to provide a more granular understanding of the mechanisms at play. This deeper insight offers more actionable guidance for policymakers, suggesting that targeted interventions in areas like public finance management and governance reform can specifically impact healthy working lives, rather than merely relying on overall economic growth.

Re-framing the aim and purpose: Based on these clarifications, we have refined the statement of our study's aim in the Introduction to emphasize this deeper analytical approach. Our primary aim is not merely to compare HWLE across countries, but to systematically examine and quantify the distinct associations of individual-level and a comprehensive set of country-level factors (including specific economic indicators and governance quality metrics) with HWLE across a diverse multinational population. This allows us to identify which specific aspects of national contexts, beyond general wealth, are most influential in shaping healthy working lives, thereby providing more targeted evidence for health, employment, and retirement policies.

We believe these revisions address the reviewer's concerns by clearly articulating the novel contributions of our country-level factor analysis and demonstrating the depth of understanding our findings provide.

**Comment 8:**

The study has selected factors identified in other studies -what is the novelty of looking at these again - please state that the aim is to compare between countries (and check this is done in the results).

**Response**:

We thank the reviewer for raising this important point regarding the novelty of the factors selected and the emphasis on cross-country comparison. According to your comment 6 and comment 7, we re-framed the core objective is to systematically examine the associations of both individual-level and country-level factors with HWLE across this multinational cohort.

We have revised our Introduction (Page 5, Line 6-15) and Discussion (Page17 Line13- Page18 Line11) sections to state the novelty of our study more explicitly in this context.

As demonstrated in our Results section (e.g., Page 11, Line 4-19, and Supplementary fig 5 & 6, Supplementary table 6), we clearly present and discuss the marked cross-country variations in HWLE and other health expectancies. Furthermore, our analysis of country-level factors (Table 2 and Figure 3) directly addresses how national economic indicators and governance quality contribute to these observed cross-country differences.

While some country-level factors like GDP per capita might have been broadly linked to health outcomes, our study provides novel insights by disaggregating and quantifying the specific associations of various economic indicators (e.g., health expenditure, tax revenue) and, crucially, governance quality metrics (e.g., government effectiveness, control of corruption, political stability) with HWLE. This goes beyond a general understanding that “wealthier countries have higher HWLE” by identifying which specific aspects of national contexts are most influential. This nuanced understanding is critical for developing targeted and effective policies.

We believe this integrated, multinational approach to simultaneously assess a comprehensive set of individual and country-level factors, and to explain the observed cross-country variations, provides significant novelty and a deeper understanding of healthy working life expectancy.

**Comment 9:**

Please state that the 28 countries were selected on data availability. Please outline for SHARE in the appropriate Table, the sample sizes for each European country. Please add in the discussion that not all samples are generalisable. The methods fails to outline how survey weights were used

**Response**:

Thank you for your valuable suggestion. We have added relevant statement. “Using these harmonized data, we constructed a multinational dynamic cohort of 241,622 participants aged 50 years and older from 28 countries, which were included based on data availability.” Also, we added a table to show the sample sizes for each European country. (Supplementary table 3. Sample characteristics of SHARE participants by country). As for the generalizability issue, similar to Comment 23, we have revised Limitation 2 to further clarify. “Second, although the overall sample size across countries was large, this was not a global sample, and the sample size in some countries was relatively small, which may limit the generalizability of the findings. This is particularly important for transition-based multistate models, because the estimates depend on the number of observed transitions between states. When the sample size is small in some countries, the number of specific transitions may be limited, which can lead to unstable transition probability estimates and wider confidence intervals.” (Page 19, Line 10-16)

The methods fails to outline how survey weights were used: Thank you for this important comment. We agree that the original Methods section did not clearly state how survey weights were handled. In the present analyses, no sample weights were applied because this study was based on a multi-cohort design, and the survey weights available across cohorts were constructed using different sampling frameworks and weighting procedures, which limited their comparability and direct integration in pooled analyses. We also note that previous multi-cohort studies have commonly conducted pooled analyses without applying survey weights^[1]^.

Following your suggestion, we have clarified this point in the Methods section and also acknowledged it in the Limitations section of the revised manuscript. The added text is as follows:

“Survey weights were not applied in the analyses due to inconsistent weight construction across cohorts.” (Page 6, Line 4-5)

“Seventh, sample weights were not applied in our analyses, due to the multi-cohort design and inconsistent methods of the construction of weights across cohorts.” (Page 20, Line 6-8)

[1] Zhou Y, Kivimäki M, Holt-Lunstad J, Yan LL, Zhang Y, Wang H, Wang S, Xu X. Stressful life events in childhood and adulthood and risk of physical, psychological and cognitive multimorbidities: a multicohort study. EClinicalMedicine. 2025 May 2;83:103225. doi: 10.1016/j.eclinm.2025.103225. PMID: 40453536; PMCID: PMC12123342.

**Comment 10:**

“in order to assess impacts of country-level factors on life expectancy and health expectancies, we firstly calculated life expectancies for participants stratified by each country-level factor, and then calculated the differences and 95%CI of the differences between high and low level of each country-level factor”. - please clarify. I am unable to reproduce this method clearly from the write-up. I am presuming that each individual in the dataset from one country has the same value for each factor. All participants are then added together, TLE and HWLE is calculated and the stratified by country? Is this circular? As intro I am not sure what this adds to what we know that richer countries have higher HWLE, perhaps we don't know why this is (is it due to how healthcare is funded or workplace health is managed?) - please state.

**Response**:

Thank you for your important suggestion. We agree that our previous description of the analysis of country-level factors was not sufficiently clear. In our analysis, each participant was first assigned the value of the corresponding country-level factor (e.g., GDP) according to their country of residence and the year of cohort entry. These assigned values were then used to dichotomize participants at the median into high- and low-level groups across the pooled sample. The estimates were subsequently calculated for the two groups, and the differences between groups, together with their 95%CIs, were obtained. We have revised the Methods section to clarify this procedure. The revised text is as follows: “The above 8 country-level factors were dichotomized by the median, coded as “high” and “low” by two steps. Data corresponding to the country-level factors in the year when participants were first enrolled in the cohort were used. If data for that year were unavailable, values from the preceding year were used instead. For each factor, participants were then dichotomized into “high” and “low” groups using the median of the assigned values in the pooled sample.” (Page 8, Line 1-6) “Similarly, to assess the associations of country-level factors with life expectancy and health expectancies, each participant was assigned the corresponding country-level value according to the procedure described above. Based on the distribution of the assigned values across all participants, each country-level factor was dichotomized at the median into high- and low-level groups. The corresponding estimates were then obtained for the two groups, and the differences between groups, together with their 95%CI, were calculated.” (Page 9, Line 4-7)

**Comment 11:**

Sensitivity analyses - As outlined previously why compare self-reported health with ADLs - these are not relevant to work and capture a high level of disability, quite different to health

**Response**:

We appreciate the reviewer's insightful comment regarding the comparison of self-reported health (SRH) with Activities of Daily Living (ADL) in our sensitivity analyses. We acknowledge that SRH and ADL capture different aspects of health, with ADL reflecting more severe functional limitations and disability, which may appear distinct from a broader definition of 'health' relevant to work.

We choose ADL as an alternative health definition in the sensitivity analyses based on the following thinking:

1) A primary purpose of sensitivity analysis is to assess the robustness of our main findings to different definitions of key variables. SRH is a widely used and validated measure in health expectancy research, but its subjective nature means it can be influenced by cultural and individual perceptions. By contrast, ADL provides a more objective measure of functional capacity. Using ADL allows us to test whether the associations identified between individual/country-level factors and HWLE hold true when a more stringent, functional definition of health is applied.

2) While ADL limitations represent a high level of disability, these fundamental functional impairments (such as difficulty bathing, dressing, or walking) are undeniably critical determinants of an individual's ability to perform work tasks and maintain employment. Therefore, ADL is highly relevant to the concept of 'healthy working life expectancy,' as severe ADL limitations would almost certainly preclude healthy and sustained employment. It represents a critical threshold of functional health that directly impacts work participation.

3) The use of ADL as an alternative health measure in healthy life expectancy calculations is consistent with established methodologies in the field, particularly for assessing functional health in older populations, such as reference 9 and reference 10.

Our sensitivity analyses, as presented in Supplementary Table 7 and Supplementary Fig 4, showed that while defining health based on ADL led to different absolute HWLE estimates (e.g., higher overall HWLE due to ADL capturing more severe states), the direction and significance of the associations between key individual and country-level factors and HWLE remained largely consistent. This consistency strengthens the confidence in our main findings, demonstrating their robustness across different conceptualizations of health.

We believe that including ADL in our sensitivity analyses provides valuable evidence for the stability of our results under varying health definitions, thereby enhancing the overall credibility of our study.

**Comment 12:**

Finally, to examine the results' sensitivity to varied analysis methods, we used shorter interpolations (12-month step and 18-month step) and longer interpolations (36-month step) to construct transition probability models to calculate TLE and health expectancies - state why you have done these analyses.

**Response**:

Thank you for your valuable suggestion. The IMaCh technical documentation notes that life expectancy estimates are calculated using trapezoidal integration based on fixed monthly intervals (steps), and that the choice of step size can be used to compare differences across model specifications. Therefore, sensitivity analyses using different step sizes are necessary to assess the robustness of the results. The whole paragraph has been revised as followed: “First, to examine whether the results were sensitive to the interpolation interval used in the transition probability models, analyses were repeated using step sizes of 12 months (Method 1), 18 months (Method 2), and 36 months (Method 3). Second, to assess the potential impact of sample selection, we included the 11,465 individuals who had been excluded from the primary analysis due to missing individual-level covariates and re-estimated the results in the intact sample of 186,783 participants (Method 4). We also randomly selected 70% of the analytic sample and repeated the analyses in this sub-sample of 122,722 participants (Method 5). Third, to assess whether the findings were sensitive to the health definition, we used activities of daily living (ADL) as a functional definition of health. Health was defined as good if participants reported no difficulty in bathing, dressing, eating, getting in and out of bed, or walking across a room, and poor if difficulty was reported in at least one of these activities. Based on this alternative definition, analyses were repeated using a 24-month step (Method 6) and a 12-month step (Method 7).” (Page9 Line16- Page10 Line5)

**Comment 13:**

Please remove the work “globally” the datasets do not provide a global sample.

**Response**:

Thank you for your helpful suggestion. We have removed the term “globally” from the manuscript accordingly.

**Comment 14:**

The TLE for Switzerland continues to indicate an issue with the validity of your estimates. The problem appears to be with SHARE data. The estimate is 6 years higher than official estimates - does that mean that all other estimates for Switzerland are around 15% higher than the true value? The methods are overestimating life expectancies for some countries - it means the estimates are difficult to compare because there is a bias which is overestimating the values

**Response**:

We appreciate the reviewer's persistent and critical observation regarding the estimated Total Life Expectancy (TLE) for Switzerland and the potential implications for the validity and comparability of our estimates. We acknowledge that our TLE estimate for Switzerland at age 50 (40.46 years) is indeed higher than some official national statistics, and we understand the reviewer's concern that this might indicate a systematic overestimation affecting other countries or the relative comparisons.

We have carefully considered this point and have added a new point to the Limitations section of our revised manuscript (page20 line12- page21 line8) to explicitly address this issue and provide a more thorough explanation.

Our response is as follows:

We agree that the TLE estimate for Switzerland in our study is higher than some official figures, and we have explicitly acknowledged this potential overestimation in our revised Limitations section. This difference can arise from several methodological distinctions between our study and official life expectancy calculations:

Firstly, official statistics typically rely on comprehensive vital registration systems and census data for entire populations. Our study, however, uses harmonized longitudinal cohort data (e.g., SHARE for Switzerland) which, while robust for studying individual trajectories and risk factors, has specific sampling frames, inclusion/exclusion criteria, and follow-up protocols. These differences in data collection and underlying populations can lead to variations in estimates.

Secondly, the participants in the SHARE cohort, even if broadly representative, might differ from the general population in ways that influence their life expectancy (e.g., healthier participants might be more likely to remain in the study). This interpretation is also supported by our cohort-stratified analysis. The cohort-stratified results showed that the estimated TLE at age 50 in the SHARE cohort was 34.40 years (see Comment 24). This estimate is broadly consistent with previous studies based on SHARE data, which reported TLE at age 50 of 37.87 years for women and 32.65 years for men [2]. Although these figures are not directly comparable because of differences in sample composition and analytical strategy, they suggest that relatively high TLE estimates may also reflect characteristics of SHARE-based cohort data.

Thirdly, as we have also noted in another limitation, any potential underestimation of mortality within our cohort data (e.g., if some deceased participants were misclassified as lost to follow-up) could lead to an upward bias in life expectancy estimates.

Finally, our estimates are specifically for individuals at age 50, using a multi-state Markov model, which might differ from the exact methodology used for official period or cohort life tables.

We recognize that if such an overestimation is systematic across certain datasets or countries, it could indeed affect the absolute values and their direct comparison with external official statistics. We have explicitly stated this caution in the Limitations section.

Despite these potential biases in absolute values, we believe our study's primary strength and contribution lie in identifying relative associations and cross-national patterns of factors influencing HWLE. Our sensitivity analyses (Supplementary Table 7 and Supplementary Fig 4) demonstrate the robustness of the direction and significance of the associations between individual/country-level factors and HWLE, even when different methodological assumptions (e.g., interpolation steps, health definitions) are applied. Therefore, while the absolute values should be interpreted with caution and not directly equated with official national statistics, the identified relationships between factors and HWLE, and the comparative insights into why HWLE varies across countries based on specific individual and country-level determinants, remain valid and valuable.

In summary, we acknowledge the potential for overestimation in absolute TLE values for some countries and have clarified this as a limitation. However, we maintain that the core findings regarding the relative impact of individual and country-level factors on HWLE, and the patterns of cross-national variation, provide robust and novel insights.

[2] Talifu Z, Guo S, Su B, Wu Y, Wang Y, Liu J, Luo Y, Zheng X. Gender disparities in multi-state health transitions and life expectancy among the ≥50-year-old population: A cross-national multi-cohort study. J Glob Health. 2024 Sep 6;14:04156. doi: 10.7189/jogh.14.04156IF: 4.3 Q1 . PMID: 39238364; PMCID: PMC11377966.

**Comment 15:**

I don't feel any of the maps add anything to the results- most of it is grey because the countries are not in the analysis.

**Response**:

Thank you for your valuable suggestion. Thank you for your valuable suggestion. Following your recommendation, we have removed the corresponding figures from the main text. They are now presented only in the Supplementary Information as additional supporting material.

**Comment 16:**

There is very little comparison between countries for the other variables - please consider adding.

**Response**:

Thank you for your valuable suggestion. Following your recommendation, we have added additional comparisons for HnWLE and nHWLE across countries in the Results section. “In addition to HWLE, cross-country differences were also observed in other health expectancy states. HnWLE was relatively high in Switzerland (16.61 years), Austria (15.60 years), Belgium (15.44 years), and Mexico (15.03 years), whereas it was notably low in Estonia (2.29 years), Latvia (2.21 years), and Portugal (3.96 years). nHWLE was particularly high in Portugal (7.11 years) and Estonia (8.66 years), indicating a longer period of life spent in poor health while remaining in work.” (Page 11, Line 18-23)

**Comment 17:**

Supplementary Table 1 indicates that the datasets contributed different number of waves and time periods. Did this place a part in estimates if data was collected at different time points. How would this impact on the country wide comparisons?; are confidence intervals important?

**Response**:

Thank you for your important suggestion. We agree that differences in the number of waves and time periods across datasets may have influenced the estimates and their comparability across countries. Following your suggestion, we have added a clarification in the limitations section to note that such temporal heterogeneity may affect cross-country comparisons. In addition, as stated in the revised Limitation 2, countries with relatively small sample sizes may have wider confidence intervals because transition-based multistate estimates depend on the number of observed transitions between states. Reporting confidence intervals is therefore important, as it allows readers to better interpret the uncertainty of the estimates. “First, the baseline and follow-up periods of the harmonized cohorts varied across countries and regions, and the number of waves also differed across datasets, which may bias cross-country comparisons because health and working states may be influenced by the temporal and policy contexts at baseline and follow-up.” (Page 19, Line 7-10)

**Comment 18:**

Supplementary Table 2 - please stratify SHARE by country - the estimates are by country. As sample size is important please outline the size for each European country included.

**Response**:

Thank you for your valuable suggestion. Following your recommendation, we have stratified the SHARE data by country and added **Supplementary Table 3. Sample characteristics of SHARE participants by country**, which reports the sample size for each European country included in the analysis.

**Comment 19:**

Supplementary Table 4 - TLE estimates do not align with official estimates https://www.europeinnumbers.com/rankings/life_expectancy_at_fifty/ As outlined in the first review this questions the estimates or all of the HWLE states. The differences in TLE to official estimates are significant (e.g. 5 years for Austria as well as for Switzerland in the last review.

**Response**:

We appreciate the reviewer's continued vigilance regarding the discrepancy between our estimated TLE values and official statistics, particularly for Switzerland and Austria, and for providing the external reference. Considering together with your comment 14, We have integrated and strengthened our explanation for this issue in the Limitations section of our revised manuscript. (page20 line12- page21 line8)

**Comment 20:**

“The results corroborate previous findings from England[9], Australia[11], Europe[18], and China[10], indicating that inequalities in HWLE persist among individuals of varying socioeconomic statuses and extending working life remains a formidable challenge.” - outline why the results indicate that it will be a challenge. Was this an opportunity to compare the differences by country between HWLE and state pension provision.

**Response**:

Thank you for your important suggestion. Following your recommendation, we have revised this part of the Discussion. We have also added an illustrative example to further support this point. The revised text reads: “ The results corroborate previous findings from England[9], Australia[11], Europe[18], and China[10], indicating that inequalities in HWLE persist across socioeconomic groups. This suggests that the capacity to remain both healthy and employed is not equally distributed across populations. In particular, individuals with lower socioeconomic status, poorer health, and chronic conditions may have fewer years available to remain in healthy work. Notably, with the exception of Switzerland, HWLE at age 50 was below 15 years in the remaining 28 countries. This finding suggests that, in most countries, policies aimed at further delaying retirement beyond age 65 may create a mismatch between retirement policy and older adults’ actual health and work capacity. For example, in the United States, HWLE was 10.69 years in our study, whereas the current full retirement age in the Social Security system is 67 for people born in 1960 or later[19]. This comparison is illustrative rather than a formal analysis of retirement systems, but it highlights the potential risk that some older adults may be expected to remain in work longer than their healthy working capacity would allow.” (page14 line18- page19 line6)

**Comment 21:**

“Specifically, national health expenditure and GDP per capita emerged as pivotal determinants of longer life expectancy, whereas tax revenue was more closely linked to

maintaining a healthy workforce”. This conforms what we know - countries with higher incomes have higher HWLE. Can you take this any further or does the data limit developing depth.

**Response**:

We appreciate the reviewer's comment that our finding regarding higher HWLE in countries with higher incomes might seem to conform to existing knowledge. We agree that a general correlation between national wealth and health outcomes is well-established. However, our study aims to move beyond this broad observation by providing a more nuanced and actionable understanding of how specific economic and governance factors contribute to healthy working life expectancy.

We have revised the Discussion section of our manuscript (page14 line15- page16 line3; page17 line13- page18 line11) to further elaborate on this point and demonstrate how our findings offer greater depth.

**Comment 22:**

“Our findings, based on self-reported health measures, also 7 show that stroke and chronic lung disease are associated with lower TLE and HWLE, indicating controlling for risk factors leading to stroke and chronic lung disease among adults may benefit to increasing TLE and HWLE.” This is a missed opportunity to compare the relationship with health conditions by country. This would provide a more in-depth discussion on why differences occur between countries and hence potential interventions. Whilst prevention of health conditions is important, HWLE would increase with workplace support - would be worth exploring? Would it also be worth discussing differences between countries and health conditions for HnW, nHW and nHnW?

**Response**:

We appreciate the reviewer's insightful comments, which highlight important avenues for deepening our discussion on the relationship between health conditions and healthy working life expectancy. We agree that these points represent valuable opportunities for further exploration, which have been added in the Discussion section. (page17 line13- page18 line11; page21 line9- page22 line2)

**Comment 23:**

Limitation 2 - expand on why small sample sizes for a methodology that focuses on transitions is a problem in addition. With regards generalisability - can you compare country samples with country distributions?

**Response**:

Thank you for your helpful suggestion. Following your suggestion, we have revised the limitation to further explain why small sample sizes are problematic for transition-based multistate models. “Second, although the overall sample size across countries was large, this was not a global sample, and the sample size in some countries was relatively small, which may limit the generalizability of the findings. This is particularly important for transition-based multistate models, because the estimates depend on the number of observed transitions between states. When the sample size is small in some countries, the number of specific transitions may be limited, which can lead to unstable transition probability estimates and wider confidence intervals.” (Page 19, Line 10-13)

**Comment 24:**

Limitation 5 -cohort effects can be examined for.

**Response**:

We appreciate the reviewer's concise but important comment regarding cohort effects.

We would like to draw the reviewer's attention to the Limitations section of our manuscript (Page 20, Line 4-6), where we have already acknowledged this point: “Sixth, the addition of new participants to each cohort at different times may introduce cohort effects, as disease incidence and prevalence change over time, potentially biasing our estimates.” In addition, following the reviewer’s suggestion, we conducted supplementary analyses stratified by cohort, and the results have been added to Supplementary Table 5. The cohort-stratified results showed that the estimated TLE at age 50 in the SHARE cohort was 34.40 years. This estimate is broadly consistent with previous studies based on SHARE data, which reported TLE at age 50 of 37.87 years for women and 32.65 years for men^[2]^. Although these figures are not directly comparable, they are within a similar range and suggest that our estimates are reasonable. At the same time, this pattern is also consistent with the limitation we acknowledged in the manuscript. As noted in the revised Limitations section, estimated TLE for some countries may appear higher than official national statistics because cohort-based harmonized datasets such as SHARE are designed primarily for cross-national comparability rather than precise national population estimation. Differences from official statistics may arise from cohort inclusion and exclusion criteria, follow-up structure, age-specific estimation methods, and potential underestimation of mortality within cohorts. Therefore, while these supplementary findings support the general validity of our estimates, the absolute values of TLE and HWLE should still be interpreted with caution, particularly when compared directly with official national statistics. (page20 line13- page21 line9)

| **Cohort** | **N** | **TLE** | **HWLE** | **HnWLE** | **nHWLE** | **nHnWLE** |
| --- | --- | --- | --- | --- | --- | --- |
| SHARE | 94,627 | 34.40(34.24-34.55) | 10.71(10.55-10.87) | 10.97(10.84-11.10) | 2.80(2.70-2.90) | 9.92(9.80-10.04) |
| CHARLS | 15,262 | 30.85(30.44-31.27) | 12.76(12.49-13.03) | 7.87(7.62-8.12) | 4.15(3.98-4.32) | 6.08(5.85-6.30) |
| KLoSA | 8,801 | 33.15(32.73-33.57) | 13.46(13.03-13.90) | 11.51(11.08-11.93) | 1.55(1.43-1.67) | 6.63(6.39-6.86) |
| HRS | 36,339 | 30.42(30.24-30.62) | 10.69(10.50-10.88) | 11.16(11.00-11.31) | 1.84(1.77-1.91) | 6.74(6.62-6.85) |
| ELSA | 13,981 | 30.78(30.41-31.14) | 9.94(9.54-10.34) | 13.23(12.87-13.59) | 1.40(1.23-1.57) | 6.20(5.96-6.45) |
| MHAS | 17,773 | 30.58(30.30-30.86) | 11.05(10.73-11.38) | 15.03(14.71-15.36) | 1.05(0.97-1.13) | 3.44(3.31-3.57) |

[2] Talifu Z, Guo S, Su B, Wu Y, Wang Y, Liu J, Luo Y, Zheng X. Gender disparities in multi-state health transitions and life expectancy among the ≥50-year-old population: A cross-national multi-cohort study. J Glob Health. 2024 Sep 6;14:04156. doi: 10.7189/jogh.14.04156IF: 4.3 Q1 . PMID: 39238364; PMCID: PMC11377966.

**Comment 25:**

“Nevertheless, this study provides valuable insights, including the predictive utility of national economic and governance quality indicators for population health and workforce, as well as the influence of chronic diseases on TLE and HWLE, offering potential guidance for measures to extend healthy working life” - please explain how the data provides guidance.

**Response**:

Thank you for this helpful comment. We agree that our previous statement did not clearly explain how the data may provide guidance. We have revised the relevant part. “Nevertheless, this study may help inform strategies to extend healthy working life by identifying factors associated with shorter HWLE at both the individual and country levels. For example, the associations of chronic diseases with lower TLE and HWLE highlight the importance of chronic disease prevention and management, while the associations with health system investment, social inequality, and governance indicators suggest that policy and institutional contexts may shape opportunities to remain healthy and employed at older ages.” (Page 21, Line 9-14)

**Comment 26:**

“In summary, a variety of multilevel interventions are warranted to extend the TLE and HWLE.” This indicates the potential to outline more research -please expand.

**Response**:

Thank you for your helpful suggestion. We agree that the statement on multilevel interventions was too general, and we have revised relevant part accordingly. “At the individual level, prevention and effective management of chronic diseases, particularly diabetes, stroke, and chronic lung disease, along with healthier lifestyles, may help preserve both health and work capacity. At the policy level, greater investment in health systems, efforts to reduce social inequality, and stronger governance may provide the structural conditions needed to support longer and healthier working lives.” (Page 21, Line 21-24)

**Response to Reviewer #3:**

**Comment 1:**

When you write…

Response: “Thank you for this insightful comment. Political Stability and Absence of Violence/Terrorism measures perceptions of the likelihood of political instability and/or politically-motivated violence, including terrorism. Therefore, “Low” Political Stability and Absence of Violence/Terrorism indicates that citizens perceive a lower likelihood of politically motivated violence (including terrorism), which corresponds to a higher TLE. To avoid potential ambiguity, we have added the definitions of each country-level determinant in the supplementary materials (Supplementary 3).”

to my comment…

Comment 3: “On Table 2 (as well as on some Figures) Higher TLE and other values go with “Low” Political Stability and Absence of Violence/Terrorism: Estimate and lower values with “High” Political Stability and Absence of Violence/Terrorism: Estimate. Shouldn't it be the other way around? ”

You go my way, talking about the “likelihood of political instability…” as I suggest changing “low” for “high” and vice-versa. Here in your response, you change “stability” for “instability”. I agree that in English “low” perceptions of the likelihood of political INSTABILITY and/or politically-motivated violence can go with a higher TLE but not “low” perceptions of the likelihood of political STABILITY…”

**Response**:

Thank you for your valuable suggestion. We agree that the previous definitions of “high” and “low” may have caused misunderstanding for readers. After discussion within our team, we decided to follow your suggestion and revise the coding accordingly. Specifically, in the revised manuscript, “high” is now defined as indicating greater political stability and a lower likelihood of political instability and politically motivated violence. We have corrected the relevant content in Figure 4 and Table 2, and we have also added the following clarification in Supplementary 3 of the Supplementary Information: “In this study, higher values indicate greater political stability and a lower likelihood of political instability and politically motivated violence.”

**Response to Reviewer #4:**

**Comment 1:**

The quality of the language is not overall high. E.g., in the abstract, the first sentence lacks a verb and the first section titled background does not describe the background, but focuses rather on the aim of the study. Please let a native speaker read the manuscript. Further example is the sentence on line 13 page 6 of the manuscript, added as an answer to my comment – “Mortality information gained from exit…” - the sentence is not well written.

**Response**:

Thank you for your valuable suggestion. Following your recommendation, we have revised the relevant sections of the manuscript. “Background - To address labor force shortages associated with population aging, many countries have proposed raising the retirement age. As health is an important determinant of employment outcomes among older workers, it is important in this context to understand the individual- and country-level factors associated with healthy working life expectancy (HWLE), defined as the average number of years individuals can expect to remain healthy and employed from age 50.” (Page 2, Line 3-7) “Mortality information was obtained from exit and post-exit interviews conducted with proxy respondents who knew the decreased participants well, such as their spouse, child, or another close person (Supplementary 2).” (Page 7, Line 1-3)

**Comment 2:**

I would agree with the comment#13 of the reviewer 2 that the level of the TE and HWLE is unexpectedly high. I do not find a sufficient acknowledgment of this issue in the limitations section, although the authors mention this in the answer to this comment.

**Response**:

We thank Reviewer 4 for reiterating this important point regarding the unexpectedly high levels of TLE and HWLE, and for highlighting the need for a more sufficient acknowledgment of this issue in the Limitations section. We agree that this concern, previously raised by Reviewer 2, warrants comprehensive discussion within the main text.

We have now significantly expanded and clarified our discussion on this matter in the Limitations section of the revised manuscript. We have explicitly addressed the potential reasons for the observed discrepancies between our estimated life expectancies and official national statistics. The potential reasons are following:

“Official statistics often rely on comprehensive vital registration systems and census data, whereas our study uses cohort data with specific inclusion/exclusion criteria and follow-up periods. Our estimates are for individuals at age 50, which might differ slightly from official life tables that often use period or cohort life expectancies calculated differently. Although the cohorts are large, they may not perfectly represent the entire national population, especially in specific age groups or socioeconomic strata. As noted in a previous limitation, any underestimation of mortality rates within the cohort (e.g., due to lost to follow-up being misclassified) could lead to an overestimation of life expectancies.” (Page20, Line16-24)

**Comment 9:**

My comment 9 (reviewer 4 comment 9) was not answered completely. The question remains open where was the median value itself included - into the high or low-group? I insist on the answer to this question.

**Response**:

We sincerely apologize for this oversight and thank you for pointing it out. For observations exactly equal to the median, we assigned them to the group that resulted in the smallest difference in sample size between the two groups. This approach is consistent with methodological guidance^[3]^ —“Values exactly at the median can be put into either group, and are usually assigned in a way that will make the groups most equivalent in size.”

Following your suggestion, we have now clarified this grouping rule in the Methods section as follows: “For observations exactly equal to the median, values were assigned to the group that resulted in the smallest difference in sample size between the two groups.” (Page 8, Line 6-8)

[3] Gallucci, M. . (2011). Best practices for using median splits, artificial categorization, and their continuous alternatives. Journal of Experimental Psychopathology, 2 (2), 197-209.

**Comment 15:**

my comment 15 (reviewer 4) was not answered appropriately. I insist on including possible biases in mortality assessment into limitations.

**Response**:

Thank you for your valuable suggestion. Following your suggestion, we have added the following limitation to the main text. “Fifth, mortality ascertainment bias may have affected our estimates. If some deceased participants were not identified and were instead treated as lost to follow-up, mortality rates may have been underestimated, which may have led to overestimation of TLE and HWLE.” (Page 20, Line 1-4)
