# Supplementary material for: Country- and individual-level factors associated with healthy working life expectancy: A multinational longitudinal study
Source: PLoS Med. 2026 Sep 10;23(9):e1004676. doi: 10.1371/journal.pmed.1004676 (PMC13561363; doi:10.1371/journal.pmed.1004676)
Supplement: Supplementary file 10 [file pmed.1004676.s010.docx]

Responses to Reviewers’ Comments for Manuscript PMEDICINE-D-25-02153R1

**Country- and individual-level factors associated with healthy working life expectancy: a multinational longitudinal study**

Addressed Comments for Publication to

PLOS Medicine

by

Jingjie Zhu, Bo Ye, Junling Gao

Dear Editors and Reviewers,

We sincerely appreciate the opportunity to revise our manuscript titled “Country- and individual-level factors associated with healthy working life expectancy: a multinational longitudinal study” and are grateful for the insightful comments provided by the reviewers. Those comments are all valuable and very helpful for revising and improving our paper, as well as the important guiding significance to our research. In this response letter, we have provided detailed responses to each of the reviewers' comments. The reviewers' comments are presented in *italics*, and our corresponding changes and additions to the manuscript are highlighted in red text. Additionally, we have conducted a comprehensive revision of the entire manuscript. We have provided both a tracked-changes version of the revised manuscript and a clean version without markup. We have tried our best to make all the revisions clear, and we hope that the revised manuscript meets the requirements for publication.

Thank you again for your time and consideration.

**Response to Reviewer #1:**

***Comment 1:*** *[MINOR] In the abstract the authors say "… chronic diseases, particularly stroke, reduced HWLE by 5·49 years (95%CI:4·94-6·04)". It's not clear to me whether these statistics refer to individuals with any chronic disease or with stroke in particular. Suggest clarifying.*

***Response***: Thank you for pointing out this important aspect. The results indicated major chronic diseases including arthritis, cancer, diabetes, heart problems, hypertension, stroke and chronic lung disease were associated with reduced TLE and HWLE, and stroke was the leading one. According to your suggestion, we have revised the Abstract accordingly.

“Marriage, higher education, and healthy lifestyles were associated with longer HWLE, while chronic diseases reduced HWLE, with stroke showing the greatest reduction of 5.49 years (95%CI: 4.94-6.04).” (Page 2, Line 17-19)

**Comment 2:** [MINOR] While the English in this paper is very good, there are a few instances of unusual wording (e.g., "These individual socioeconomic disparities arise great concerns for policymakers…", "The detail cohorts' information (see Supplementary 1) of the participants by waves…", "duration of interview date") that would benefit from further checking.

***Response***: Thank you for your valuable suggestion. We have revised the relevant sentences to improve readability.

***Comment 3:*** *[MINOR] Suggest the authors very briefly explain what Gini index measures (income inequality?) for those of us who are unfamiliar with it.*

***Response***: Thank you for your helpful suggestion. We have briefly explained the Gini index in the manuscript as follows:

“The Gini index (reflecting the degree of inequality in income or consumption distribution).” (Page 7, Line 9-10)

***Comment 4:*** Otherwise, the Introduction is excellent. Clear, succinct, and comprehensive.

***Response***: Thank you for your positive comments!

***Comment 5:*** *I suggest the authors ensure only study methods are included in the Methods section - any results (including numbers of patients included/excluded and references to main or supplementary figures with patient flow) should be in the Results section.*

***Response:*** Thank you for pointing out this important aspect. According to your suggestion, we have moved the relevant content to the Results section (Page 9, Line 14-19) and revised the Methods section (Page 5, Line 15-17) accordingly.

***Comment 6:***  *Looking at the "doctor diagnosed condition" at the supplied website (https://g2aging.org/vac/home) I noticed quite a few conditions that could foreseeably impact HWLE (e.g., "Alzheimer's disease and Dementia", "Arthritis", "Parkinson's Disease", "Psychological conditions", and others) were available on the website but not included in the analyses. Suggest the authors consider inclusion or justify their reasoning for only analysing a subset.*

***Response:*** We completely agree with you that Alzheimer's disease and Dementia, Arthritis, Parkinson's Disease, Psychological conditions may associate with HWLE. Due to their availability in the database, we did not include these conditions in the analyses. For example, doctor diagnosed dementia was available from Wave 10 in HRS and not available in MHAS. Psychological conditions were not available in MHAS. Parkinson's Disease was not available in CHARLS, KLoSA and HRS. In order to include as many participants as possible, we only analyzed doctor diagnosed conditions which were available in all cohorts. According to your suggestion, the related description was revised as following:

*“Due to availability, hypertension, diabetes, cancer, lung disease, heart problems, stroke, and arthritis, which were available in all cohorts were included in our analyses.”* (Page 6, Line 21-23)

***Comment 7:*** *[MINOR] The Statistical Analysis section of the Methods is well-presented, and I appreciate the additional information provided in the Supplement. I am not sure of the value of including the 95%CI formula given a reference is provided (formulae can scare some readers off), however I will leave this decision to the authors.*

***Response:*** Thank you for this helpful suggestion. According to your advice, we have removed the formula from the manuscript and retained only the reference.

“The 95% CI of the difference was calculated based on the square root of the sum of variances under the assumption of independence[16].”(Page 8, Line 18-19)

***Comment 8:*** *The authors could consider explicitly noting any important assumptions underpinning the discrete-time Markov multi-state model-based methods used. Also, being clear on whether transition probabilities were assumed constant over time or whether they were allowed to vary by age.*

***Response:*** Thank you for this valuable comment. In our study, the model assumes that an individual’s transition between states depends only on the current state and covariates (Markov assumption). The transition probabilities were allowed to vary by age but were assumed to remain constant within each time interval (24 months in the main analysis). According to your suggestion, we have added this clarification in the Statistical Analysis section of the Methods. (Page 8, Line 1-6)

***Comment 9:*** *Another concern I have is it's very difficult to keep track of all the explanatory variables across individual and country levels. I would encourage the authors to include a table or figure (supplementary is fine) that lists all the variables in one place and clearly shows when they are first recorded and if/when they are updated during follow-up.*

***Response:*** Thank you for your valuable suggestion. We have added a supplementary table to present the definitions and the timing of the first record for both individual-level and country-level variables (**Supplementary 3**).

***Comment 10:*** *With respect to dichotomising various continuous variables (e.g., country-level GDP), while this historically has been common practice, recent methodological guidance discourages this ( https://doi.org/10.1136/bmj.332.7549.1080). I suggest the authors consider modelling continuous predictors as continuous variables.*

***Response:*** Thank you for this important comment. Due to the methodological limitations of the multistate model, including GDP as a continuous variable would not allow us to estimate the differences in life expectancy between groups. Following your suggestion, we categorized GDP into tertiles and re-ran the analysis. The results were consistent with those from the binary classification, confirming the robustness of our findings.

|  | **N** | **TLE** | **HWLE** | **HnWLE** | **nHWLE** | **nHnWLE** |
| --- | --- | --- | --- | --- | --- | --- |
| **GDP per capita (constant 2015 US$)** | | | | | | |
| Low | 59,747 | 32.03(31.85-32.21) | 10.14(9.97-10.30) | 10.30(10.15-10.45) | 2.57(2.48-2.67) | 9.02(8.91-9.14) |
| Intermediate | 65,520 | 33.60(33.45-33.76) | 10.53(10.36-10.70) | 12.46(12.32-12.61) | 2.31(2.24-2.38) | 8.30(8.17-8.43) |
| High | 50,051 | 35.12(34.94-35.29) | 12.59(12.40-12.78) | 13.94(13.77-14.11) | 1.99(1.91-2.07) | 6.60(6.48-6.71) |

***Comment 11:*** *Similarly, I found the Sensitivity Analysis section unclear. I suggest the authors make much clearer what each of the seven sensitivity analysis is changing as compared to the main analysis. This could be done textually in the current section or in a supplementary table. It should also be very clear that results are presented for each of the specified sensitivity analyses.*

***Response:*** Thank you for this important comment. We have revised **Supplementary fig 4** to more clearly illustrate the differences among the seven sensitivity analyses in terms of steps, sample sizes, and health definitions. The corresponding results and 95% CI for each sensitivity analysis are now provided in **Supplementary table 5**.

Supplementary fig 4. Results of sensitivity analyses

***Comment 12:*** *Remember to make sure each table and figure is comprehensible without referring to other parts of the manuscript, particularly when using abbreviations. The authors have done this for the first two main tables but not for all tables and figures in the main manuscript and supplementary materials. It is particularly important for observational studies like this one where tables and figures in the supplement are referred to quite a lot.*

***Response:*** Thank you for this helpful suggestion. We have revised both the Tables, Figures and the Supplementary Information accordingly.

***Comment 13:*** *Suggest the authors ensure that rows and columns of tables are appropriately labelled - this is especially important for tables like Supplementary Table 3 with similar variables on both axes.*

***Response:*** Thank you for this helpful suggestion. We have revised both the Manuscript and the Supplementary Information accordingly. For example, we have added a note to **Supplementary table 3** to clarify the meaning of rows and columns: “Rows represent the starting state at age 50, and columns represent life expectancy in each specific state. TLE: Total life expectancy; HWLE: Healthy and working life expectancy; HnWLE: Life expectancy in healthy and not working status; nHWLE: Life expectancy in not healthy and working status; nHnWLE: Life expectancy in not healthy and not working status.”

***Comment 14:***  *Suggest avoiding the word "significant" unless specifically referring to statistical significance e.g., "HWLE showed a significant upward trend with increasing educational attainment…", "Although no significant difference in TLE was observed between drinkers… ". If the authors mean statistical significance, then appropriate statistics should be presented. This is problematic in the Sensitivity Analysis results where the authors claim "…no statistically significant discrepancies…" without presenting any statistics.*

***Response:*** *Yes! We have revised all descriptions with caution in using “significant”*

***Comment 15:***  *Relatedly, all comparisons presented are descriptive i.e., results are presented alongside each other with no quantification or analysis of the difference e.g., "Women had a higher TLE (35·29 years [95%CI: 35·16 to 35·42]) than men (31·72 years [95%CI: 31·58 to 31·87]).". Was this a deliberate choice by the authors or a limitation of the methods used? Either is ok, I just think it might be worth being clear on this in the methods.*

***Response:*** Thank you for your valuable comment. Exactly, IMaCh cannot directly test the significant difference, so we evaluated whether there are significant differences between groups based on the overlap of 95% CIs. The related description was added in the revised manuscript. As following:

*“For comparisons between groups, if the 95% CIs do not overlap, it suggests a statistically* *significant difference.”* (Page 8, Line 19-20)

***Comment 16:*** *Is not clear to me whether "doctor-diagnosed diseases" are updated after enrolment. My earlier comment about making this clearer across all explanatory variables will resolve this but if the diseases are not updated after enrolment, I suggest the authors include this as a limitation because these obviously emerge over a lifetime.*

***Response:*** Thank you for this important comment. In our study, because information on doctor-diagnosed diseases was only available at baseline in some databases, these variables reflected participants’ disease status at enrollment and were not updated during follow-up. According to your suggestion, we have added this clarification to the Limitations: *“Fourth, information on doctor-diagnosed diseases was collected at baseline and not updated during follow-up. Changes in disease status over time, including the onset or remission of diseases, may have affected the estimation of transitions. ”* (Page 16, Line 17-19)

***Comment* *17****:*  Otherwise, the Discussion is excellent - well done!

***Response:*** *Thank you for your positive comment!*

**Response to Reviewer #2:**

***Comment 1***: First sentence - please revise for clarity.

***Response***: Thank you for your valuable suggestion. In order to improve readability, we have revised the sentence as following:

*“Globally, accelerating population aging and increasing life expectancy have led to a relative shortage of the productive labor force compared with a growing number of retirees.”* (Page 4, Line 2-3)

***Comment 2:*** Last sentence of first paragraph - please elaborate on why gender, educational attainment, regional governance can guide policymakers. Please also reference why a flexible and equitable reitrement system should be a goal - I am not sure what this would look like or is feasible.

***Response***: Thank you for your valuable comment. This study aims to estimate total life expectancy (TLE) and HWLE among a multinational population aged 50 and older in 28 countries across three continents, and to identified the individual-level and country-level factors that influencing healthy working lives. We hope our findings, especially the factors associated with HWLE providing reference information to policies, such as the State Pension age. According to your suggestions, we carefully checked the Introduction section again, finding the background section is somewhat inappropriate, so we have revised the whole Introduction section.

***Comment 3:*** Second paragraph as last point please outline what equitable pension policies are.

***Response:*** Thank you for your valuable comment. According to your ***Comment 2,*** we have revised the whole Introduction section.

***Comment 4:*** Last paragraph of introduction: Is the sample globally representative? Please provide evidence, using data.

***Response***: We completely agree with your comment. Although the sample of each cohort may be a representative sample of population where the cohort were conducted, harmonized them into our study sample is not a global representative sample. Referring to the sample as "global representative" was indeed inappropriate, so we have removed the word "global" from the relevant description. The last paragraph of introduction has been revised as following:

*“T**his study aimed to estimate total life expectancy (TLE) and HWLE among a multinational population aged 50 and older in 28 countries across three continents, and to identified the individual-level and country-level factors that influencing healthy working lives.”* (Page 5, Line 5-7)

***Comment 5***: Please revise the second last sentence - there are a large number of factors associated with lower HWLE. Please note that these are associations not determinants (the analysis does not indicate causation).

***Response:*** Thank you for your valuable comment. We have revised the word "determinants" to "factors" in all relevant descriptions.

***Comment 6***: Please justify why the comparison between countries informs strategies - unclear how it will do this at this point.

***Response***: Thank you for your valuable comment. The related descriptions have revised as following:

*“Fully examining the associations between individual- and country-level factors and HWLE can deepen understanding of the relationships between politics, economy, employment, and health, and guide the development of country-specific measures, such as healthcare investment policies, governance capacity building, and the optimization of employment and retirement policies.”* (Page 4, Line 24)

***Comment 7*:** The Global Ageing data is a strength for harmonising data from different countries. The introduction claims that this is a globally representative population (of adults aged 50 years and over) - provide the data to support this - does data from US, England, Europe, Korea, Mexico and China represent the global population? Please also consider how you will support that each of these populations is representative of the country; data in supplementary table 4 questions this.

**Response:** As your above comment 5 on Introduction section. We completely agree with your comment. Although the sample of each cohort may be a representative sample of population where the cohort were conducted, harmonized them into our study sample is not a global representative sample. Referring to the sample as "global representative" was indeed inappropriate, so we have removed the word "global" from the relevant description. The last paragraph of introduction has been revised as following:

*“This study aimed to estimate total life expectancy (TLE) and HWLE among a multinational population aged 50 and older in 28 countries across three continents, and to identified the individual-level and country-level factors that influencing healthy working lives.”* (Page 5, Line 5-7)

As for whether the each of these populations is representative of the country, we got the related information from the published Cohort Profiles as following:

1. HRS: “The Health and Retirement Study (HRS) is a nationally representative longitudinal survey of more than 37 000 individuals over age 50 in 23 000 households in the USA.” [1]

2. ELSA: “The English Longitudinal Study of Ageing (ELSA) is a panel study of a representative cohort of men and women living in England aged ≥50 years.” [2]

3. SHARE: “SHARE is a unique panel database of micro data on health, socio-economic status and social and family networks covering most of the European Union and Israel.” [3]

4. KLoSA: “Participants were selected randomly using a multistage, stratified probability sampling design to create a nationally representative sample of community-dwelling Koreans aged 45 years or older. Participant selection was performed by the Korea Labor Institute for these rapidly growing populations and included individuals from both urban and rural areas.” [4]

5.MHAS: The Mexican Health and Aging Study (MHAS) was designed to prospectively evaluate the impact of disease on the health, function and mortality of adults over the age of 50 in both urban and rural areas of Mexico. The MHAS 2001 baseline is a nationally and urban-rural representative survey of individuals born in 1951 or earlier.[5]

6. “The China Health and Retirement Longitudinal Study (CHARLS) is a nationally representative longitudinal survey of persons in China 45 years of age or older and their spouses, including assessments of social, economic, and health circumstances of community-residents.” [6]

[1] Sonnega A, Faul JD, Ofstedal MB, Langa KM, Phillips JW, Weir DR. Cohort Profile: the Health and Retirement Study (HRS). Int J Epidemiol. 2014;43(2):576-585. doi:10.1093/ije/dyu067

[2] Steptoe A, Breeze E, Banks J, Nazroo J. Cohort profile: the English longitudinal study of ageing. Int J Epidemiol. 2013;42(6):1640-1648. doi:10.1093/ije/dys168

[3] Börsch-Supan A, Brandt M, Hunkler C, et al. Data Resource Profile: the Survey of Health, Ageing and Retirement in Europe (SHARE). Int J Epidemiol. 2013;42(4):992-1001. doi:10.1093/ije/dyt088

[4] Jang SK, Kim JH. Association between Hand Grip Strength and Self-Rated Health in Middle- and Old-Aged Korean Citizens. Korean J Fam Med. 2020;41(1):53-60. doi:10.4082/kjfm.18.0200

[5] Wong R, Michaels-Obregon A, Palloni A. Cohort Profile: The Mexican Health and Aging Study (MHAS). Int J Epidemiol. 2017;46(2):e2. doi:10.1093/ije/dyu263

[6] Zhao Y, Hu Y, Smith JP, Strauss J, Yang G. Cohort profile: the China Health and Retirement Longitudinal Study (CHARLS). Int J Epidemiol. 2014;43(1):61-68. doi:10.1093/ije/dys203

***Comment 8*:** Outcome - the intro indicates that HWLE should be measured with reference to functional capacity, but the outcome is based on self-reported health. Will this reduce the strength of this work as it does not refer to functional/work capacity? How will this link with health and work policy.

***Response:*** we completely with your concern about the definition of HWLE. In this study, based on Lievre A et.al definition [1] and previous studies [2,3], healthy working life expectancy was defined as is the average number of years a person is expected to be healthy and in work from age 50 years(Page 4, Line 10). So, we measured health status based on self-rated health. Of course, functional/work capacity may impact work and health, which is a limitation of this study we mentioned in the limitation section.

[1] Lievre A, Jusot F, Barnay T, et al. Healthy working life expectancies at age 50 in Europe: a new indicator. J Nutr Health Aging. 2007;11(6):508-514.

[2] Parker M, Bucknall M, Jagger C, Wilkie R. Population-based estimates of healthy working life expectancy in England at age 50 years: analysis of data from the English Longitudinal Study of Ageing. Lancet Public Health. 2020;5(7):e395-e403. doi:10.1016/S2468-2667(20)30114-6

[3] Li C, Wang L, Ding L, Zhou Y. Determinants and inequities in healthy working life expectancy in China. Nat Med. 2024;30(11):3318-3326. doi:10.1038/s41591-024-03184-3

***Comment 9*:** Individual-level determinants - please outline why these determinants were selected.

***Response:*** Thank you for your suggestion! Previous studies have found that gender, education, region, deprivation, income, chronic diseases, lifestyle factors were associated with HWLE among population in UK and China [1,2]. So, based on the previous findings, we selected gender, educational attainment, marital status, health behaviours (smoking, drinking, and physical activity), and chronic diseases (hypertension, diabetes, cancer, lung disease, heart problems, stroke, and arthritis) as individual-level factors.

According to your suggestion, the related description in the revised manuscript has revised as following:

*“Based on previous studies[9, 10], The following individual-level factors were included in this study: gender, educational attainment, marital status, health behaviours (smoking, drinking, and physical activity), and chronic diseases (hypertension, diabetes, cancer, lung disease, heart problems, stroke, and arthritis)”*

[1] Parker M, Bucknall M, Jagger C, Wilkie R. Population-based estimates of healthy working life expectancy in England at age 50 years: analysis of data from the English Longitudinal Study of Ageing. Lancet Public Health. 2020;5(7):e395-e403. doi:10.1016/S2468-2667(20)30114-6

[2] Li C, Wang L, Ding L, Zhou Y. Determinants and inequities in healthy working life expectancy in China. Nat Med. 2024;30(11):3318-3326. doi:10.1038/s41591-024-03184-3

***Comment 10*:** Statistical analysis - The description of the analysis needs to be clearer and transparent. It is unclear how variables are being included in the analysis; this needs to be clearly stated. For individual factors; it needs to be clear that HWLE is calculated for each category of each variable. It is unclear how HWLE by country is estimated (stratified or for each country); the methods section should clearly state this.

Please clarify in the methods which population is stratified for health condition and lifestyle factors.

Please state the time period for transitions.

***Response:*** Thank you for your constructive suggestion! Our analysis plan is referred the previous studies (Ref. 9 and Ref. 10). Due to space constraints in the article, we did not describe the analysis plan in detail. In the revised manuscript, we placed the measurements of country-level factors into supplementary file, and described the analysis plan in detail as following according to your suggestion:

*“Firstly, life expectancies were calculated overall for all participants and then stratified by country. In order to assess impacts of individual-level factors on life expectancies, we firstly calculated life expectancies for participants stratified by each individual-level factor, including gender, educational attainment, marital status, health behaviours (smoking, drinking, and physical activity), and chronic diseases (hypertension, diabetes, cancer, lung disease, heart problems, stroke, and arthritis), and then calculated the differences of life expectancies and 95%CI of the differences between levels of each individual-level factor. Similarly, in order to assess impacts of country-level factors on life expectancy and health expectancies, we firstly calculated life expectancies for participants stratified by each country-level factor, and then calculated the differences and 95%CI of the differences between high and low level of each country-level factor. The 95% CI of the difference was calculated based on the square root of the sum of variances under the assumption of independence[16]”* (Page 8, Line 8-19)

***Comment 11*:** Please state why the particular sensitivity analysis is being performed; previously references indicate that self-rated health is similar to functional loss. Please state why you selected a more severe form of functional loss (similar to functional loss in the oldest old!) for a sensitivity analysis?

***Response:*** Thank you for your suggestion! Actually, we used three types of sensitivity analyses. First one is to verify whether the results were sensitive to the health definition; second one is to examine the impact of participant loss to follow-up on the results; third one is to examine the results’ sensitivity to varied analysis methods.

In order to clarify the reason of each sensitivity analysis, we revised the section as following:
*“In order to the robustness of the findings, seven sensitivity analyses were performed in all participants. First, to verify whether the results were sensitive to the health definition, health based on activities of daily living was defined as good if there were no difficulties with activities of daily living and poor if difficulty was reported with at least one activity from bathing, dressing, eating, getting in and out of bed, and walking across a room. Second, to examine the impact of participant loss to follow-up on the results, participants with missing individual-level factors have also been included to exterminate TLE and health expectancies. Finally, to examine the results’ sensitivity to varied analysis methods, we used shorter interpolations (12-month step and 18-month step) and longer interpolations (36-month step) to construct transition probability models to calculate TLE and health expectancies.”* (Page 8, Line 23)

As your concern on health assessment based on activities of daily living, refer to previous studies (Ref.9 and Ref.10) to verify whether the results were sensitive to the health definition. The results showed that defining health as the absence of difficulty with ADL led to higher HWLE overall (11.72 years; 95%CI: 11.63-11.81) and lower estimates for time spent not healthy and in work (1.46 years; 95%CI:1.43-1.49), which was consistent with the findings of Ref.9. Both findings indicated that “*poor health based on activities of daily living was a restrictive measure resulting in low numbers of years expected to be spent not healthy and in work; higher estimates of healthy working life expectancy based on this definition might arise from classifying people with other limiting health problems as healthy*”, which have added in the discussion section of the revised manuscript.

***Comment 12:*** *Supplementary table 2 - suggests that this is a less deprived population than the global average - please comment on this*

***Response***: thank you! Thinking together with your above comments, the participants in this study is not the sample globally representative, which is a limitation of this study, which has been mentioned in the limitation section of the revised manuscript.

***Comment 13:*** *The average total life expectancy seems high - 33.56 years worldwide! All of the life expectancies for the European countries (using SHARE data) are 5 years higher than those reported by government agencies (https://www.europeinnumbers.com/rankings/life_expectancy_at_fifty/). This data reports that TLE for Switzerland is 40.46 years (that is men and women on average from age 50 live until they are 90.46 years; this suggest that a large proportion of people live beyond 90 years). Is this true? If TLE if being over-estimated are all other estimates too high?*

***Response:*** Thank you for your valuable comment. We also noticed the discrepancy between our results and official reports, which was led by two reasons. Firstly, the different estimate methods may lead to this discrepancy. A previous study indicated that estimates of health expectancy in a dynamic situation can be very misleading when based on the Sullivan method.[1] In order to capture the dynamic feature of health and working, we used Discrete-time Markov multistate models to estimate health expectancy, which also used in previous studies (Ref. 9 and Ref.10). Ref. 9 using ELSA estimated the TLE and HWLE at age 50 to be 31.76 years and 9.42 years (95%CI: 9.19–9.66), respectively. In our study, the corresponding estimates were 30.78 years for TLE and 9.94 years (95%CI: 9.54–10.34) for HWLE. Secondly, the participants being evaluated may also be different between official reports and our study. Official reports may be conducted among a more representative group of people, while our study was conducted among a sample population. We also checked the data again, and found that 84.86% of participants’ health status in Switzerland was “good” based our definition.

Meanwhile, wo also this is a limitation of this study, which has been mentioned in limitation section of the revised manuscript.

[1] Barendregt JJ, Bonneux L, Van der Maas PJ. Health expectancy: an indicator for change? Technology Assessment Methods Project Team. J Epidemiol Community Health. 1994;48(5):482-487. doi:10.1136/jech.48.5.482

***Comment 14:*** *Linked to this current smokers can still expect to live on average to 78 worldwide? Please check the TLE estimates.*

***Response*:** Thank you for this valuable comment. According to your suggestion, we checked the analyses again, and still found the TLE among current smokers at age 50 was 28.34 years, corresponding to a total life expectancy may be 78.34 years. In addition, we examined the difference in life expectancy between non-smokers and current smokers, found that non-smokers had a 7.70-year (95% CI: 7.45-7.95) longer life expectancy than current smokers. We also reviewed previous literature to validate our TLE estimates. A large-scale study[1] including 296,836 participants reported that current smokers had a life expectancy of 38.5 years at age 40, equivalent to a total life expectancy of approximately 78.5 years. Another Framingham Heart Study[2], published in European Heart Journal, reported that non-smokers live 8.66 (95% CI: 7.61-9.63) years longer in men and 7.59 (95% CI: 6.33-8.92) years longer in women compared with smokers.

[1] Ozasa K, Katanoda K, Tamakoshi A, et al. Reduced life expectancy due to smoking in large-scale cohort studies in Japan. J Epidemiol. 2008;18(3):111-118. doi:10.2188/jea.je2007416

[2] Mamun AA, Peeters A, Barendregt J, et al. Smoking decreases the duration of life lived with and without cardiovascular disease: a life course analysis of the Framingham Heart Study. Eur Heart J. 2004;25(5):409-415. doi:10.1016/j.ehj.2003.12.015

***Comment 15:*** *Why provide estimates for different states at age 50? Would policy interventions not target these states in the overall population?*

***Response:*** *Thank you for your comment. There is no special purpose, but the estimates also indicated starting states have some impacted on HWLE.*

***Comment 16:*** *Supplementary data table 4 - Please advise on the interpretation of estimates for countries with small sample sizes (e.g. Latvia). The confidence intervals for TLE span 18 years, HWLE span 6 years. I am presuming that small numbers made some transitions between the five states (hence the large CI). Is the small number an issue for representativeness for that country?*

***Response****: We completely agree with your comment. Sample size in some countries (e.g. Latvia) is small, which may biases the corresponding estimates in these countries. This limitation also was mentioned in the limitation section of the revised manuscript. Thank you again for pointing this limitation!*

***Comment 17:*** *Page 10 - should it be that physical inactivity is associated with lower TLE?*

***Response***: Thank you for this important comment. We appreciate your careful reading. This was a typographical error in the previous version, and we have corrected it according to your suggestion. “Physical activity was associated with a longer TLE (2·96 years [95%CI: 2·75 to 3·17]) and a longer HWLE (1·93 years [95%CI: 1·72 to 2·14]) at age 50.” (Page 11, Line 4-5)

***Comment 18***: Please expand on why this data will raise concerns for delaying retirement. It is not outlined why HWLE is important as an indicator. Can it ever be equivalent to retirement age?

Please expand on why the indicator is important - it is unclear.

***Response***: Thank you for your valuable comment. We think that the HWLE indicator is not equivalent to the retirement age but provides information about expected working lives among the pre-retirement population. Notably, our study shows that, except for Switzerland, HWLE estimates for 50-year-olds in the remaining 32 countries were below 15 years. We have added relevant details in the first section of the discussion. These details are as follows:

*“Poor physical and mental health increase the risk of absenteeism, presenteeism, work disability, early retirement, and increased employer costs. At age 50, many individuals are in mid-to-late career stages and may begin to experience age-related health problems that affect work capacity and productivity. Assessing the health status of this workforce is essential for developing national policies to extend working lives. Estimating HWLE at age 50 offers several advantages: it provides a comprehensive view of population health and labor-force participation, informs the feasibility of policies to prolong working lives, and facilitates comparison with findings from other studies. The results corroborate previous findings from England, Australia, Europe, and China, indicating that inequalities in HWLE persist among individuals of varying socioeconomic statuses and extending working life remains a formidable challenge. Notably, the study shows that, except for Switzerland, HWLE estimates for 50-year-olds in the remaining 32 countries were below 15 years. This finding suggests that, in most countries, policies aimed at further delaying retirement beyond the age of 65 may raise concerns, because this group would likely remain in work while in poor health.”* (Page 13, Line 5-19)

***Comment 19:*** The relation between TLE, HWLE and indicators of countries economies is not novel (that is, richer countries with greater governance have healthier populations and better job opportunities). Please outline why this analysis is novel.

***Response:*** Thank you for your valuable comment. We must acknowledge that this study on the relation between TLE, HWLE and indicators of countries’ economies is not original innovation. As you pointed out that richer countries with greater governance have healthier populations and better job opportunities. To the best of our knowledge, there was few studies to quantitively examine the impacts of country-level factors, especially ease of doing business, government effectiveness,	control of corruption, and political stability and absence of violence/terrorism on HWLE.

According your suggestion, we revised the related paragraph of the introduction section as following:

*“It is well known that country-level factors, such as gross domestic product (GDP) per capita, national health expenditures, quality of governance, and the Gini index are significantly correlated with population health. However, no study has sought to identify* *country-level factors of HWLE.* *Fully examining the associations between individual- and country-level factors and HWLE can deepen understanding of the relationships between politics, economy, employment, and health, and guide the development of country-specific measures, such as healthcare investment policies, governance capacity building, and the optimization of employment and retirement policies.”* (Page 4, Line 21)

***Comment 20***: Low and middle income countries is discussed; what LMIC are included in this analysis?

HWLE for HNW, NHW and NHNW in highly dependent on employment opportunities. Please expand on this.

***Response:*** Thank you for your valuable comment. We have added relevant details in the second section of the discussion. These details are as follows:

*“For example, China as one of the world’s most populous LMICs undergoing rapid population aging exhibits marked interregional economic imbalance that have producted substantial regional disparities in HWLE. This underscores the inadequacy of a “one-size-fits-all” retirement age policy. In contrast. policies such as promoting phased retirement programs, enhancing fair hiring practices, expanding employment opportunities, and offering flexible work arrangements would be feasible approaches.”* (Page 14, Line 8-14)

***Comment 21***: Comparison of results with other studies; as outlined above, please comment on the high TLE and the impact on HWLE. Are these estimates of HWLE too high?

Please discuss HWLE estimates for some conditions. HWLE for someone with a stroke seems high across the countries covered.

***Response***: Thank you for your valuable comment. As noted in our discussion, our findings are consistent with those of other comparable studies. Overestimation of total life expectancy (TLE) may result from survival bias in the analytical sample. A further potential explanation is the estimation approach: estimating life expectancy from age 50 may explicitly overestimate values because younger dead who were significantly influence population TLE, were excluded from the estimation. HWLE estimates may be subject to a similar overestimation bias.

As for the impacts of chronic conditions on HWLE, we found that while chronic diseases reduced HWLE, with stroke showing the greatest reduction of 5.49 years (95%CI: 4.94-6.04). we revised the related discussion as following:

*“Our findings, based on self-reported health measures, also show that stroke and chronic lung disease are associated with lower TLE and HWLE, indicating controlling for risk factors leading to stroke and chronic lung disease among adults may benefit to increasing TLE and HWLE.”* (Page 15, Line 6-9)

***Comment 22***: Please discuss limitations in the data collected. Does employment mean it includes working 1 hour or more? How will this bias results?

***Response***: Limitations in the data collected was mentioned in the revised manuscript according to your suggestion.

***Comment 23***: The introduction highlights the importance of function as a measure of health but self-reported health is used. Can people have good self-reported health but have limited work capacity?; work capacity being crucial for policy development.

***Response*:** Thank you for your valuable comment. An optimal definition of HWLE focuses on assessing work ability limitations. As you noted, some individuals may perceive themselves as good health, yet their work ability is limited. This discrepancy could lead to overestimation of HWLE estimates, meaning the group’s actual remaining healthy working life may be shorter than estimated. Moreover, studies of HWLE have shown that SRH-based HWLE estimates are comparable to those derived from measures of limiting long-standing illness or diagnosed chronic conditions. The application of these measures is mainly limited by the limitations of the available data.

***Comment 24***: Please comment on the limitation of representativeness of the overall sample and each country.

As above, I disagree with the statement in the last paragraph (page 14) that TLE is higher because of earlier retirement ages; this must be an issue linked to selection of participants or how calculated. Non-representative samples for countries may explain this. Please comment on the representativeness of the data for countries.

***Response:*** Thank you for your comment. The representativeness of the research subjects may one of challenges all epidemiology studies. Although this study used data from multinational cohorts, the representativeness of the overall sample and each country is not particularly good, which may bias the estimates. This limitation was mentioned in the revised manuscript according to your suggestion.

***Comment 25***: Limitations - please expand on what you mean by "confounding cross-country comparisons"? What is the direction of the measurement error for self-reported information? What data supports cohort effects? Please expand on why two variables could not be included in the analysis

***Response*:** Thank you for your comments. Because participants in each cohort were enrolled the studies at the same time, which may bias the estimates. Such as initial participants in HRS start being interviewed in 1992, while initial participants in CHARLS in 2011. Duo to poor English, we did not clearly present this limitation in the previous manuscript. This limitation has been revised as following:

*“First, the baseline and follow-up periods of the harmonized cohorts varied across countries and regions, which may bias the comparisons among countries because the health and working states may be influenced by the country contexts at baseline and follow-up.”* (Page 16, Line 5-8)

Cohort effects describe how studying populations in different “cohorts” — having been born in a different time or region or having different life experiences — can alter the outcomes of studies. For example, in HRS, participants aged 50 years enrolled in 1992 and participants aged 50 years enrolled in 2010 may exhibit distinct behavioral, health, or economic characteristics.

In order clarify the limitations of this study, according to your comments, the limitation section was revised as following:

*“First, the baseline and follow-up periods of the harmonized cohorts varied across countries and regions, which may bias the comparisons among countries because the health and working states may be influenced by the country contexts at baseline and follow-up. Second, although the overall sample size is large from multinational countries, which is not a global sample, especially sample size in some countries is small, which may impact the generalization of conclusions. Third, SRH-based HWLE estimates are comparable to those derived from measures of limiting long-standing illness or diagnosed chronic conditions[9, 10], but self-reported measures of health and working status are susceptible to measurement error. The definition of work was inclusive and did not differentiate full-time workers from people who work part time. Moreover, work ability limitations are an outcome of poor health. Some individuals may perceive themselves as good health, yet their work ability is limited. This discrepancy could lead to misestimation of HWLE estimates. Fourth, information on doctor-diagnosed diseases was collected at baseline and not updated during follow-up. Changes in disease status over time, including the onset or remission of diseases, may have affected the estimation of transitions. Fifth, the addition of new participants to each cohort at different times may introduce cohort effects, as disease incidence and prevalence change over time, potentially biasing our estimates. Additionally, throughout the life course, individual intrinsic capacity, relevant environmental characteristics, and the interactions between individuals and their environments are key determinants of healthy aging and functional ability[12, 28]. However, due to methodological constraints, we were unable to clarify the effects of these interactions on HWLE, which represents an important area for future research.”*

***Comment 26***: Please give more detail in the novel guidance this has for policy. Is government policy n the countries included not already aiming to reduce smoking, alcohol use, increase activity etc and better manage economies and health?

***Response****:* Thank you for your comments. Our inaccurate descriptions may lead to your misunderstanding. Sorry for this! In this section, we want to summarize some insights for extending health working life based our findings, not to guide policy development. Based your valuable comments, we give care though to the descriptions in this section and think “policy development” may be not appropriate. So, we revised this section as following:

*“Nevertheless, this study provides valuable insights, including the predictive utility of national economic and governance quality indicators for population health and workforce, as well as the influence of chronic diseases on TLE and HWLE, offering potential guidance for measures to extend healthy working life. Health promotion programs focused on preventing chronic diseases (i.e. stroke and chronic lung disease), controlling tobacco use, and promoting physical activity have the potential to extend both life expectancy and healthy working years. At the national level, strategies to boost economic development and improve governance quality, such as increasing tobacco taxes to fund sports facility construction may be beneficial to population health; improving ease of doing business may provide more job opportunity. In summary, a variety of multilevel interventions are warranted to extend the TLE and HWLE.”* (Page 17, Line 3-12)

**Response to Reviewer #3:**

***Comment 1:*** *In the section "Age calculation", the sentence "Ages of survivors were calculated by the duration of interview date and birthday." is unclear. Do you mean that the ages of survivors what calculated by difference between the date of (first?) interview and the date of birth?*

***Response:*** Thank you for this valuable comment. We acknowledge that the previous wording may have caused ambiguity. We have revised the sentence to make it clearer: “Age of survivors were calculated as the difference between the first interview date and the date of birth.” (Page 6, Line 10-11)

***Comment 2:*** *In the section "Individual-level determinants", there is a duplicate (when they when they) in the sentence "All the participants when they when they firstly enrolled cohorts, reported whether have any one of doctor-diagnosed diseases: hypertension,…"*

***Response:*** Thank you for pointing out this important aspect. We apologize for the typographical error and have corrected it in the revised manuscript.

***Comment 3:*** *On Table 2 (as well as on some Figures) Higher TLE and other values go with "Low" Political Stability and Absence of Violence/Terrorism: Estimate and lower values with "High" Political Stability and Absence of Violence/Terrorism: Estimate. Shouldn't it be the other way around?*

***Response:*** Thank you for this insightful comment. Political Stability and Absence of Violence/Terrorism measures perceptions of the likelihood of political instability and/or politically-motivated violence, including terrorism. Therefore, “Low” Political Stability and Absence of Violence/Terrorism indicates that citizens perceive a lower likelihood of politically motivated violence (including terrorism), which corresponds to a higher TLE. To avoid potential ambiguity, we have added the definitions of each country-level determinant in the supplementary materials (**Supplementary 3**).

**Response to Reviewer #4:**

***Comment 1:*** *Introduction "as individuals aging" should probably state "as individuals age"*

***Response:*** Thank you for this valuable comment. We have revised the Introduction section.

***Comment 2:*** *"Previous studies on HWLE have established it" would be better as "Previous studies have established HWLE as a population indicator"*

***Response:*** Thank you for this valuable comment. We have revised the text according to your suggestion. (Page 4, Line 10)

***Comment 3:*** *Methods: "of the participants by waves" should state "of the number of participants by waves"*

***Response:*** Thank you for this valuable comment. We have revised the text according to your suggestion. (Page 9, Line 18-19)

***Comment 4:*** *In the sentence starting with "Using this harmonized data, we designed…" the reviewer would recommend to divide the sentence into two, starting the second one with "among them…"*

***Response:*** Thank you for this valuable comment. We have revised the text according to your suggestion. (Page 9, Line 15)

***Comment 5:*** *Do the authors really mean, that the remaining cases had all data for all determinants, including chronic diseases? It would be unusual that only 5% of the baseline dataset had to be excluded due to missings on any of the determinants. The authors should check if some of the six survey datasets included imputation of missing data, e.g. for the disease indicators.*

***Response:*** Thank you for your important suggestion. We double-checked the inclusion/exclusion workflow independently by two team members and confirmed there was no error. None of the six datasets applied imputation for missing data; all analyses are based on complete observed values.

Although it may seem unusual that only approximately 5% of baseline cases were excluded for missing any individual-level determinant, this relatively low rate can be explained by the following factors:

1. Our handling of chronic disease determinants. For the seven chronic disease indicators, we used each participant’s status at their first entry into the cohort (baseline). We did not require complete information across multiple waves; therefore, baseline missingness for these determinants was limited.

2. Order of exclusions. We applied exclusions sequentially:(1) excluded participants younger than 50 years;(2) excluded those interviewed in only one wave;(3) excluded those with missing age or birth date;(4) finally, excluded participants with missing individual-level determinants. This sequence substantially reduced the denominator before the final step, which helps explain why the proportion excluded specifically for “determinant missingness” is relatively small. For example, in the original HRS population (n=42,233), 6105 participants miss individual-level determinants, accounting for 14.4% of the total sample.

***Comment 6:*** *Outcomes: the brackets at the end of the section, e.g. "health and working: HW" should be an adjective "healthY and working" for all the descriptions of the states.*

***Response:*** Thank you for this valuable comment. We have carefully checked both the main manuscript and the supplementary materials and made the corresponding revisions accordingly. “TLE: total life expectancy; HWLE: healthy and working life expectancy; HnWLE: life expectancy on healthy and not working status; nHWLE: life expectancy on not healthy and working status; nHnWLE: life expectancy on not healthy and not working status.”

***Comment 7***: *Age Calculation: the sentence beginning with "ages of survivors"- first, the word ages means a completely different construct, please use "age". Second: the authors mean surely the difference between the year of the interview and the birth year. It is very unlikely that the respondents delivered their birth date in all cohorts (data protection laws).*

***Response:*** Thank you for this crucial comment. You are right that some respondents did not deliver their birth date. Age is a required variable, so participants with missing age or birth date were exclude in this study. According to your reminder, we checked the harmonized data documents and found that the original research teams have deal with missing birth date. Such as, in HRS, the respondent birth date is derived by searching all waves of data and Tracker for a non-missing date. In SHARE, “RABYEAR and RABMONTH are taken from the first non-missing value of birth data.”

***Comment 8:*** *The next sentence: The authors should necessarily add more information regarding the quality of data on death during follow-up. As much as the reviewer knows data in e.g. share are not that reliable. Please explain shortly for each data source how mortality information was assessed since this is the most important variable in the calculation. Such details could be added in the supplement.*

***Response:*** Thank you for this crucial comment. We completely agree with that mortality information is the most important variable in the calculation. From the harmonized data documents, we know mortality information was gained from exit and post-exit interviews. Besides, partial dead participants were missing death date, which were imputed by the imputed methods previous studies used(Ref.9 and Ref. 10). So, the related section was revised as following:

*“Mortality information gained from exit and post-exit interviews, which interview with someone who knew the dead participants well, like their spouse, child, or another person close to them. Partially missing time of death data were extrapolated from the closest interview date of the last wave and known vital status at this interview[9] and were determined as the mid of the time interval between two consecutive interviews[10].”* (Page 6, Line 13-17)

*According to your suggestion, we also added the details on the exit and post-exit interviews for each cohort in* ***Supplementary 2****.*

***Comment 9:*** *country-level determinants: How was the median built - overall or country specific value? Where did the authors include the values of the median itself - in the high or in the low-group? How did the year/wave play role or the calculation of the median?*

***Response:*** Thank you! We apologize to you for missing methods to medians of country-level factors. Because participants were enrolled in the cohort in different time even in the same cohort. The country-level indicator in the year when the individual was first enrolled in the cohort was used; if data for that year were unavailable, the value from the preceding year was used. Then, the median was calculated using data of all countries included in this study.The related section was added in the revised manuscript:

*“Data corresponding to the country-level factors in the year when participants were first enrolled in the cohort were used. If data for that year were unavailable, values from the preceding year were used instead. The median was then calculated based on data from all countries included in this study.”* (Page 7, Line 15-18)

***Comment 10:*** *Sensitivity analyses: The sentence starting "Participants with missing …" should state " have also been included".*

***Response:*** Thank you for this valuable comment. We have revised the text according to your suggestion. (Page 9, Line 4-5)

***Comment 11***: *Figure 1: the figure looks fancy, but far too small to recognize anything. Please find another way how to visualise it. Would it be enough to show HWLE in the main figure and put the other expectancies in the supplement?*

***Response:*** Thank you for your helpful suggestion. Following your advice, we have revised the figure accordingly. **Figure 1** now presents only HWLE, while the other life expectancies are provided in **Supplementary fig 5**.

***Comment 12:*** *Results with country-level determinants, second section: why DTLE and not TLE?*

***Response:*** Thank you for this helpful comment. “DTLE” refers to the difference in total life expectancy, and “DHWLE” refers to the difference in healthy working life expectancy. To facilitate a more intuitive comparison and improve the clarity of presentation, we reported the results as differences (DTLE and DHWLE). Figures 2-4 also display these differences to make the results easier to interpret.

***Comment 13:*** *Discussion, the section regarding chronic diseases: how could stroke be controlled? The authors mean probably controlling for risk factors leading to stroke and chronic lung disease.*

***Response:*** Thank you for your helpful suggestion. We have revised the relevant sentences to improve readability.

***Comment 14:*** *Limitations: please expound more on the measurement errors.*

***Response:*** Thank you for your valuable comment. We have added relevant details in the limitation section of the discussion. These details are as follows: *“Third, SRH-based HWLE estimates are comparable to those derived from measures of limiting long-standing illness or diagnosed chronic conditions[9, 10], but self-reported measures of health and working status are susceptible to measurement error. The definition of work was inclusive and did not differentiate full-time workers from people who work part time. Moreover, work ability limitations are an outcome of poor health. Some individuals may perceive themselves as good health, yet their work ability is limited. This discrepancy could lead to misestimation of HWLE estimates. Fourth, information on doctor-diagnosed diseases was collected at baseline and not updated during follow-up. Changes in disease status over time, including the onset or remission of diseases, may have affected the estimation of transitions. Fifth, the addition of new participants to each cohort at different times may introduce cohort effects, as disease incidence and prevalence change over time, potentially biasing our estimates. Additionally, throughout the life course, individual intrinsic capacity, relevant environmental characteristics, and the interactions between individuals and their environments are key determinants of healthy aging and functional ability[12, 28].”* (Page 16, Line 10-24)

***Comment 15:*** *As already stated above, one important limitation is completely lacking - mortality data in these surveys. The authors should add an extensive explanation how possible biases in mortality assessment of these data could have influenced the estimations of TLE*

***Response:*** Thank you for your comment. Methods to gain mortality information has been added in the revised manuscript and **Supplementary 2**.

***Journal Requirements:***

1) Please amend your detailed Financial Disclosure statement. This is published with the article. It must therefore be completed in full sentences and contain the exact wording you wish to be published.

1. State the initials, alongside each funding source, of each author to receive each grant.

2. State what role the funders took in the study. If the funders had no role in your study, please state: “The funders had no role in study design, data collection and analysis, decision to publish, or preparation of the manuscript.”

3. If any authors received a salary from any of your funders, please state which authors and which funders.

*Response:* Thank you ! Financial Disclosure statement was revised as following:

“GJ is supported by grants from the National Natural Science Foundation of China (82173634), the National Key Research and Development Program of China (2018YFC2002001). YB is supported by grants from the National Natural Science Foundation of China (82204166), the Young Talent Program of Huadong Hospital (QMRC2201), and the Shanghai Clinical Research Center for Aging and Medicine (19MC1910500). The Funder had no role in the design and conduct of the study; collection, management, analysis, and interpretation of the data; preparation, review, or approval of the manuscript; and decision to submit the manuscript for publication.” (Page 18, Line 8-16)

2) Please insert an Ethics Statement at the beginning of your Methods section, under a subheading 'Ethics Statement'. It must include:

1. The name(s) of the Institutional Review Board(s) or Ethics Committee(s)

2. The approval number(s), or a statement that approval was granted by the named board(s)

3. (for human participants/donors) - A statement that formal consent was obtained (must state whether verbal/written) OR the reason consent was not obtained (e.g. anonymity). NOTE: If child participants, the statement must declare that formal consent was obtained from the parent/guardian.]

***Response:*** Thank you for your comment. We have made the revisions according to your requirement. “This study was approved by the Ethics Committee of the School of Public Health, Fudan University (approval number: IRB#2021-TYSQ-03-108).” (Page 9, Line 9-12)

3) Please ensure that the Title in your manuscript file and the Title provided in your online submission form are the same.

***Response:*** *Thank you! We have checked and keep they are same.*

4) Please upload the main figures as separate figure files in .tif or .eps format. For more information about figure files please see our guidelines:

https://journals.plos.org/plosmedicine/s/figures

https://journals.plos.org/plosmedicine/s/figures#loc-file-requirements

***Response:*** *All figures revised as .tif format, and uploaed separately. Thank you again.*

5) We have noticed that you have uploaded Supporting Information files, but you have not included a list of legends. Please add a full list of legends for your Supporting Information files after the references list.

***Response:*** A list of legends was added after the references list according to your suggestion(Page 21, Line 1-16).

“Legends of Supporting Information files

Supplementary 1. Cohort profiles used in this study

Supplementary 2. Methods to gain mortality information

Supplementary 3. Measures of individual-level and country-level factors of HWLE

Supplementary 4. Methods of HWLE calculation

Supplementary fig 1. Flowchart of participants

Supplementary fig 2. Hypothetical transitions of healthy and working status

Supplementary fig 3. Life expectancy in different states at age 50 by starting state

Supplementary fig 4. Results of sensitivity analyses

Supplementary fig 5. Life expectancy in different states across countries

Supplementary table 1. Number of individuals in each wave across six databases

Supplementary table 2. Sample characteristics across six databases

Supplementary table 3. Life expectancy in different states at age 50 by starting state

Supplementary table 4. Life expectancy in different states across countries

Supplementary table 5. Results of sensitivity analyses by including all missing data, sampling randomly, using different interpolations and health definitions”

6) Some material included in your submission may be copyrighted. According to PLOS’s copyright policy, authors who use figures or other material (e.g., graphics, clipart, maps) from another author or copyright holder must demonstrate or obtain permission to publish this material under the Creative Commons Attribution 4.0 International (CC BY 4.0) License used by PLOS journals. Please closely review the details of PLOS’s copyright requirements here: PLOS Licenses and Copyright. If you need to request permissions from a copyright holder, you may use PLOS's Copyright Content Permission form.

***Response:*** thank you for your suggestion! The map in was created in R using the packages “ggplot2” and “maps”. According to the official documentation of the “maps” package (https://cran.r-project.org/web/packages/maps/refman/maps.html), the world map data used by this package are derived from the Natural Earth dataset (https://www.naturalearthdata.com/).

The Natural Earth data are explicitly stated to be in the public domain, as described in their terms of use (https://www.naturalearthdata.com/about/terms-of-use/).

Therefore, the figure is compatible with the CC BY 4.0 license used by PLOS journals. Following the examples in previously published articles in your journal, I have also added the statement “The maps were drawn using the R package “maps,” which was based on the data from the Natural Earth project.” in the legend of Figure 1 and Supplementary fig 5.
