# Supplementary material for: Country- and individual-level factors associated with healthy working life expectancy: A multinational longitudinal study
Source: PLoS Med. 2026 Sep 10;23(9):e1004676. doi: 10.1371/journal.pmed.1004676 (PMC13561363; doi:10.1371/journal.pmed.1004676)
Supplement: Supplementary file 11 [file pmed.1004676.s012.docx]

Responses to Reviewers’ Comments for Manuscript PMEDICINE-D-25-02153R1

**Country- and individual-level factors associated with healthy working life expectancy: a multinational longitudinal study**

Addressed Comments for Publication to

PLOS Medicine

by

Jingjie Zhu, Bo Ye, Junling Gao

Dear Editors and Reviewers,

We sincerely appreciate the opportunity to revise our manuscript entitled “Country- and individual-level factors associated with healthy working life expectancy: a multinational longitudinal study”, and we are deeply grateful to the reviewers for their insightful and constructive comments. These comments have been extremely valuable in helping us improve the manuscript and refine our study. We have carefully considered each suggestion and made every effort to address all comments clearly and thoroughly in the revised version. We hope that the revisions have satisfactorily addressed the concerns raised and have improved the quality of the manuscript.

Thank you again for your time, consideration, and the opportunity to revise our work.

**Comment 1:**

Estimate validity (Comments 14 & 19): The Switzerland (+~6 yrs) and Austria (+~5 yrs) TLE overestimation was acknowledged as a limitation and the framing shifted to "relative associations," but the bias was not quantified across countries or shown to be non-differential—while absolute HWLE values are still compared country-by-country throughout. Reviewer 2's actual question (is the bias uniform or differential?) is unanswered.

**Response:**

We thank the reviewer for highlighting this important concern. We agree that the potential overestimation of TLE in Switzerland and Austria raises the possibility of differential bias across countries. Unfortunately, we are unable to thoroughly quantitatively estimate the magnitude of such bias because comparable external validation data are unavailable for all participating countries. Therefore, we have revised the manuscript to explicitly acknowledge that the uniformity of this potential bias cannot be assumed and that differential bias cannot be excluded. We have also moderated statements involving absolute cross-country comparisons as following:

*“As shown in Supplementary table 6, the estimated life expectancies varied substantially across countries, with relatively high estimates observed in Switzerland, Sweden, and Denmark. However, given potential differences in cohort representativeness and mortality ascertainment across countries, these absolute estimates should be interpreted cautiously.”* (Page 13, Line 16-19)

We also clarified that our findings should mainly be interpreted in terms of associations between country-level factors and HWLE rather than precise rankings of countries based on absolute HWLE estimates.

*“Finally, we acknowledge that the estimated TLE and HWLE for some countries, particularly Switzerland and Austria, were higher than estimates from official national statistics. This discrepancy suggests that cohort-based estimates may be subject to country-specific biases arising from differences in sampling frameworks, follow-up procedures, mortality ascertainment, and population representativeness. Importantly, we cannot determine whether the magnitude of this potential bias is uniform across countries. Therefore, differential bias cannot be excluded, and comparisons of absolute HWLE values between countries should be interpreted cautiously. Our findings should primarily be interpreted as describing associations between individual- and country-level factors and HWLE within the study framework rather than providing definitive rankings of countries according to absolute HWLE.”* (Page 22, Line 8-18)

**Comment 2:**

Novelty/depth (Comments 6, 7, 8, 21, 22): The aim was reframed around governance metrics, but the core finding (richer/better-governed countries have higher HWLE) is unchanged, and the specific deeper analysis requested — health conditions by country, workplace support, HnW/nHW/nHnW by country (Comment 22) — was not performed.

**Response:**

Thank you for highlighting these important concerns regarding the depth of the analyses. Following your suggestion, we undertook additional analyses and substantially expanded the Results and Discussion in the following three respects.

**1. Workplace support**

We really appreciate this valuable suggestion. To provide an empirical assessment of the employment environment, we searched the World Bank Data360 database and added four country-level employment-related indicators: flexible working arrangements, labour force participation, equal workforce opportunities, and hiring and firing practices. We re-estimated the country-level analyses after classifying participants into high and low groups for each indicator. The results showed that higher levels of all four employment-related indicators were consistently associated with longer HWLE and nHWLE and shorter nHnWLE. We have added these indicators to Supplementary 3, Table 2 and Figure 3, and incorporated the corresponding findings into the Results, Discussion. (Page 14 Line 15- Page 15 Line 5; Page 18 Line 9-22)

**2. Cross-country differences in HnWLE, nHWLE, and nHnWLE**

Thank you for this valuable suggestion. Country-specific estimates of HnWLE, nHWLE, and nHnWLE are presented in Supplementary Table 6. We agree that these three expectancy measures provide important information beyond HWLE alone and may reflect different health–employment trajectories across countries. For example, a relatively long HnWLE may indicate earlier labour-force exit, longer survival after retirement, or stronger financial protection outside paid employment. In response to this comment, we have expanded the Discussion to interpret these cross-country differences and their possible implications.

“*Furthermore, cross-country differences were evident not only in HWLE but also in HnWLE, nHWLE, and nHnWLE, suggesting that similar levels of TLE or HWLE may arise from substantially different health–employment trajectories. A relatively long HnWLE may reflect earlier labour-force exit, longer survival after retirement, or stronger financial protection outside paid employment. By contrast, a longer nHWLE indicates that individuals spend a considerable period working while in poor health. This pattern may reflect employment arrangements that enable people with health limitations to remain economically active, but it may also indicate financial necessity, inadequate retirement protection, or presenteeism. A relatively long nHnWLE represents the combined burden of poor health and labour-force non-participation. These differences may be shaped by chronic disease burden, retirement and disability systems, pension adequacy, labour-market conditions, and workplace support. Understanding these country-specific patterns can provide deeper insight into how national policies and institutional contexts influence the distribution of later life across different health and employment states. Accordingly, HWLE should be interpreted alongside HnWLE, nHWLE, and nHnWLE rather than as an isolated indicator of population health and employment.*” (Page 19 Line 16- Page 20 Line 7)

**3. Country-specific analysis of health conditions**

We attempted to estimate TLE, HWLE, HnWLE, nHWLE, and nHnWLE separately by health condition within each country. However, these stratified multistate models could not be estimated reliably because several diseases had a relatively low prevalence within individual countries, resulting in sparse numbers of observations and state transitions after stratification. For example, only 139 participants in Greece had experienced stroke, 107 participants in Lithuania had chronic lung disease, and 94 participants in Romania had heart problems. Further stratification by both country and disease status resulted in model non-convergence or unstable life-expectancy estimates for a number of countries. We therefore did not report potentially unreliable country-specific disease estimates. We have explained this analytical limitation in the revised Discussion.

*“Although this study pooled data from 28 countries to identify overall associations, the study design and the limited numbers of disease cases and state transitions in some countries restricted our ability to conduct detailed country-specific analyses of the relationship between individual health conditions and HWLE. Larger country-specific samples are needed to determine whether the associations between chronic diseases and working-life expectancy vary across countries. Such analyses may clarify how differences in healthcare systems, chronic disease management, public health interventions, and workplace accommodations modify the impact of chronic conditions on the ability to remain healthy and employed.”* (Page 19, Line 8-15)
