# Supplementary material for: Country- and individual-level factors associated with healthy working life expectancy: A multinational longitudinal study
Source: PLoS Med. 2026 Sep 10;23(9):e1004676. doi: 10.1371/journal.pmed.1004676 (PMC13561363; doi:10.1371/journal.pmed.1004676)
Supplement: Supplementary file 12 [file pmed.1004676.s013.docx]

Responses to Editors’ Comments for Manuscript PMEDICINE-D-25-02153R1

**Country- and individual-level factors associated with healthy working life expectancy: a multinational longitudinal study**

Addressed Comments for Publication to

PLOS Medicine

by

Jingjie Zhu, Bo Ye, Junling Gao

Dear Editors,

We sincerely appreciate the opportunity to revise our manuscript entitled “Country- and individual-level factors associated with healthy working life expectancy: a multinational longitudinal study”. In accordance with the suggestions and the journal’s requirements, we have carefully revised the manuscript and addressed the requested editorial and reporting issues. Thank you again for your time, consideration, and the opportunity to revise our work.

**Comment 1:**

* The Data Availability Statement (DAS) requires revision. PLOS defines the “minimal data set” to consist of the data set used to reach the conclusions drawn in the manuscript with related metadata and methods, and any additional data required to replicate the reported study findings in their entirety. Authors do not need to submit their entire data set, or the raw data collected during an investigation. Please submit the following data:

The values behind the means, standard deviations and other measures reported;

The values used to build graphs;

The points extracted from images for analysis.

**Response:** All data underlying the reported results have been provided in the Supporting Information. The data used to generate Figures 1 and 2 are available in Table 1, while the data underlying Figure 3 are provided in Table 2. Data supporting the remaining reported results are provided in Supplementary Tables 2–7.

**Comment 2:**

* Please specify whether informed consent was obtained. If informed consent was not required or waived please explain whyt. Please ensure that the research complies with the PLOS policy in full: <https://journals.plos.org/plosmedicine/s/human-subjects-research#loc-patient-privacy-and-informed-consent-for-publication>

**Response:** We have verified this information in the Ethics Statement section of the manuscript.

*“This study was approved by the Ethics Committee of the School of Public Health, Fudan University (approval number: IRB#2021-TYSQ-03-108). ”* (Page 11, Line 17-19)

**Comment 3:**

Please define all elements of box plots in the figure caption - center line, box limits and whiskers.

**Response:** This comment is not applicable, as the manuscript does not include any box plots.

**Comment 4:**

Please provide titles and legends for all figures and tables (including those in Supporting Information files). Please define all acronyms used in each figure or table in its corresponding legend.

**Response:** We have carefully checked all figures and tables, including those in the Supporting Information, and confirmed that each includes an appropriate title and legend and that all acronyms are defined in the corresponding legend.

e.g. *“Figure 1: Health expectancies and life expectancy at age 50 according to lifestyle factors. Lifestyle factors include physical activity, smoking, and drinking. Estimates are derived from data on 175,318 individuals, with a mean follow-up of 8.22 years. Tails represent 95% CIs. TLE: total life expectancy; HWLE: healthy and working life expectancy; HnWLE: life expectancy on healthy and not working status; nHWLE: life expectancy on not healthy and working status; nHnWLE: life expectancy on not healthy and not working status”* (Page 33)

**Comment 5:**

Please ensure that where relevant figures include 95% CIs.

**Response:** We have carefully checked the relevant figures and confirmed that 95% CIs are included where applicable.

**Comment 6:**

When a p value is given, please specify the statistical test used to determine it in the legend.

**Response:** This comment is not applicable, as no p values are reported in the figures or their legends.

**Comment 7:**

Please consider avoiding the use of red and green in order to make your figure more accessible.

**Response:** Different colors in the figures are used only to represent the magnitude of differences and are not required for interpretation of the results. We have confirmed that the figures remain readable without relying on red–green distinctions.

**Comment 8:**

Please provide the unadjusted comparisons as well as the adjusted comparisons in all relevant Tables.

**Response:** This comment is not applicable, because TLE and HWLE were estimated using IMaCh based on multistate transition probabilities. In our analysis, no additional covariate adjustment was applied in the IMaCh estimation of life expectancy.

**Comment 9:**

Please specify the variables controlled for in all relevant Tables.

**Response:** This comment is not applicable, because TLE and HWLE were estimated using IMaCh based on multistate transition probabilities. In our analysis, no additional covariate adjustment was applied in the IMaCh estimation of life expectancy.

**Comment 10:**

* Abstract: Please include the study design, population and setting, number of participants, years during which the study took place (enrollment and follow up), length of follow up, and main outcome measures.

**Response:** We have included the study design, study population and setting, number of participants, and main outcome measures in the Abstract. The enrollment periods and duration of follow-up varied across the participating cohorts and countries; therefore, a single enrollment period or follow-up duration was not applicable and was not reported in the Abstract. Detailed information on the study periods and follow-up for each cohort is provided in the Methods and Supporting Information.

**Comment 11:**

* Please ensure that the study is reported according to the STROBE (or appropriate STOBE extension) guideline (available from: https://www.equator-network.org/reporting-guidelines/strobe) and include the completed STROBE (or STROBE extension) checklist as Supporting Information. Please add the following statement, or similar, to the Methods: "This study is reported as per the Strengthening the Reporting of Observational Studies in Epidemiology (STROBE) guideline (S1 Checklist)." When completing the checklist, please use section and paragraph numbers, rather than page numbers.

**Response:**

We have added the completed S1 Checklist as Supporting Information and added the following statement to the Methods section. *“This study adheres to the Strengthening the Reporting of Observational Studies in Epidemiology (STROBE) guidelines (S1 Checklist) and the Guidelines for Accurate and Transparent Health Estimates Reporting (GATHER) statement (S2 Checklist).”* (Page 7, Line 7-9)

**Comment 12:**

* FOR POPULATION HEALTH/REGISTRY STUDIES: Please ensure that the study is reported according to the RECORD guideline (available from https://www.record-statement.org) and include the completed checklist as Supporting Information. Please add the following statement, or similar, to the Methods: "This study is reported as per the Reporting of Studies Conducted using Observational Routinely-Collected Data (RECORD) guideline (S1 Checklist)." When completing the checklist, please use section and paragraph numbers, rather than page numbers.

**Response:** The RECORD guideline is not applicable to this study because the analyses were based on harmonized longitudinal survey data rather than routinely collected health, administrative, electronic medical record, or registry data.

**Comment 13:**

* FOR POPULATION HEALTH ESTIMATES: Please ensure that the study is reported according to the GATHER statement (available from https://www.equator-network.org/reporting-guidelines/gather-statement) and include the completed checklist as Supporting Information. Please add the following statement, or similar, to the Methods: "This study is reported as per the Guidelines for Accurate and Transparent Health Estimates Reporting (GATHER) statement (S1 Checklist)." When completing the checklist, please use section and paragraph numbers, rather than page numbers.

**Response:** We have added the completed S2 Checklist as Supporting Information and added the following statement to the Methods section. *“This study adheres to the Strengthening the Reporting of Observational Studies in Epidemiology (STROBE) guidelines (S1 Checklist) and the Guidelines for Accurate and Transparent Health Estimates Reporting (GATHER) statement (S2 Checklist).”* (Page 7, Line 7-9)

**Comment 14:**

* FOR MEDIATION ANALYSES: We recommend that the study is reported according to the AGReMA statement (https://agrema-statement.org/#:~:text=AGReMA%20is%20an%20evidence%2D%20and,randomised%20trials%20and%20observational%20studies) and include the completed checklist as Supporting Information. Please add the following statement, or similar, to the Methods: "This study is reported as per the Guideline for Reporting Mediation Analyses (AGReMA) statement (S1 Checklist)." When completing the checklist, please use section and paragraph numbers, rather than page numbers.

**Response:** This comment is not applicable as the article did not include mediation analysis.

**Comment 15:**

* For all observational studies, in the manuscript text, please indicate: (1) the specific hypotheses you intended to test, (2) the analytical methods by which you planned to test them, (3) the analyses you actually performed, and (4) when reported analyses differ from those that were planned, transparent explanations for differences that affect the reliability of the study's results. If a reported analysis was performed based on an interesting but unanticipated pattern in the data, please be clear that the analysis was data driven.

**Response:** We have clarified in the manuscript the study objectives and the analytical methods used to address them. The main study objectives and analytical approach were determined before the primary analyses were conducted. The analyses reported in the manuscript were consistent with this planned approach, with no additional data-driven analyses or substantive deviations from the planned analytical methods. *“No formally registered prospective protocol or analysis plan was available. The study objectives and analytical approach were specified before the primary analyses were conducted, and no changes to the analytical approach were made based on data-driven findings or during peer review.”* (Page 10, Line 21-24)

**Comment 16:**

* Did your study have a prospective protocol or analysis plan? Please state this (either way) early in the Methods section.

a) If a prospective analysis plan (from your funding proposal, IRB or other ethics committee submission, study protocol, or other planning document written before analyzing the data) was used in designing the study, please include the relevant prospectively written document with your revised manuscript as a Supporting Information file to be published alongside your study, and cite it in the Methods section. A legend for this file should be included at the end of your manuscript.

b) If no such document exists, please make sure that the Methods section transparently describes when analyses were planned, and when/why any data-driven changes to analyses took place.

c) In either case, changes in the analysis-- including those made in response to peer review comments-- should be identified as such in the Methods section of the paper, with rationale.

**Response:** This study did not have a formally registered prospective protocol or analysis plan. We have clarified this in the Methods section. The main study objectives and analytical approach were determined before the primary analyses were conducted, and the analytical methods were not changed during the peer review process.

*“No formally registered prospective protocol or analysis plan was available. The study objectives and analytical approach were specified before the primary analyses were conducted, and no changes to the analytical approach were made based on data-driven findings or during peer review.”* (Page 10, Line 21-24)

**Comment 17:**

* Your study is observational and therefore causality cannot be inferred. Please remove language that implies causality and refer to associations instead.

**Response:** We have carefully reviewed the manuscript and revised any wording that could imply causality. The findings are now consistently described in terms of associations.
