# Supplementary material for: Country- and individual-level factors associated with healthy working life expectancy: A multinational longitudinal study
Source: PLoS Med. 2026 Sep 10;23(9):e1004676. doi: 10.1371/journal.pmed.1004676 (PMC13561363; doi:10.1371/journal.pmed.1004676)
Supplement: Peer review history

# Editor report: Decision Letter 0

**DOI:** 10.1371/journal.pmed.1004676.r001

**Authors:** Andreia Cunha

24 Jun 2025

Dear Dr Gao,

Thank you for submitting your manuscript entitled "Country- and individual-level determinants of healthy working life expectancy: a multinational longitudinal study" for consideration by PLOS Medicine.

Your manuscript has now been evaluated by the PLOS Medicine editorial staff and I am writing to let you know that we would like to send your submission out for external peer review.

However, before we can send your manuscript to reviewers, we need you to complete your submission by providing the metadata that is required for full assessment. To this end, please login to Editorial Manager where you will find the paper in the 'Submissions Needing Revisions' folder on your homepage. Please click 'Revise Submission' from the Action Links and complete all additional questions in the submission questionnaire.

For clinical studies, please upload a copy of your trial study protocol as a supporting information file. The study protocol should be the version submitted for approval to the institutional review board or ethics committee, should include any amendments to the study protocol, as well as the date of their approval by the institutional review or ethics committee. Please also detail any deviations from the study protocol in the Methods section of your manuscript. The editors will consider the protocol and study conduct prior to a final decision for external review.

Please re-submit your manuscript within two working days, i.e. by Jun 26 2025 11:59PM.

Login to Editorial Manager here: https://www.editorialmanager.com/pmedicine

Once your full submission is complete, your paper will undergo a series of checks in preparation for peer review. Once your manuscript has passed all checks it will be sent out for review.

Feel free to email us at plosmedicine@plos.org if you have any queries relating to your submission.

Kind regards,

Andreia Cunha, PhD

Senior Editor

PLOS Medicine

# Peer review: Decision Letter 1

**DOI:** 10.1371/journal.pmed.1004676.r002

**Authors:** Andreia Cunha

10 Sep 2025

Dear Dr Gao,

Sincere apologies for the delay in getting back to you with a decision, which was due to challenges in securing all the necessary Reviewers. Many thanks for submitting your manuscript "Country- and individual-level determinants of healthy working life expectancy: a multinational longitudinal study" (PMEDICINE-D-25-02153R1) to PLOS Medicine. The paper has been reviewed by subject experts and a statistician; their comments are included below and can also be accessed here: [LINK]

As you will see, the reviewers find your work of considerable interest but raise some methodological concerns that need to be resolved. After discussing the paper with the editorial team and an academic editor with relevant expertise, I'm pleased to invite you to revise the paper in response to the reviewers' comments. We plan to send the revised paper to some or all of the original reviewers, and we cannot provide any guarantees at this stage regarding publication.

In addition to these revisions, you may need to complete some formatting changes, which you will receive in a follow up email. A member of our team will be in touch with a set of requests shortly. If you do not receive a separate email within a few days, please assume that checks have been completed, and no additional changes are required.

When you upload your revision, please include a point-by-point response that addresses all of the reviewer and editorial points, indicating the changes made in the manuscript and either an excerpt of the revised text or the location (eg: page and line number) where each change can be found. Please also be sure to check the general editorial comments at the end of this letter and include these in your point-by-point response. When you resubmit your paper, please include a clean version of the paper as the main article file and a version with changes tracked as a marked-up manuscript. It may also be helpful to check the guidelines for revised papers at http://journals.plos.org/plosmedicine/s/revising-your-manuscript for any that apply to your paper.

We ask that you submit your revision by Dec 10 2025 11:59PM. However, if this deadline is not feasible, please contact me by email, and we can discuss a suitable alternative.

Don't hesitate to contact me directly with any questions (acunha@plos.org).

Best regards,

Andreia

Andreia Cunha, PhD

Senior editor

PLOS Medicine

acunha@plos.org

-----------------------------------------------------------

Comments from the reviewers:

Reviewer #1: This study integrates multiple international prospective cohort datasets to examine the effects of both country- and individual- specific characteristics on healthy working life expectancy.

Overall, this manuscript is an excellent initial submission that contextualises the research and establishes its importance. It is clearly written, logically presented, and cogently argued. That said, I do have some statistically and methodologically focussed concerns.

Detailed feedback on the manuscript is provided below. My primary focus has been methodological, but I have made a few broader points as well. All items are major unless indicated otherwise.

1.[MINOR] In the abstract the authors say "… chronic diseases, particularly stroke, reduced HWLE by 5·49 years (95%CI:4·94-6·04)". It's not clear to me whether these statistics refer to individuals with any chronic disease or with stroke in particular. Suggest clarifying.

2.[MINOR] While the English in this paper is very good, there are a few instances of unusual wording (e.g., "These individual socioeconomic disparities arise great concerns for policymakers…", "The detail cohorts' information (see Supplementary 1) of the participants by waves…", "duration of interview date") that would benefit from further checking.

3.[MINOR] Suggest the authors very briefly explain what Gini index measures (income inequality?) for those of us who are unfamiliar with it.

4.Otherwise, the Introduction is excellent. Clear, succinct, and comprehensive.

5.I suggest the authors ensure only study methods are included in the Methods section - any results (including numbers of patients included/excluded and references to main or supplementary figures with patient flow) should be in the Results section.

6.Looking at the "doctor diagnosed condition" at the supplied website (https://g2aging.org/vac/home) I noticed quite a few conditions that could foreseeably impact HWLE (e.g., "Alzheimer's disease and Dementia", "Arthritis", "Parkinson's Disease", "Psychological conditions", and others) were available on the website but not included in the analyses. Suggest the authors consider inclusion or justify their reasoning for only analysing a subset.

7.[MINOR] The Statistical Analysis section of the Methods is well-presented, and I appreciate the additional information provided in the Supplement. I am not sure of the value of including the 95%CI formula given a reference is provided (formulae can scare some readers off), however I will leave this decision to the authors.

8.The authors could consider explicitly noting any important assumptions underpinning the discrete-time Markov multi-state model-based methods used. Also, being clear on whether transition probabilities were assumed constant over time or whether they were allowed to vary by age.

9.Another concern I have is it's very difficult to keep track of all the explanatory variables across individual and country levels. I would encourage the authors to include a table or figure (supplementary is fine) that lists all the variables in one place and clearly shows when they are first recorded and if/when they are updated during follow-up.

10.With respect to dichotomising various continuous variables (e.g., country-level GDP), while this historically has been common practice, recent methodological guidance discourages this ( https://doi.org/10.1136/bmj.332.7549.1080 ). I suggest the authors consider modelling continuous predictors as continuous variables.

11.Similarly, I found the Sensitivity Analysis section unclear. I suggest the authors make much clearer what each of the seven sensitivity analysis is changing as compared to the main analysis. This could be done textually in the current section or in a supplementary table. It should also be very clear that results are presented for each of the specified sensitivity analyses.

12.Remember to make sure each table and figure is comprehensible without referring to other parts of the manuscript, particularly when using abbreviations. The authors have done this for the first two main tables but not for all tables and figures in the main manuscript and supplementary materials. It is particularly important for observational studies like this one where tables and figures in the supplement are referred to quite a lot.

13.Suggest the authors ensure that rows and columns of tables are appropriately labelled - this is especially important for tables like Supplementary Table 3 with similar variables on both axes.

14.Suggest avoiding the word "significant" unless specifically referring to statistical significance e.g., "HWLE showed a significant upward trend with increasing educational attainment…", "Although no significant difference in TLE was observed between drinkers… ". If the authors mean statistical significance, then appropriate statistics should be presented. This is problematic in the Sensitivity Analysis results where the authors claim "…no statistically significant discrepancies…" without presenting any statistics.

15.Relatedly, all comparisons presented are descriptive i.e., results are presented alongside each other with no quantification or analysis of the difference e.g., "Women had a higher TLE (35·29 years [95%CI: 35·16 to 35·42]) than men (31·72 years [95%CI: 31·58 to 31·87]).". Was this a deliberate choice by the authors or a limitation of the methods used? Either is ok, I just think it might be worth being clear on this in the methods.

16.Is not clear to me whether "doctor-diagnosed diseases" are updated after enrolment. My earlier comment about making this clearer across all explanatory variables will resolve this but if the diseases are not updated after enrolment, I suggest the authors include this as a limitation because these obviously emerge over a lifetime.

17.Otherwise, the Discussion is excellent - well done!

Reviewer #2: Thank you for preparing this manuscript. I enjoyed reading it. Please consider the following suggestions:

Introduction

First sentence - please revise for clarity.

Last sentence of first paragraph - please elaborate on why gender, educational attainment, regional governance can guide policymakers. Please also reference why a flexible and equitable reitrement system should be a goal - I am not sure what this would look like or is feasible.

Second paragraph as last point please outline what equitable pension policies are.

Last paragraph of introduction: Is the sample globally representative? Please provide evidence, using data.

Please revise the second last sentence - there are a large number of factors associated with lower HWLE. Please note that these are associations not determinants (the analysis does not indicate causation).

Please justify why the comparison between countries informs strategies - unclear how it will do this at this point.

Methods

The Global Ageing data is a strength for harmonising data from different countries. The introduction claims that this is a globally representative population (of adults aged 50 years and over) - provide the data to support this - does data from US, England, Europe, Korea, Mexico and China represent the global population? Please also consider how you will support that each of these populations is representative of the country; data in supplementary table 4 questions this.

Outcome - the intro indicates that HWLE should be measured with reference to functional capacity, but the outcome is based on self-reported health. Will this reduce the strength of this work as it does not refer to functional/work capacity? How will this link with health and work policy.

Individual-level determinants - please outline why these determinants were selected.

Statistical analysis - The description of the analysis needs to be clearer and transparent. It is unclear how variables are being included in the analysis; this needs to be clearly stated. For individual factors; it needs to be clear that HWLE is calculated for each category of each variable. It is unclear how HWLE by country is estimated (stratified or for each country); the methods section should clearly state this.

Please clarify in the methods which population is stratified for health condition and lifestyle factors.

Please state the time period for transitions.

Please state why the particular sensitivity analysis is being performed; previously references indicate that self-rated health is similar to functional loss. Please state why you selected a more severe form of functional loss (similar to functional loss in the oldest old!) for a sensitivity analysis?

Results

Supplementary table 2 - suggests that this is a less deprived population than the global average - please comment on this

The average total life expectancy seems high - 33.56 years worldwide! All of the life expectancies for the European countries (using SHARE data) are 5 years higher than those reported by government agencies (https://www.europeinnumbers.com/rankings/life_expectancy_at_fifty/). This data reports that TLE for Switzerland is 40.46 years (that is men and women on average from age 50 live until they are 90.46 years; this suggest that a large proportion of people live beyond 90 years). Is this true? If TLE if being over-estimated are all other estimates too high?

Linked to this current smokers can still expect to live on average to 78 worldwide? Please check the TLE estimates.

Why provide estimates for different states at age 50? Would policy interventions not target these states in the overall population?

Supplementary data table 4 - Please advise on the interpretation of estimates for countries with small sample sizes (e.g. Latvia). The confidence intervals for TLE span 18 years, HWLE span 6 years. I am presuming that small numbers made some transitions between the five states (hence the large CI). Is the small number an issue for representativeness for that country?

Page 10 - should it be that physical inactivity is associated with lower TLE?

Discussion

Please expand on why this data will raise concerns for delaying retirement. It is not outlined why HWLE is important as an indicator. Can it ever be equivalent to retirement age?

Please expand on why the indicator is important - it is unclear.

The relation between TLE, HWLE and indicators of countries economies is not novel (that is, richer countries with greater governance have healthier populations and better job opportunities). Please outline why this analysis is novel.

Low and middle income countries is discussed; what LMIC are included in this analysis?

HWLE for HNW, NHW and NHNW in highly dependent on employment opportunities. Please expand on this.

Comparison of results with other studies; as outlined above, please comment on the high TLE and the impact on HWLE. Are these estimates of HWLE too high?

Please discuss HWLE estimates for some conditions. HWLE for someone with a stroke seems high across the countries covered.

Please discuss limitations in the data collected. Does employment mean it includes working 1 hour or more? How will this bias results?

The introduction highlights the importance of function as a measure of health but self-reported health is used. Can people have good self-reported health but have limited work capacity?; work capacity being crucial for policy development.

Please comment on the limitation of representativeness of the overall sample and each country.

As above, I disagree with the statement in the last paragraph (page 14) that TLE is higher because of earlier retirement ages; this must be an issue linked to selection of participants or how calculated. Non-representative samples for countries may explain this. Please comment on the representativeness of the data for countries.

Limitations - please expand on what you mean by "confounding cross-country comparisons"? What is the direction of the measurement error for self-reported information? What data supports cohort effects? Please expand on why two variables could not be included in the analysis

Please give more detail in the novel guidance this has for policy. Is government policy n the countries included not already aiming to reduce smoking , alcohol use, increase activity etc and better manage economies and health?

Reviewer #3:

The research question is very clear. It is original. It is particularly relevant in the current context of an accelerated aging population. The literature review on Health Working Life Expectancy (HWLE) is correct. The authors benefit from an internet portal (the Gateway to Global Aging Data) that provides all the data necessary for their study, from comparable surveys on health and work in 33 countries. The choice of individual variables retained is judicious and justified by the literature. The same is true for the socioeconomic variables retained at the country level, provided by the World Bank. The multistate calculation method (IMaCh) is well adapted to the research question and the data. The results are clear and well presented. The discussion is of good quality. In short, this study demonstrates the value of cross-referencing individual variables with macroeconomic indicators for each country. The authors emphasize at the end of the discussion: "Higher national economic status and governance quality are associated with longer TLE (Total Life Expectancy) and HWLE (Healthy Working Life Expectancy). In contrast, unhealthy behaviors and chronic diseases are linked to lower overall life expectancy and healthy working years."

Typos and small errors

In the section "Age calculation", the sentence "Ages of survivors were calculated by the duration of interview date and birthday." is unclear. Do you mean that the ages of survivors what calculated by difference between the date of (first?) interview and the date of birth?

In the section "Individual-level determinants", there is a duplicate (when they when they) in the sentence "All the participants when they when they firstly enrolled cohorts, reported whether have any one of doctor-diagnosed diseases: hypertension,…"

On Table 2 (as well as on some Figures) Higher TLE and other values go with "Low" Political Stability and Absence of Violence/Terrorism: Estimate and lower values with "High" Political Stability and Absence of Violence/Terrorism: Estimate. Shouldn't it be the other way around?

Reviewer #4: Comments on PLOS article country- and individual-level determinants of HWLE and TLE

The authors have submitted a very extensive analysis on country- and individual-level determinants of healthy working life expectancy using data from six large health and retirement surveys. The idea of the paper is innovative, the analysis is performed in a very thorough way. Still there is need for some clarifying details.

The most important point is lack of detailed information about mortality follow-ups in the six surveys. The authors mention only in one sentence that the death year was used from such mortality follow-ups, but do not report at more detail how mortality data was assessed and do not discuss the implications of possible incompleteness of mortality data in surveys. This information should be added necessarily since death date/year is the basis for the crucial indicator for the calculation of the TLE. Furthermore, the authors should discuss the implications for the calculations.

Some more comments:

1) Introduction "as individuals aging" should probably state "as individuals age"

2) "Previous studies on HWLE have established it" would be better as "Previous studies have established HWLE as a population indicator"

3) Methods: "of the participants by waves" should state "of the number of participants by waves"

4) In the sentence starting with "Using this harmonized data, we designed…" the reviewer would recommend to divide the sentence into two, starting the second one with "among them…"

5) Do the authors really mean, that the remaining cases had all data for all determinants, including chronic diseases? It would be unusual that only 5% of the baseline dataset had to be excluded due to missings on any of the determinants. The authors should check if some of the six survey datasets included imputation of missing data, e.g. for the disease indicators.

6) Outcomes: the brackets at the end of the section, e.g. "health and working: HW" should be an adjective "healthY and working" for all the descriptions of the states

7) Age Calculation: the sentence beginning with "ages of survivors"- first, the word ages means a completely different construct, please use "age". Second: the authors mean surely the difference between the year of the interview and the birth year. It is very unlikely that the respondents delivered their birth date in all cohorts (data protection laws).

8) The next sentence: The authors should necessarily add more information regarding the quality of data on death during follow-up. As much as the reviewer knows data in e.g. share are not that reliable. Please explain shortly for each data source how mortality information was assessed since this is the most important variable in the calculation. Such details could be added in the supplement.

9) country-level determinants: How was the median built - overall or country specific value? Where did the authors include the values of the median itself - in the high or in the low-group? How did the year/wave play role or the calculation of the median?

10) Sensitivity analyses: The sentence starting "Participants with missing …" should state " have also been included".

11) Figure 1: the figure looks fancy, but far too small to recognize anything. Please find another way how to visualise it. Would it be enough to show HWLE in the main figure and put the other expectancies in the supplement?

12) Results with country-level determinants, second section: why DTLE and not TLE?

13) Discussion, the section regarding chronic diseases: how could stroke be controlled? The authors mean probably controlling for risk factors leading to stroke and chronic lung disease.

14) Limitations: please expound more on the measurement errors

15) As already stated above, one important limitation is completely lacking - mortality data in these surveys. The authors should add an extensive explanation how possible biases in mortality assessment of these data could have influenced the estimations of TLE

Overall, it is a very important study. Methodologically it should be improved a bit.

Any attachments provided with reviews can be seen via the following link: [LINK]

--------------------------------------------------------- ---

General editorial requests:

(Note: not all will apply to your paper, but please check each item carefully)

*We ask every co-author listed on the manuscript to fill in a contributing author statement, making sure to declare all competing interests. If any of the co-authors have not filled in the statement, we will remind them to do so when the paper is revised. If all statements are not completed in a timely fashion this could hold up the re-review process. If new competing interests are declared later in the revision process, this may also hold up the submission. Should there be a problem getting one of your co-authors to fill in a statement we will be in contact. Please do not add or remove authors without first discussing this with the handling editor. You can see our competing interests policy here: http://journals.plos.org/plosmedicine/s/competing-interests.

*Please upload any figures associated with your paper as individual TIF or EPS files with 300dpi resolution at resubmission; please read our figure guidelines for more information on our requirements: http://journals.plos.org/plosmedicine/s/figures. While revising your submission, we strongly recommend that you use PLOS's NAAS tool (https://ngplosjournals.pagemajik.ai/artanalysis) to test your figure files. NAAS can convert your figure files to the TIFF file type and meet basic requirements (such as print size, resolution), or provide you with a report on issues that do not meet our requirements and that NAAS cannot fix.

After uploading your figures to PLOS's NAAS tool - https://ngplosjournals.pagemajik.ai/artanalysis, NAAS will process the files provided and display the results in the "Uploaded Files" section of the page as the processing is complete.

If the uploaded figures meet our requirements (or NAAS is able to fix the files to meet our requirements), the figure will be marked as "fixed" above. If NAAS is unable to fix the files, a red "failed" label will appear above.

When NAAS has confirmed that the figure files meet our requirements, please download the file via the download option, and include these NAAS processed figure files when submitting your revised manuscript.

*Please ensure that the paper adheres to the PLOS Data Availability Policy (see http://journals.plos.org/plosmedicine/s/data-availability), which requires that all data underlying the study's findings be provided in a repository or as Supporting Information. For data residing with a third party, authors are required to provide instructions with contact information (web or email address) for obtaining the data. Please note that a study author cannot be the contact person for the data. PLOS journals do not allow statements supported by "data not shown" or "unpublished results." For such statements, authors must provide supporting data or cite public sources that include it.

*We expect all researchers with submissions to PLOS in which author-generated code underpins the findings in the manuscript to make all author-generated code available without restrictions upon publication of the work. In cases where code is central to the manuscript, we may require the code to be made available as a condition of publication. Authors are responsible for ensuring that the code is reusable and well documented. Please make any custom code available, either as part of your data deposition or as a supplementary file. Please add a sentence to your data availability statement regarding any code used in the study, e.g. "The code used in the analysis is available from Github [URL] and archived in Zenodo [DOI link]" Please review our guidelines at https://journals.plos.org/plosmedicine/s/materials-software-and-code-sharing and ensure that your code is shared in a way that follows best practice and facilitates reproducibility and reuse. Because Github depositions can be readily changed or deleted, we encourage you to make a permanent DOI'd copy (e.g. in Zenodo) and provide the URL.

FORMATTING - GENERAL

*Abstract: Please structure your abstract using the PLOS Medicine headings (Background, Methods and Findings, Conclusions). Please combine the Methods and Findings sections into one section.

*At this stage, we ask that you include a short, non-technical Author Summary of your research to make findings accessible to a wide audience that includes both scientists and non-scientists. The Author Summary should immediately follow the Abstract in your revised manuscript. This text is subject to editorial change and should be distinct from the scientific abstract. Ideally each sub-heading should contain 2-3 single sentence, concise bullet points containing the most salient points from your study. In the final bullet point of 'What Do These Findings Mean?', please include the main limitations of the study in non-technical language. Please see our author guidelines for more information: https://journals.plos.org/plosmedicine/s/revising-your-manuscript#loc-author-summary.

*Please express the main results with 95% CIs as well as p values. When reporting p values please report as p<0.001 and where higher as the exact p value p=0.002, for example. Throughout, suggest reporting statistical information as follows to improve clarity for the reader "22% (95% CI [13%,28%]; p</=)". Please be sure to define all numerical values at first use.

*Please include page numbers and line numbers in the manuscript file. Use continuous line numbers (do not restart the numbering on each page).

*Please cite the reference numbers in square brackets. Citations should precede punctuation.

FIGURES AND TABLES

*Please provide titles and legends for all figures and tables (including those in Supporting Information files).

*Please define all abbreviations used in each figure/table (including those in Supporting Information files).

*Please consider avoiding the use of red and green in order to make your figure more accessible to those with color blindness.

SUPPLEMENTARY MATERIAL

*Please note that supplementary material will be posted as supplied by the authors. Therefore, please amend it according to the relevant comments outlined here.

*Please cite your Supporting Information as outlined here: https://journals.plos.org/plosmedicine/s/supporting-information

REFERENCES

*PLOS uses the numbered citation (citation-sequence) method and first six authors, et al.

*Please ensure that journal name abbreviations match those found in the National Center for Biotechnology Information (NCBI) databases (http://www.ncbi.nlm.nih.gov/nlmcatalog/journals), and are appropriately formatted and capitalised.

*Where website addresses are cited, please include the complete URL and specify the date of access (e.g. [accessed: 12/06/2023]).

*Please also see https://journals.plos.org/plosmedicine/s/submission-guidelines#loc-references for further details on reference formatting.

OBSERVATIONAL STUDIES

*Abstract: Please include the study design, population and setting, number of participants, years during which the study took place (enrollment and follow up), length of follow up, and main outcome measures.

*Please ensure that the study is reported according to the STROBE (or appropriate STOBE extension) guideline (available from: https://www.equator-network.org/reporting-guidelines/strobe) and include the completed STROBE (or STROBE extension) checklist as Supporting Information. Please add the following statement, or similar, to the Methods: "This study is reported as per the Strengthening the Reporting of Observational Studies in Epidemiology (STROBE) guideline (S1 Checklist)." When completing the checklist, please use section and paragraph numbers, rather than page numbers.

*For all observational studies, in the manuscript text, please indicate: (1) the specific hypotheses you intended to test, (2) the analytical methods by which you planned to test them, (3) the analyses you actually performed, and (4) when reported analyses differ from those that were planned, transparent explanations for differences that affect the reliability of the study's results. If a reported analysis was performed based on an interesting but unanticipated pattern in the data, please be clear that the analysis was data driven.

*Please state in the Methods section whether the study had a prospective protocol or analysis plan. If a prospective analysis plan (from your funding proposal, IRB or other ethics committee submission, study protocol, or other planning document written before analyzing the data) was used in designing the study, please include the relevant document(s) with your revised manuscript as a Supporting Information file to be published alongside your study and cite it in the Methods section. A legend for this file should be included at the end of your manuscript. If no such document exists, please make sure that the Methods section transparently describes when analyses were planned, and when/why any data-driven changes to analyses took place. Changes in the analysis, including those made in response to peer review comments, should be identified as such in the Methods section of the paper, with rationale.

MODELLING STUDIES

The following list is derived from Geoffrey P Garnett, Simon Cousens, Timothy B Hallett, Richard Steketee, Neff Walker. Mathematical models in the evaluation of health programmes. (2011) Lancet DOI:10.1016/S0140-6736(10)61505-X:

*If pertinent, please provide a diagram that shows the model structure, including how the natural history of the disease is represented, the process and determinants of disease acquisition, and how the putative intervention could affect the system.

*Please provide a complete list of model parameters, including clear and precise descriptions of the meaning of each parameter, together with the values or ranges for each, with justification or the primary source cited and important caveats about the use of these values noted.

*Please provide a clear statement about how the model was fitted to the data, including goodness-of-fit measure, the numerical algorithm used, which parameter varied, constraints imposed on parameter values, and starting conditions.

*For uncertainty analyses, please state the sources of uncertainties quantified and not quantified [can include parameter, data, and model structure].

*Please provide sensitivity analyses to identify which parameter values are most important in the model. Uncertainty estimates seek to derive a range of credible results on the basis of an exploration of the range of reasonable parameter values. The choice of method should be presented and justified.

*Please discuss the scientific rationale for the choice of model structure and identify points where this choice could influence conclusions drawn. Please also describe the strength of the scientific basis underlying the key model assumptions.

# Author comment: Author response to Decision Letter 2

**DOI:** 10.1371/journal.pmed.1004676.r003

9 Nov 2025

Attachment Submitted filename: Response to Reviewer1105.docx

# Peer review: Decision Letter 2

**DOI:** 10.1371/journal.pmed.1004676.r004

**Authors:** Andreia Cunha

16 Jan 2026

Dear Dr Gao,

Many thanks for submitting your revised manuscript "Country- and individual-level factors associated with healthy working life expectancy: a multinational longitudinal study" (PMEDICINE-D-25-02153R2) to PLOS Medicine and apologies for the delay in getting back to you with a decision. The paper has been reviewed by the original subject experts and statistician; their comments are included below and can also be accessed here: [LINK]

As you will see, the reviewers maintain some serious concerns about the data and depth of discussion, in particular Reviewer 2 is concerned with the accuracy of the estimates. After discussing the paper with the editorial team and an academic editor with relevant expertise, I'm pleased to invite you to revise the paper in response to the reviewers' comments. We plan to send the revised paper to some of the original reviewers, and we cannot provide any guarantees at this stage regarding publication. However, please note we will be reluctant to send it back to Reviewers unless the concerns of Reviewer 2 have been fully resolved with a thorough revision.

In addition to these revisions, you may need to complete some formatting changes, which you will receive in a follow up email. A member of our team will be in touch with a set of requests shortly. If you do not receive a separate email within a few days, please assume that checks have been completed, and no additional changes are required.

When you upload your revision, please include a point-by-point response that addresses all of the reviewer and editorial points, indicating the changes made in the manuscript and either an excerpt of the revised text or the location (eg: page and line number) where each change can be found. Please also be sure to check the general editorial comments at the end of this letter and include these in your point-by-point response. When you resubmit your paper, please include a clean version of the paper as the main article file and a version with changes tracked as a marked-up manuscript. It may also be helpful to check the guidelines for revised papers at http://journals.plos.org/plosmedicine/s/revising-your-manuscript for any that apply to your paper.

We ask that you submit your revision by May 01 2026 11:59PM. However, if this deadline is not feasible, please contact me by email, and we can discuss a suitable alternative.

Don't hesitate to contact me directly with any questions (acunha@plos.org).

Best regards,

Andreia

Andreia Cunha, PhD

Senior editor

PLOS Medicine

acunha@plos.org

-----------------------------------------------------------

Comments from the reviewers:

Reviewer #1: I thank the authors for their considered and comprehensive responses to my comments and note the manuscript is reading very well. In all but two cases, I am happy to accept the authors' proposed updates and consider the items finalised. However, I do suggest the authors revisit the two items below (numbers reference my original review items).

Comment 11

Unfortunately, I am still struggling to understand which sensitivity analysis results in the supplementary materials (notated by "Method X") correspond to which description in the Methods. Suggest the authors make this crystal clear by referencing the method number in the text and ensuring each method number 1-7 is covered e.g., "First, to verify whether the results were sensitive to the health definition, health based on activities of daily living was defined as good if there were no difficulties with activities of daily living and poor if difficulty was reported with at least one activity from bathing, dressing, eating, getting in and out of bed, and walking across a room (Method 1)".

Comment 12

The authors have made good progress on this point but there are still cases where there are definitions used in tables or figures that are not defined in each case e.g., "heart problems" in Figure 3, "GDP" in Figure 4, dataset names in Supplementary Figure 1, and both "SRH" and "ADL" in Supplementary Figure 4. Also in Supplementary Table 5 and Supplementary Figure 4, ensuring enough detail is included in the table/figure notes to succinctly but completely define the analysis method used for each "Method" number. Suggest carefully checking each table and figure and ensuring all definitions are covered.

Reviewer #2: Thank you for revising the manuscript.

This manuscript requires further clarity in the introduction and methods and much more depth in the discussion, in my opinion to be publishable. There are lots of broad statements that require depth and justification. This is analysis of comparable data from 28 countries selected because the data was available. The opportunity for meaningful comparisons to direct approaches to increase health and work are not taken. The discussion does not offer new insights for health and work practice or policy development. The methods lack detail to properly scrutinize the cross-national comparison; HWLE being higher in countries with more positive factors is not novel. The calculation of life expectancies in SHARE data is problematic - TLE is so much higher for some countries that is questionable if the HWLE estimates are accurate; I have highlighted the issue with Switzerland previously but these are not the only country that this is questionable. The response to comments does not explain the extent of the difference. It is difficult to compare estimates if some are inaccurate. More could also be done to check the representativeness of SHARE country data as number by country are small (too allow sufficient transition to occur).

Specific suggestions

1.Abstract - I suggest removing average data but focus on the range. The average is dependent on the selection of the countries

2.Intro - first paragraph - "It is crucial to extend working life expectancy in healthy state among older workers." - please clarify what this means and why it is crucial.

3.Intro - Please clarify "Although health status is a major determinant of HWLE for older workers, current evidence suggests that individual-level factors, including gender, education, lifestyle, and chronic conditions—are associated with HWLE." Health is part of the HWLE construct; how can it also be a determinant?

4.Intro - Revise "Fully examining the associations between individual- and country-level factors and HWLE can deepen understanding of the relationships between politics, economy, employment, and health, and guide the development of country-specific measures, such as healthcare investment policies, governance capacity building, and the optimization of employment and retirement". This analysis and manuscript does not do this; please stop overstating what this analysis is doing or explain how your manuscript deepens the understanding between politics, healthcare investment and the optimisation of employment and retirement.

5.Revise: "to identified the individual-level and country-level factors that influencing healthy working lives". The analysis is not causal.

6.The aim is to estimate HWLE in 28 countries but it needs to be stated and elaborated in the discussion why comparing HWLE for 28 countries is useful. Please outline how your analysis explains differences between countries. Should the results and discussion further explore why HWLE differs by health conditions - is this about healthcare provision or as outlined above politics or economy.

7.Please also state the novelty of the country level factors; the factors are a measure of wealth. The results indicate that wealthier countries have higher HWLE which is known. The last paragraph indicates that you would explore factors in more depth but this is not achieved. Please re-frame the aim and purpose of this work.

Methods.

1.The study has selected factors identified in other studies -what is the novelty of looking at these again - please state that the aim is to compare between countries (and check this is done in the results).

2.Please state that the 28 countries were selected on data availability. Please outline for SHARE in the appropriate Table, the sample sizes for each European country. Please add in the discussion that not all samples are generalisable. The methods fails to outline how survey weights were used

3."in order to assess impacts of country-level factors on life expectancy and health expectancies, we firstly calculated life expectancies for participants stratified by each country-level factor, and then calculated the differences and 95%CI of the differences between high and low level of each country-level factor". - please clarify. I am unable to reproduce this method clearly from the write-up. I am presuming that each individual in the dataset from one country has the same value for each factor. All participants are then added together, TLE and HWLE is calculated and the stratified by country? Is this circular? As intro I am not sure what this adds to what we know that richer countries have higher HWLE, perhaps we don't know why this is (is it due to how healthcare is funded or workplace health is managed?) - please state.

4.Sensitivity analyses - As outlined previously why compare self-reported health with ADLs - these are not relevant to work and capture a high level of disability, quite different to health

5."Finally, to examine the results' sensitivity to varied analysis methods, we used shorter interpolations (12-month step and 18-month step) and longer interpolations (36-month step) to construct transition probability models to calculate TLE and health expectancies - state why you have done these analyses.

Results:

1.Please remove the work "globally" the datasets do not provide a global sample.

2.The TLE for Switzerland continues to indicate an issue with the validity of your estimates. The problem appears to be with SHARE data. The estimate is 6 years higher than official estimates - does that mean that all other estimates for Switzerland are around 15% higher than the true value? The methods are overestimating life expectancies for some countries - it means the estimates are difficult to compare because there is a bias which is overestimating the values

3.I don't feel any of the maps add anything to the results- most of it is grey because the countries are not in the analysis.

4.There is very little comparison between countries for the other variables - please consider adding

5.Supplementary Table 1 indicates that the datasets contributed different number of waves and time periods. Did this place a part in estimates if data was collected at different time points. How would this impact on the country wide comparisons?; are confidence intervals important?.

6.Supplementary Table 2 - please stratify SHARE by country - the estimates are by country. As sample size is important please outline the size for each European country included.

7.Supplementary Table 4 - TLE estimates do not align with official estimates https://www.europeinnumbers.com/rankings/life_expectancy_at_fifty/ As outlined in the first review this questions the estimates or all of the HWLE states. The differences in TLE to official estimates are significant (e.g. 5 years for Austria as well as for Switzerland in the last review.

Discussion

1."The results corroborate previous findings from 14 England[9], Australia[11], Europe[18], and China[10], indicating that inequalities in HWLE persist among individuals of varying socioeconomic statuses and extending working life remains a formidable challenge". - outline why the results indicate that it will be a challenge. Was this an opportunity to compare the differences by country between HWLE and state pension provision.

2."Specifically, national health expenditure and GDP per capita emerged as pivotal determinants of longer life expectancy, whereas tax revenue was more closely linked to

maintaining a healthy workforce". This conforms what we know - countries with higher incomes have higher HWLE. Can you take this any further or does the data limit developing depth

3."Our findings, based on self-reported health measures, also 7 show that stroke and chronic lung disease are associated with lower TLE and HWLE, indicating controlling for risk factors leading to stroke and chronic lung disease among adults may benefit to increasing TLE and HWLE." This is a missed opportunity to compare the relationship with health conditions by country. This would provide a more in-depth discussion on why differences occur between countries and hence potential interventions. Whilst prevention of health conditions is important, HWLE would increase with workplace support - would be worth exploring? Would it also be worth discussing differences between countries and health conditions for HnW, nHW and nHnW?

4.Limitation 2 - expand on why small sample sizes for a methodology that focuses on transitions is a problem in addition. With regards generalisability - can you compare country samples with country distributions?

5.Limitation 5 -cohort effects can be examined for.

6."Nevertheless, this study provides valuable insights, including the predictive utility of

7.national economic and governance quality indicators for population health and workforce, as well as the influence of chronic diseases on TLE and HWLE, offering potential guidance for

measures to extend healthy working life" - please explain how the data provides guidance.

8."In summary, a variety of multilevel interventions are warranted to extend the TLE and HWLE." This indicates the potential to outline more research -please expand.

Reviewer #3: Thank you for this revised manuscript.

When you write…

Response: "Thank you for this insightful comment. Political Stability and Absence of Violence/Terrorism measures perceptions of the likelihood of political instability and/or politically-motivated violence, including terrorism. Therefore, "Low" Political Stability and Absence of Violence/Terrorism indicates that citizens perceive a lower likelihood of politically motivated violence (including terrorism), which corresponds to a higher TLE. To avoid potential ambiguity, we have added the definitions of each country-level determinant in the supplementary materials (Supplementary 3)."

to my comment…

Comment 3: "On Table 2 (as well as on some Figures) Higher TLE and other values go with "Low" Political Stability and Absence of Violence/Terrorism: Estimate and lower values with "High" Political Stability and Absence of Violence/Terrorism: Estimate. Shouldn't it be the other way around?"

You go my way, talking about the "likelihood of political instability…" as I suggest changing "low" for "high" and vice-versa. Here in your response, you change "stability" for "instability". I agree that in English "low" perceptions of the likelihood of political INSTABILITY and/or politically-motivated violence can go with a higher TLE but not "low" perceptions of the likelihood of political STABILITY…"

Reviewer #4: Review „Country- and individual-level factors associated with health working life expectancy"

I would like to thank the authors for the revision of the manuscript and the thorough responses to the numerous reviewer comments. Overall, the manuscript has been improved. However I would like to point some issues out that have to be amended before publication:

1) The quality of the language is not overall high. E.g., in the abstract, the first sentence lacks a verb and the first section titled background does not describe the background, but focuses rather on the aim of the study. Please let a native speaker read the manuscript. Further example is the sentence on line 13 page 6 of the manuscript, added as an answer to my comment - "Mortality information gained from exit…" - the sentence is not well written.

2) I would agree with the comment#13 of the reviewer 2 that the level of the TE and HWLE is unexpectedly high. I do not find a sufficient acknowledgment of this issue in the limitations section, although the authors mention this in the answer to this comment.

3) My comment 9 (reviewer 4 comment 9) was not answered completely. The question remains open where was the median value itself included - into the high or low-group? I insist on the answer to this question.

4) my comment 15 (reviewer 4) was not answered appropriately. I insist on including possible biases in mortality assessment into limitations.

Any attachments provided with reviews can be seen via the following link: [LINK]

--------------------------------------------------------- ---

General editorial requests:

(Note: not all will apply to your paper, but please check each item carefully)

*We ask every co-author listed on the manuscript to fill in a contributing author statement, making sure to declare all competing interests. If any of the co-authors have not filled in the statement, we will remind them to do so when the paper is revised. If all statements are not completed in a timely fashion this could hold up the re-review process. If new competing interests are declared later in the revision process, this may also hold up the submission. Should there be a problem getting one of your co-authors to fill in a statement we will be in contact. Please do not add or remove authors without first discussing this with the handling editor. You can see our competing interests policy here: http://journals.plos.org/plosmedicine/s/competing-interests.

*Please upload any figures associated with your paper as individual TIF or EPS files with 300dpi resolution at resubmission; please read our figure guidelines for more information on our requirements: http://journals.plos.org/plosmedicine/s/figures. While revising your submission, we strongly recommend that you use PLOS's NAAS tool (https://ngplosjournals.pagemajik.ai/artanalysis) to test your figure files. NAAS can convert your figure files to the TIFF file type and meet basic requirements (such as print size, resolution), or provide you with a report on issues that do not meet our requirements and that NAAS cannot fix.

After uploading your figures to PLOS's NAAS tool - https://ngplosjournals.pagemajik.ai/artanalysis, NAAS will process the files provided and display the results in the "Uploaded Files" section of the page as the processing is complete.

If the uploaded figures meet our requirements (or NAAS is able to fix the files to meet our requirements), the figure will be marked as "fixed" above. If NAAS is unable to fix the files, a red "failed" label will appear above.

When NAAS has confirmed that the figure files meet our requirements, please download the file via the download option, and include these NAAS processed figure files when submitting your revised manuscript.

*Please ensure that the paper adheres to the PLOS Data Availability Policy (see http://journals.plos.org/plosmedicine/s/data-availability), which requires that all data underlying the study's findings be provided in a repository or as Supporting Information. For data residing with a third party, authors are required to provide instructions with contact information (web or email address) for obtaining the data. Please note that a study author cannot be the contact person for the data. PLOS journals do not allow statements supported by "data not shown" or "unpublished results." For such statements, authors must provide supporting data or cite public sources that include it.

*We expect all researchers with submissions to PLOS in which author-generated code underpins the findings in the manuscript to make all author-generated code available without restrictions upon publication of the work. In cases where code is central to the manuscript, we may require the code to be made available as a condition of publication. Authors are responsible for ensuring that the code is reusable and well documented. Please make any custom code available, either as part of your data deposition or as a supplementary file. Please add a sentence to your data availability statement regarding any code used in the study, e.g. "The code used in the analysis is available from Github [URL] and archived in Zenodo [DOI link]" Please review our guidelines at https://journals.plos.org/plosmedicine/s/materials-software-and-code-sharing and ensure that your code is shared in a way that follows best practice and facilitates reproducibility and reuse. Because Github depositions can be readily changed or deleted, we encourage you to make a permanent DOI'd copy (e.g. in Zenodo) and provide the URL.

FORMATTING - GENERAL

*Abstract: Please structure your abstract using the PLOS Medicine headings (Background, Methods and Findings, Conclusions). Please combine the Methods and Findings sections into one section.

*At this stage, we ask that you include a short, non-technical Author Summary of your research to make findings accessible to a wide audience that includes both scientists and non-scientists. The Author Summary should immediately follow the Abstract in your revised manuscript. This text is subject to editorial change and should be distinct from the scientific abstract. Ideally each sub-heading should contain 2-3 single sentence, concise bullet points containing the most salient points from your study. In the final bullet point of 'What Do These Findings Mean?', please include the main limitations of the study in non-technical language. Please see our author guidelines for more information: https://journals.plos.org/plosmedicine/s/revising-your-manuscript#loc-author-summary.

*Please express the main results with 95% CIs as well as p values. When reporting p values please report as p<0.001 and where higher as the exact p value p=0.002, for example. Throughout, suggest reporting statistical information as follows to improve clarity for the reader "22% (95% CI [13%,28%]; p</=)". Please be sure to define all numerical values at first use.

*Please include page numbers and line numbers in the manuscript file. Use continuous line numbers (do not restart the numbering on each page).

*Please cite the reference numbers in square brackets. Citations should precede punctuation.

FIGURES AND TABLES

*Please provide titles and legends for all figures and tables (including those in Supporting Information files).

*Please define all abbreviations used in each figure/table (including those in Supporting Information files).

*Please consider avoiding the use of red and green in order to make your figure more accessible to those with color blindness.

SUPPLEMENTARY MATERIAL

*Please note that supplementary material will be posted as supplied by the authors. Therefore, please amend it according to the relevant comments outlined here.

*Please cite your Supporting Information as outlined here: https://journals.plos.org/plosmedicine/s/supporting-information

REFERENCES

*PLOS uses the numbered citation (citation-sequence) method and first six authors, et al.

*Please ensure that journal name abbreviations match those found in the National Center for Biotechnology Information (NCBI) databases (http://www.ncbi.nlm.nih.gov/nlmcatalog/journals), and are appropriately formatted and capitalised.

*Where website addresses are cited, please include the complete URL and specify the date of access (e.g. [accessed: 12/06/2023]).

*Please also see https://journals.plos.org/plosmedicine/s/submission-guidelines#loc-references for further details on reference formatting.

OBSERVATIONAL STUDIES

*Abstract: Please include the study design, population and setting, number of participants, years during which the study took place (enrollment and follow up), length of follow up, and main outcome measures.

*Please ensure that the study is reported according to the STROBE (or appropriate STOBE extension) guideline (available from: https://www.equator-network.org/reporting-guidelines/strobe) and include the completed STROBE (or STROBE extension) checklist as Supporting Information. Please add the following statement, or similar, to the Methods: "This study is reported as per the Strengthening the Reporting of Observational Studies in Epidemiology (STROBE) guideline (S1 Checklist)." When completing the checklist, please use section and paragraph numbers, rather than page numbers.

*POPULATION HEALTH/REGISTRY STUDIES: Please ensure that the study is reported according to the RECORD guideline (available from https://www.record-statement.org) and include the completed checklist as Supporting Information. Please add the following statement, or similar, to the Methods: "This study is reported as per the Reporting of Studies Conducted using Observational Routinely-Collected Data (RECORD) guideline (S1 Checklist)." When completing the checklist, please use section and paragraph numbers, rather than page numbers.

* POPULATION HEALTH ESTIMATES: Please ensure that the study is reported according to the GATHER statement (available from https://www.equator-network.org/reporting-guidelines/gather-statement) and include the completed checklist as Supporting Information. Please add the following statement, or similar, to the Methods: "This study is reported as per the Guidelines for Accurate and Transparent Health Estimates Reporting (GATHER) statement (S1 Checklist)." When completing the checklist, please use section and paragraph numbers, rather than page numbers.

*MEDIATION ANALYSES: We recommend that the study is reported according to the AGReMA statement (https://agrema-statement.org/#:~:text=AGReMA%20is%20an%20evidence%2D%20and,randomised%20trials%20and%20observational%20studies) and include the completed checklist as Supporting Information. Please add the following statement, or similar, to the Methods: "This study is reported as per the Guideline for Reporting Mediation Analyses (AGReMA) statement (S1 Checklist)." When completing the checklist, please use section and paragraph numbers, rather than page numbers.

*For all observational studies, in the manuscript text, please indicate: (1) the specific hypotheses you intended to test, (2) the analytical methods by which you planned to test them, (3) the analyses you actually performed, and (4) when reported analyses differ from those that were planned, transparent explanations for differences that affect the reliability of the study's results. If a reported analysis was performed based on an interesting but unanticipated pattern in the data, please be clear that the analysis was data driven.

*Please state in the Methods section whether the study had a prospective protocol or analysis plan. If a prospective analysis plan (from your funding proposal, IRB or other ethics committee submission, study protocol, or other planning document written before analyzing the data) was used in designing the study, please include the relevant document(s) with your revised manuscript as a Supporting Information file to be published alongside your study and cite it in the Methods section. A legend for this file should be included at the end of your manuscript. If no such document exists, please make sure that the Methods section transparently describes when analyses were planned, and when/why any data-driven changes to analyses took place. Changes in the analysis, including those made in response to peer review comments, should be identified as such in the Methods section of the paper, with rationale.

# Author comment: Author response to Decision Letter 3

**DOI:** 10.1371/journal.pmed.1004676.r005

24 Apr 2026

Attachment Submitted filename: Point-to-point response.docx

# Peer review: Decision Letter 3

**DOI:** 10.1371/journal.pmed.1004676.r006

**Authors:** Andreia Cunha

24 Jun 2026

Dear Dr Gao,

Sincere apologies for the delay in getting back to you with a decision which was due to challenges in securing all the necessary advice. Many thanks for submitting your manuscript "Country- and individual-level factors associated with healthy working life expectancy: a multinational longitudinal study" (PMEDICINE-D-25-02153R3) to PLOS Medicine. The paper has been reviewed by two of the original Reviewers, and the academic editor reviewed your response to Reviewer 2 who was unavailable; their comments are included below and can also be accessed here: [LINK]

As you will see, the two reviewers are mostly happy with the revision, but I'm afraid that our editorial view after discussing with the academic editor is that two of the more substantial concerns of Reviewer 2 have not been sufficiently addressed. After discussing the paper with the editorial team and an academic editor with relevant expertise, I'm pleased to invite you to revise the paper one more time to address the remaining concerns of Reviewer 2 with additonal analyses. We plan to send the revised paper to some or all of the original reviewers, and we cannot provide any guarantees at this stage regarding publication.

In addition to these revisions, you may need to complete some formatting changes, which you will receive in a follow up email. A member of our team will be in touch with a set of requests shortly. If you do not receive a separate email within a few days, please assume that checks have been completed, and no additional changes are required.

When you upload your revision, please include a point-by-point response that addresses all of the reviewer and editorial points, indicating the changes made in the manuscript and either an excerpt of the revised text or the location (eg: page and line number) where each change can be found. Please also be sure to check the general editorial comments at the end of this letter and include these in your point-by-point response. When you resubmit your paper, please include a clean version of the paper as the main article file and a version with changes tracked as a marked-up manuscript. It may also be helpful to check the guidelines for revised papers at http://journals.plos.org/plosmedicine/s/revising-your-manuscript for any that apply to your paper.

We ask that you submit your revision by Aug 24 2026 11:59PM. However, if this deadline is not feasible, please contact me by email, and we can discuss a suitable alternative.

Don't hesitate to contact me directly with any questions (acunha@plos.org).

Best regards,

Andreia

Andreia Cunha, PhD

Senior editor

PLOS Medicine

acunha@plos.org

-----------------------------------------------------------

Comments from the academic editor:

The authors responded to all 26 of Reviewer 2's comments and fully resolved the presentational and clarity ones — abstract now reports the range not averages, overstated and causal wording fixed, maps moved to the Supplement, "globally" removed, sensitivity methods explained, SHARE sample sizes added by country, and limitations expanded.

But the two concerns that drove Reviewer 2's Reject were met mostly with caveats and reframing rather than new analysis:

Estimate validity (Comments 14 & 19): The Switzerland (+~6 yrs) and Austria (+~5 yrs) TLE overestimation was acknowledged as a limitation and the framing shifted to "relative associations," but the bias was not quantified across countries or shown to be non-differential — while absolute HWLE values are still compared country-by-country throughout. Reviewer 2's actual question (is the bias uniform or differential?) is unanswered.

Novelty/depth (Comments 6, 7, 8, 21, 22): The aim was reframed around governance metrics, but the core finding (richer/better-governed countries have higher HWLE) is unchanged, and the specific deeper analysis requested — health conditions by country, workplace support, HnW/nHW/nHnW by country (Comment 22) — was not performed.

-----------------------------------------------------------

Comments from the reviewers:

Reviewer #1: The authors have addressed all my comments, and I am happy to recommend acceptance. I have only one final suggestion that is entirely optional and does not require a response:

Now that Method 1 to Method 7 in the Sensitivity Analysis section are clearly defined, it is not clear to me what scientific question Method 5 addresses. Reducing your sample size through random sampling will just increase the uncertainty of subsequent estimates (e.g., wider confidence intervals), and this is what is observed in the supplementary results. The authors may wish to clarify what Method 5 is testing that is not covered by the other sensitivity analyses or omit Method 5 altogether. That said, there is nothing incorrect with including it as-is. Either way, it looks like the upper bound of Method 5's "Not healthy and not working" 95% CI is a typo.

Well done and congratulations.

Reviewer #4: Dear authors,

thank you for the comprehensive revision and for responses to my comments. I do not have further questions and congratulate the authors to this successful and important analysis!

Best regards!

Any attachments provided with reviews can be seen via the following link: [LINK]

--------------------------------------------------------- ---

General editorial requests:

*We ask every co-author listed on the manuscript to fill in a contributing author statement, making sure to declare all competing interests. If any of the co-authors have not filled in the statement, we will remind them to do so when the paper is revised. If all statements are not completed in a timely fashion this could hold up the re-review process. If new competing interests are declared later in the revision process, this may also hold up the submission. Should there be a problem getting one of your co-authors to fill in a statement we will be in contact. Please do not add or remove authors without first discussing this with the handling editor. You can see our competing interests policy here: http://journals.plos.org/plosmedicine/s/competing-interests.

*Please upload any figures associated with your paper as individual TIF or EPS files with 300dpi resolution at resubmission; please read our figure guidelines for more information on our requirements: http://journals.plos.org/plosmedicine/s/figures. While revising your submission, we strongly recommend that you use PLOS's NAAS tool (https://ngplosjournals.pagemajik.ai/artanalysis) to test your figure files. NAAS can convert your figure files to the TIFF file type and meet basic requirements (such as print size, resolution), or provide you with a report on issues that do not meet our requirements and that NAAS cannot fix.

After uploading your figures to PLOS's NAAS tool - https://ngplosjournals.pagemajik.ai/artanalysis, NAAS will process the files provided and display the results in the "Uploaded Files" section of the page as the processing is complete.

If the uploaded figures meet our requirements (or NAAS is able to fix the files to meet our requirements), the figure will be marked as "fixed" above. If NAAS is unable to fix the files, a red "failed" label will appear above.

When NAAS has confirmed that the figure files meet our requirements, please download the file via the download option, and include these NAAS processed figure files when submitting your revised manuscript.

*Please ensure that the paper adheres to the PLOS Data Availability Policy (see http://journals.plos.org/plosmedicine/s/data-availability), which requires that all data underlying the study's findings be provided in a repository or as Supporting Information. For data residing with a third party, authors are required to provide instructions with contact information (web or email address) for obtaining the data. Please note that a study author cannot be the contact person for the data. PLOS journals do not allow statements supported by "data not shown" or "unpublished results." For such statements, authors must provide supporting data or cite public sources that include it.

*We expect all researchers with submissions to PLOS in which author-generated code underpins the findings in the manuscript to make all author-generated code available without restrictions upon publication of the work. In cases where code is central to the manuscript, we require the code to be made available as a condition of publication. Authors are responsible for ensuring that the code is reusable and well documented. Please make any custom code available, either as part of your data deposition or as a supplementary file. Please add a sentence to your data availability statement regarding any code used in the study, e.g. "The code used in the analysis is available from Github [URL] and archived in Zenodo [DOI link]" Please review our guidelines at https://journals.plos.org/plosmedicine/s/materials-software-and-code-sharing and ensure that your code is shared in a way that follows best practice and facilitates reproducibility and reuse. Because Github depositions can be readily changed or deleted, we encourage you to make a permanent DOI'd copy (e.g. in Zenodo) and provide the URL.

*The funding statement should include: specific grant numbers, initials of authors who received each award, URLs to sponsors’ websites. Also, please state whether any sponsors or funders (other than the named authors) played any role in study design, data collection and analysis, the decision to publish, or preparation of the manuscript. If they had no role in the research, include this sentence: “The funders had no role in study design, data collection and analysis, decision to publish, or preparation of the manuscript.”

*All authors must declare their relevant competing interests per the PLOS policy, which can be seen here: https://journals.plos.org/plosmedicine/s/competing-interests For authors with ties to industry, please indicate whether any of the interests has a financial stake in the results of the current study.

*Please specify whether informed consent was obtained, and if it was waived or not necessary, please explain why. Please ensure that the research complies with the PLOS policy in full: https://journals.plos.org/plosmedicine/s/human-subjects-research#loc-patient-privacy-and-informed-consent-for-publication

*Please ensure that the study is reported according to the STROBE guideline and include the completed STROBE checklist as Supporting Information. When completing the checklist, please use section and paragraph numbers, rather than page numbers. Please add the following statement, or similar, to the Methods: "This study is reported as per STROBE guideline (S1 Checklist)."

* Please disclose any use of AI tools and technologies to the study or article content at the end of the Methods. Please specify the name(s) and version(s) of any tool(s) used and a description of their use, including how the AI-assisted or generated content was evaluated by authors, and a clear statement of which aspects of the study, article contents, data, or supporting files were affected/generated by AI tool usage. Please also include a statement specifying who retains responsibility for the final manuscript. See our guidelines for more information: https://journals.plos.org/plosmedicine/s/ethical-publishing-practice#loc-artificial-intelligence-tools-and-technologies.

FORMATTING - GENERAL

* Please confirm that your title complies with PLOS Medicine's style. Your title must be nondeclarative and not a question. It should begin with main concept if possible. "Effect of" should be used only if causality can be inferred, i.e., for an RCT. Please place the study design ("A randomized controlled trial," "A retrospective study," "A modelling study," etc.) in the subtitle (ie, after a colon).

*Abstract: Please structure your abstract using the PLOS Medicine headings (Background, Methods and Findings, Conclusions). Please combine the Methods and Findings sections into one section.

*At this stage, we ask that you include a short, non-technical Author Summary of your research to make findings accessible to a wide audience that includes both scientists and non-scientists. The Author Summary should immediately follow the Abstract in your revised manuscript. This text is subject to editorial change and should be distinct from the scientific abstract. Ideally each sub-heading should contain 2-3 single sentence, concise bullet points containing the most salient points from your study. In the final bullet point of 'What Do These Findings Mean?', please include the main limitations of the study in non-technical language. Please see our author guidelines for more information: https://journals.plos.org/plosmedicine/s/revising-your-manuscript#loc-author-summary.

*Please express the main results with 95% CIs as well as p values. When reporting p values please report as p<0.001 and where higher as the exact p value p=0.002, for example. Throughout, suggest reporting statistical information as follows to improve clarity for the reader "22% (95% CI [13%,28%]; p</=)". Please be sure to define all numerical values at first use.

*Please include page numbers and line numbers in the manuscript file. Use continuous line numbers (do not restart the numbering on each page).

*Please cite the reference numbers in square brackets. Citations should precede punctuation.

FIGURES AND TABLES

*Please provide titles and legends for all figures and tables (including those in Supporting Information files).

*Please define all abbreviations used in each figure/table (including those in Supporting Information files).

*Please consider avoiding the use of red and green in order to make your figure more accessible to those with color blindness.

SUPPLEMENTARY MATERIAL

*Please note that supplementary material will be posted as supplied by the authors. Therefore, please amend it according to the relevant comments outlined here.

*Please cite your Supporting Information as outlined here: https://journals.plos.org/plosmedicine/s/supporting-information

REFERENCES

*PLOS uses the numbered citation (citation-sequence) method and first six authors, et al.

*Please ensure that journal name abbreviations match those found in the National Center for Biotechnology Information (NCBI) databases (http://www.ncbi.nlm.nih.gov/nlmcatalog/journals), and are appropriately formatted and capitalised.

*Where website addresses are cited, please include the complete URL and specify the date of access (e.g. [accessed: 12/06/2023]).

*Please also see https://journals.plos.org/plosmedicine/s/submission-guidelines#loc-references for further details on reference formatting.

OBSERVATIONAL STUDIES

*Abstract: Please include the study design, population and setting, number of participants, years during which the study took place (enrollment and follow up), length of follow up, and main outcome measures.

*Please ensure that the study is reported according to the STROBE (or appropriate STOBE extension) guideline (available from: https://www.equator-network.org/reporting-guidelines/strobe) and include the completed STROBE (or STROBE extension) checklist as Supporting Information. Please add the following statement, or similar, to the Methods: "This study is reported as per the Strengthening the Reporting of Observational Studies in Epidemiology (STROBE) guideline (S1 Checklist)." When completing the checklist, please use section and paragraph numbers, rather than page numbers.

*FOR POPULATION HEALTH/REGISTRY STUDIES Please ensure that the study is reported according to the RECORD guideline (available from https://www.record-statement.org) and include the completed checklist as Supporting Information. Please add the following statement, or similar, to the Methods: "This study is reported as per the Reporting of Studies Conducted using Observational Routinely-Collected Data (RECORD) guideline (S1 Checklist)." When completing the checklist, please use section and paragraph numbers, rather than page numbers.

*FOR POPULATION HEALTH ESTIMATES Please ensure that the study is reported according to the GATHER statement (available from https://www.equator-network.org/reporting-guidelines/gather-statement) and include the completed checklist as Supporting Information. Please add the following statement, or similar, to the Methods: "This study is reported as per the Guidelines for Accurate and Transparent Health Estimates Reporting (GATHER) statement (S1 Checklist)." When completing the checklist, please use section and paragraph numbers, rather than page numbers.

*FOR MEDIATION ANALYSES We recommend that the study is reported according to the AGReMA statement (https://agrema-statement.org/#:~:text=AGReMA%20is%20an%20evidence%2D%20and,randomised%20trials%20and%20observational%20studies) and include the completed checklist as Supporting Information. Please add the following statement, or similar, to the Methods: "This study is reported as per the Guideline for Reporting Mediation Analyses (AGReMA) statement (S1 Checklist)." When completing the checklist, please use section and paragraph numbers, rather than page numbers.

*For all observational studies, in the manuscript text, please indicate: (1) the specific hypotheses you intended to test, (2) the analytical methods by which you planned to test them, (3) the analyses you actually performed, and (4) when reported analyses differ from those that were planned, transparent explanations for differences that affect the reliability of the study's results. If a reported analysis was performed based on an interesting but unanticipated pattern in the data, please be clear that the analysis was data driven.

*Please state in the Methods section whether the study had a prospective protocol or analysis plan. If a prospective analysis plan (from your funding proposal, IRB or other ethics committee submission, study protocol, or other planning document written before analyzing the data) was used in designing the study, please include the relevant document(s) with your revised manuscript as a Supporting Information file to be published alongside your study and cite it in the Methods section. A legend for this file should be included at the end of your manuscript. If no such document exists, please make sure that the Methods section transparently describes when analyses were planned, and when/why any data-driven changes to analyses took place. Changes in the analysis, including those made in response to peer review comments, should be identified as such in the Methods section of the paper, with rationale.

# Author comment: Author response to Decision Letter 4

**DOI:** 10.1371/journal.pmed.1004676.r007

20 Jul 2026

Attachment Submitted filename: Responses to Reviewers.docx

# Editor report: Decision Letter 4

**DOI:** 10.1371/journal.pmed.1004676.r008

**Authors:** Andreia Cunha

18 Aug 2026

Dear Dr. Gao,

My apologies for the delay in getting back to you with a decision and thank you very much for re-submitting your manuscript "Country- and individual-level factors associated with healthy working life expectancy: a multinational longitudinal study" (PMEDICINE-D-25-02153R4) for review by PLOS Medicine.

I have discussed the paper and revisions with my colleagues and the academic editor. I am pleased to say that provided the remaining editorial and production issues are dealt with we are planning to accept the paper for publication in the journal.

The remaining issues that need to be addressed are listed at the end of this email. Any accompanying reviewer attachments can be seen via the link below. Please take these into account before resubmitting your manuscript:

[LINK]

***Please note while forming your response, if your article is accepted, you may have the opportunity to make the peer review history publicly available. The record will include editor decision letters (with reviews) and your responses to reviewer comments. If eligible, we will contact you to opt in or out.***

In revising the manuscript for further consideration here, please ensure you address the specific points made by each reviewer and the editors. In your rebuttal letter you should indicate your response to the reviewers' and editors' comments and the changes you have made in the manuscript. Please submit a clean version of the paper as the main article file. A version with changes marked must also be uploaded as a marked up manuscript file.

Please also check the guidelines for revised papers at http://journals.plos.org/plosmedicine/s/revising-your-manuscript for any that apply to your paper. If you haven't already, we ask that you provide a short, non-technical Author Summary of your research to make findings accessible to a wide audience that includes both scientists and non-scientists. The Author Summary should immediately follow the Abstract in your revised manuscript. This text is subject to editorial change and should be distinct from the scientific abstract.

In addition to these revisions, you may need to complete some formatting changes, which you will receive in a follow up email. A member of our team will be in touch with a set of requests shortly. If you do not receive a separate email within a few days, please assume that checks have been completed, and no additional changes are required.

We expect to receive your revised manuscript within 1 week. Please email us (plosmedicine@plos.org) if you have any questions or concerns.

We ask every co-author listed on the manuscript to fill in a contributing author statement. If any of the co-authors have not filled in the statement, we will remind them to do so when the paper is revised. If all statements are not completed in a timely fashion this could hold up the re-review process. Should there be a problem getting one of your co-authors to fill in a statement we will be in contact. YOU MUST NOT ADD OR REMOVE AUTHORS UNLESS YOU HAVE ALERTED THE EDITOR HANDLING THE MANUSCRIPT TO THE CHANGE AND THEY SPECIFICALLY HAVE AGREED TO IT.

Please ensure that the paper adheres to the PLOS Data Availability Policy (see http://journals.plos.org/plosmedicine/s/data-availability), which requires that all data underlying the study's findings be provided in a repository or as Supporting Information. For data residing with a third party, authors are required to provide instructions with contact information for obtaining the data. PLOS journals do not allow statements supported by "data not shown" or "unpublished results." For such statements, authors must provide supporting data or cite public sources that include it.

To enhance the reproducibility of your results, we recommend that you deposit your laboratory protocols in protocols.io, where a protocol can be assigned its own identifier (DOI) such that it can be cited independently in the future. Additionally, PLOS ONE offers an option to publish peer-reviewed clinical study protocols. Read more information on sharing protocols at https://plos.org/protocols?utm_medium=editorial-email&utm_source=authorletters&utm_campaign=protocols

Please review your reference list to ensure that it is complete and correct. If you have cited papers that have been retracted, please include the rationale for doing so in the manuscript text, or remove these references and replace them with relevant current references. Any changes to the reference list should be mentioned in the rebuttal letter that accompanies your revised manuscript.

Please note, when your manuscript is accepted, an uncorrected proof of your manuscript will be published online ahead of the final version, unless you've already opted out via the online submission form. If, for any reason, you do not want an earlier version of your manuscript published online or are unsure if you have already indicated as such, please let the journal staff know immediately at plosmedicine@plos.org.

If you have any questions in the meantime, please contact me or the journal staff on plosmedicine@plos.org.

We look forward to receiving the revised manuscript by Aug 18 2026 11:59PM.

Sincerely,

Andreia Cunha, PhD

Senior Editor

PLOS Medicine

plosmedicine.org

------------------------------------------------------------

Requests from Editors:

GENERAL EDITORIAL REQUESTS

* At this stage, we ask that you include a short, non-technical Author Summary of your research to make findings accessible to a wide audience that includes both scientists and non-scientists. The Author Summary should immediately follow the Abstract in your revised manuscript. This text is subject to editorial change and should be distinct from the scientific abstract. Ideally each sub-heading should contain 2-3 single sentence, concise bullet points containing the most salient points from your study. In the final bullet point of ‘What Do These Findings Mean?’ Please include the main limitations of the study in non-technical language.

Please see our author guidelines for more information: https://journals.plos.org/plosmedicine/s/revising-your-manuscript#loc-author-summary.

* Please confirm that your title complies with PLOS Medicine's style. Your title must be nondeclarative and not a question. It should begin with main concept if possible. "Effect of" should be used only if causality can be inferred, i.e., for an RCT. Please place the study design ("A randomized controlled trial," "A retrospective study," "A modelling study," etc.) in the subtitle (ie, after a colon).

* Please confirm that your abstract complies with our requirements, including format (three sections: Background, Methods and Findings, and Conclusions) and providing all the information relevant to this study type https://journals.plos.org/plosmedicine/s/submission-guidelines#loc-abstract

* Please ensure that the Introduction ends with a clear description of the study question or hypothesis.

* Please ensure that all abbreviations are defined at first use throughout the text.

* Please confirm that all numbers presented in the abstract are present and identical to numbers presented in the main manuscript text.

GENERAL

* Please include a statement regarding use or non-use of AI tools and technologies at the end of the Methods. Please specify the name(s) and version(s) of any tool(s) used and a description of their use, including how the AI-assisted or generated content was evaluated by authors, and a clear statement of which aspects of the study, article contents, data, or supporting files were affected or generated by AI tool usage. Please also include a statement specifying who retains responsibility for the final manuscript. See our guidelines for more information: https://journals.plos.org/plosmedicine/s/ethical-publishing-practice#loc-artificial-intelligence-tools-and-technologies.

* Please review your text for claims of novelty or primacy (e.g. 'for the first time') and remove this language. In addition, please check that any use of statistical terms (such as trend or significant) are supported by the data, and if not please remove them.

* Statistical reporting: Please revise throughout the manuscript, including tables and figures.

- Please report statistical information as follows to improve clarity for the reader ""22% (95% CI [13,28]; p</=)"".

- Please separate upper and lower bounds with commas instead of hyphens as the latter can be confused with reporting of negative values.

- Please repeat statistical definitions (HR, CI etc.) for each set of parentheses.

* In the last sentence of the Abstract Methods and Findings section, please describe the main limitation(s) of the study's methodology.

* In the abstract, please include the important dependent variables that are adjusted for in the analyses.

* In the author summary, in the final bullet point of 'What Do These Findings Mean?', please include the main limitations of the study in non-technical language.

FUNDING STATEMENT

* The funding statement should include: specific grant numbers, initials of authors who received each award, URLs to sponsors’ websites. Also, please state whether any sponsors or funders (other than the named authors) played any role in study design, data collection and analysis, the decision to publish, or preparation of the manuscript. If they had no role in the research, include this sentence: “The funders had no role in study design, data collection and analysis, decision to publish, or preparation of the manuscript.”

* It appears that one or more study authors is affiliated with one or more of the agencies that funded the study (Huadong Hospital). Thus, the statement “The funders had no role in study design, data collection and analysis, decision to publish, or preparation of the manuscript” does not apply. Please revise the Financial Disclosure accordingly, as in "[Author name] is [author's role] at [funding agency]. The funders had no other role in study design…..”

COMPETING INTERESTS STATEMENT

* All authors must declare their relevant competing interests per the PLOS policy, which can be seen here: https://journals.plos.org/plosmedicine/s/competing-interests For authors with ties to industry, please indicate whether any of the interests has a financial stake in the results of the current study.

*Please amend your competing interests statement. Funding information should go to the relevant section and include URLs.

DATA AVAILABILITY

* PLOS defines the “minimal data set” to consist of the data set used to reach the conclusions drawn in the manuscript with related metadata and methods, and any additional data required to replicate the reported study findings in their entirety. Authors do not need to submit their entire data set, or the raw data collected during an investigation. Please submit the following data:

The values behind the means, standard deviations and other measures reported;

The values used to build graphs;

The points extracted from images for analysis.

* The Data Availability Statement (DAS) requires revision. For each data source used in your study:

a) If the data are freely or publicly available, note this and state the location of the data: within the paper, in Supporting Information files, or in a public repository (include the DOI or accession number).

b) If the data are owned by a third party but freely available upon request, please note this and state the institutional owner of the data set and contact information for data requests (institutional web or email address). Note that a study author cannot be the contact for the data.

c) If the data are not freely available, please describe briefly the ethical, legal, or contractual restriction that prevents you from sharing it. Please also include an appropriate institutional contact (institutional web or email address) for inquiries (again, this cannot be a study author).

ETHICS AND CONSENT

* Please specify whether informed consent was obtained. If informed consent was not required or waived please explain why in the ethics and consent statement. Please ensure that the research complies with the PLOS policy in full: https://journals.plos.org/plosmedicine/s/human-subjects-research#loc-patient-privacy-and-informed-consent-for-publication

FIGURES

* Please define all elements of box plots in the figure caption - center line, box limits and whiskers.

* Please provide titles and legends for all figures and tables (including those in Supporting Information files). Please define all acronyms used in each figure or table in its corresponding legend.

* Please ensure that where relevant figures include 95% CIs.

* When a p value is given, please specify the statistical test used to determine it in the legend.

* Please consider avoiding the use of red and green in order to make your figure more accessible

* Please provide the unadjusted comparisons as well as the adjusted comparisons in all relevant Tables

* Please specify the variables controlled for in all relevant Tables

ACKNOWLEDGMENTS

* Please include an Acknowledgments section in your manuscript. The Acknowledgements should not include funder information.

OBSERVATIONAL STUDIES

* Abstract: Please include the study design, population and setting, number of participants, years during which the study took place (enrollment and follow up), length of follow up, and main outcome measures.

* Please ensure that the study is reported according to the STROBE (or appropriate STOBE extension) guideline (available from: https://www.equator-network.org/reporting-guidelines/strobe) and include the completed STROBE (or STROBE extension) checklist as Supporting Information. Please add the following statement, or similar, to the Methods: "This study is reported as per the Strengthening the Reporting of Observational Studies in Epidemiology (STROBE) guideline (S1 Checklist)." When completing the checklist, please use section and paragraph numbers, rather than page numbers.

* FOR POPULATION HEALTH/REGISTRY STUDIES: Please ensure that the study is reported according to the RECORD guideline (available from https://www.record-statement.org) and include the completed checklist as Supporting Information. Please add the following statement, or similar, to the Methods: "This study is reported as per the Reporting of Studies Conducted using Observational Routinely-Collected Data (RECORD) guideline (S1 Checklist)." When completing the checklist, please use section and paragraph numbers, rather than page numbers.

* FOR POPULATION HEALTH ESTIMATES: Please ensure that the study is reported according to the GATHER statement (available from https://www.equator-network.org/reporting-guidelines/gather-statement) and include the completed checklist as Supporting Information. Please add the following statement, or similar, to the Methods: "This study is reported as per the Guidelines for Accurate and Transparent Health Estimates Reporting (GATHER) statement (S1 Checklist)." When completing the checklist, please use section and paragraph numbers, rather than page numbers.

* FOR MEDIATION ANALYSES: We recommend that the study is reported according to the AGReMA statement (https://agrema-statement.org/#:~:text=AGReMA%20is%20an%20evidence%2D%20and,randomised%20trials%20and%20observational%20studies) and include the completed checklist as Supporting Information. Please add the following statement, or similar, to the Methods: "This study is reported as per the Guideline for Reporting Mediation Analyses (AGReMA) statement (S1 Checklist)." When completing the checklist, please use section and paragraph numbers, rather than page numbers.

* For all observational studies, in the manuscript text, please indicate: (1) the specific hypotheses you intended to test, (2) the analytical methods by which you planned to test them, (3) the analyses you actually performed, and (4) when reported analyses differ from those that were planned, transparent explanations for differences that affect the reliability of the study's results. If a reported analysis was performed based on an interesting but unanticipated pattern in the data, please be clear that the analysis was data driven.

* Did your study have a prospective protocol or analysis plan? Please state this (either way) early in the Methods section.

a) If a prospective analysis plan (from your funding proposal, IRB or other ethics committee submission, study protocol, or other planning document written before analyzing the data) was used in designing the study, please include the relevant prospectively written document with your revised manuscript as a Supporting Information file to be published alongside your study, and cite it in the Methods section. A legend for this file should be included at the end of your manuscript.

b) If no such document exists, please make sure that the Methods section transparently describes when analyses were planned, and when/why any data-driven changes to analyses took place.

c) In either case, changes in the analysis-- including those made in response to peer review comments-- should be identified as such in the Methods section of the paper, with rationale.

* Your study is observational and therefore causality cannot be inferred. Please remove language that implies causality and refer to associations instead.

Any attachments provided with reviews can be seen via the following link:

[LINK]

# Author comment: Author response to Decision Letter 5

**DOI:** 10.1371/journal.pmed.1004676.r009

23 Aug 2026

Attachment Submitted filename: Responses to Editors.docx

# Editor report: Decision Letter 5

**DOI:** 10.1371/journal.pmed.1004676.r010

**Authors:** Andreia Cunha

25 Aug 2026

Dear Dr Gao,

On behalf of my colleagues and the Academic Editor, [AE Name], I am pleased to inform you that we have agreed to publish your manuscript "Country- and individual-level factors associated with healthy working life expectancy: a multinational longitudinal study" (PMEDICINE-D-25-02153R5) in PLOS Medicine.

Before your manuscript can be formally accepted you will need to complete some formatting changes, which you will receive in a follow up email.

* In the Au summary, please revise following sentence as suggested: 'This study provides a comprehensive estimate of HWLE at age 50, and identifies national economic indicators, governance quality and employment-related conditions as contextual factors associated with HWLE, beyond individual factors like chronic diseases and lifestyle.'

* In the Methods section, please indicate the reason that informed consent was not obtained (e.g. the data were analyzed anonymously). You have only provided information about ethics approval but not about informed consent.

* In the Data Availability Statement, please:

- state whether the data was de-identified and analysed anonymously.

- Include a citable digital object identifier (DOI) or other persistent identifier of the code (such as through linkage with Zenodo) in the manuscript’s.

- add the statement: The minimal dataset underlying the reported results have been provided in the Supporting Information.

- update the DAS in the submission system to be the same as the manuscript

Please be aware that it may take several days for you to receive this email; during this time no action is required by you. Once you have received these formatting requests, please note that your manuscript will not be scheduled for publication until you have made the required changes.

In the meantime, please log into Editorial Manager at http://www.editorialmanager.com/pmedicine/, click the "Update My Information" link at the top of the page, and update your user information to ensure an efficient production process.

PRESS

We frequently collaborate with press offices. If your institution or institutions have a press office, please notify them about your upcoming paper at this point, to enable them to help maximise its impact. If the press office is planning to promote your findings, we would be grateful if they could coordinate with medicinepress@plos.org. If you have not yet opted out of the early version process, we ask that you notify us immediately of any press plans so that we may do so on your behalf.

We also ask that you take this opportunity to read our Embargo Policy regarding the discussion, promotion and media coverage of work that is yet to be published by PLOS. As your manuscript is not yet published, it is bound by the conditions of our Embargo Policy. Please be aware that this policy is in place both to ensure that any press coverage of your article is fully substantiated and to provide a direct link between such coverage and the published work. For full details of our Embargo Policy, please visit http://www.plos.org/about/media-inquiries/embargo-policy/.

To enhance the reproducibility of your results, we recommend that you deposit your laboratory protocols in protocols.io, where a protocol can be assigned its own identifier (DOI) such that it can be cited independently in the future. Additionally, PLOS ONE offers an option to publish peer-reviewed clinical study protocols. Read more information on sharing protocols at https://plos.org/protocols?utm_medium=editorial-email&utm_source=authorletters&utm_campaign=protocols

Thank you again for submitting to PLOS Medicine. We look forward to publishing your paper.

Sincerely,

Andreia Cunha, PhD

Senior Editor

PLOS Medicine
